# Nutrient control of eukaryote cell growth: a systems biology study in yeast

**DOI:** 10.1186/1741-7007-8-68

**Published:** 2010-05-24

**Authors:** Alex Gutteridge, Pınar Pir, Juan I Castrillo, Philip D Charles, Kathryn S Lilley, Stephen G Oliver

**Affiliations:** 1 Cambridge Systems Biology Centre & Department of Biochemistry, University of Cambridge, Sanger Building, 80 Tennis Court Road, Cambridge CB2 1GA, UK

## Abstract

**Background:**

To elucidate the biological processes affected by changes in growth rate and nutrient availability, we have performed a comprehensive analysis of the transcriptome, proteome and metabolome responses of chemostat cultures of the yeast, *Saccharomyces cerevisiae*, growing at a range of growth rates and in four different nutrient-limiting conditions.

**Results:**

We find significant changes in expression for many genes in each of the four nutrient-limited conditions tested. We also observe several processes that respond differently to changes in growth rate and are specific to each nutrient-limiting condition. These include carbohydrate storage, mitochondrial function, ribosome synthesis, and phosphate transport. Integrating transcriptome data with proteome measurements allows us to identify previously unrecognized examples of post-transcriptional regulation in response to both nutrient and growth-rate signals.

**Conclusions:**

Our results emphasize the unique properties of carbon metabolism and the carbon substrate, the limitation of which induces significant changes in gene regulation at the transcriptional and post-transcriptional level, as well as altering how many genes respond to growth rate. By comparison, the responses to growth limitation by other nutrients involve a smaller set of genes that participate in specific pathways.

See associated commentary http://www.biomedcentral.com/1741-7007/8/62

## Background

Growth (that is, the increase of biomass due to macromolecular synthesis) constitutes a fundamental process in the living cell. It results from the catabolism of available nutrients, yielding metabolic intermediates and energy for the synthesis of cellular constituents. In order to be able to survive in a variety of different environments, a unicellular microbe must be able to regulate the myriad pathways that lead to growth in response to the external nutrient supply [1-4].

The model eukaryote, *Saccharomyces cerevisiae *[5], has been used extensively to investigate the processes involved in sensing and assimilating nutrients and cell growth. Recently, studies have been made integrating nutrient and growth rate effects on the metabolome level [6], whereas previous studies have examined the genes and processes regulating cell growth without making any detailed analysis of specific nutrient effects [7-9] or individual nutrient responses [10,11]. Here, we present a comprehensive, detailed analysis integrating both processes at the transcriptome, proteome, and metabolome levels. This (to our knowledge) has not been attempted to date.

One difficulty with making analyses of complex systems, such as those governing growth, is that regulation of the activity of a protein or pathway can occur at multiple levels in the cellular machinery. At the transcriptional level, transcription factors and other elements control the expression of genes [12], while many other mechanisms control activities post-transcriptionally [13-15]. To date, transcriptional regulation has been the focus of most studies of nutrient and growth rate responses, due to the ease of gene expression analysis using microarray [16,17] or deep-sequencing [18] technologies. However, the importance of regulation at the proteome and metabolome levels means that integrative studies incorporating multiple types of data are necessary [7,19-23]. Another important feature of many studies is the use of defined controlled conditions, of which chemostat fermentors are an example [24-27], to ensure time-course and steady-state measurements are taken under rigorously defined conditions, making comparisons between experiments more robust [28,29].

In a previous study [7], we characterized a *core *set of genes, proteins and metabolic pathways subject to control by cell growth rate, irrespective of the specific nutrient limitation by which the different growth rates were imposed. In this work, we aim to use our comprehensive transcriptome, proteome and metabolome data to examine the mechanisms by which *S. cerevisiae*, as a prototypic eukaryotic cell, adapts its intracellular networks to support cell growth under each specific nutrient-limiting condition.

## Results and discussion

The transcriptome and metabolome data presented here are as used previously (see [7] for details), while the proteome data comes from a reanalysis of the existing mass spectra using updated techniques. In brief, *Saccharomyces cerevisiae *was grown under four different nutrient limitations (glucose, ammonium, phosphate, and sulphate) at three different dilution rates (D = μ = 0.07 h^-1^, 0.1 h^-1^, and 0.2 h^-1^). Gene expression at the mRNA level was investigated by transcriptome analysis using Affymetrix hybridization arrays. Proteomic studies were performed using isotope tags for multiplexed relative and absolute quantification (iTRAQ). In this case, the four tags and labeling schema applied (see [7] for details) allowed us to test and compare the proteomes of cells grown at μ = 0.1 h^-1 ^with those of cells grown at μ = 0.2 h^-1 ^for all four nutrient limitations. For the metabolome, gas chromatography coupled to time-of-flight mass spectrometry (GC/TOF-MS) was used to analyze the complement of intracellular and extracellular metabolites, that is, the endo- and the exometabolomes [7]. All data are publicly available at the Manchester Centre for Integrative Systems Biology http://www.mcisb.org.

### Nutrient-specific responses at the transcriptome, proteome and metabolome levels

**Figure 1:**
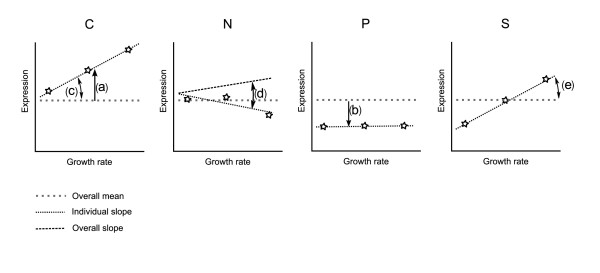
**Graphical representation of the parameters used to detect genes with nutrient and growth-rate effects**. An overall mean expression level (grey dotted line) and linear regression (black dashed line shown only for nitrogen limitation) is calculated across all four conditions. Separate means and linear regressions are then made for each condition (C, N, P and S) separately and compared. In this example we highlight significant nutrient effects (of opposite signs) in carbon **(a) **and phosphate **(b) **limitations, significant growth-rate effects in carbon **(c) **and sulphur **(e) **limitations (the slopes are significantly different from 0) and nutrient-specific growth-rate effects in nitrogen **(d) **limitation (slope is significantly different from overall slope).

Initially, genes were selected and analyzed based on transcriptional changes between the different limiting nutrients, while growth-rate effects were ignored. The mean expression level for each gene-nutrient pair was compared to the mean expression level across all nutrient limitations for the given gene. This parameter and the other parameters used later to find growth rate regulated genes are shown graphically in Figure 1. With the experimental conditions used here, this parameter allows us to compare nutrient-limited with nutrient excess conditions rather than nutrient presence to nutrient absence, as has been done previously. Table 1 shows the number of genes significantly up and down regulated in each nutrient limiting condition detected using this analysis (false discovery rate, FDR, < 5%).

**Table 1:** Number of genes significantly up or down regulated under each nutrient limitation (FDR < 5%).

	Carbon	Nitrogen	Phosphorus	Sulphur
**Up-regulated**	905	67	65	56
**Down-regulated**	390	49	195	72
**Total**	1295	116	260	128

While nitrogen-, phosphorus- and sulphur-limitation led to similar numbers of differentially expressed genes, the carbon-limited state triggered a much broader transcriptional response. Genes whose expression is up-regulated in carbon-limitation dominate, most likely as an effect of the release of glucose repression [30-32].

**Figure 2:**
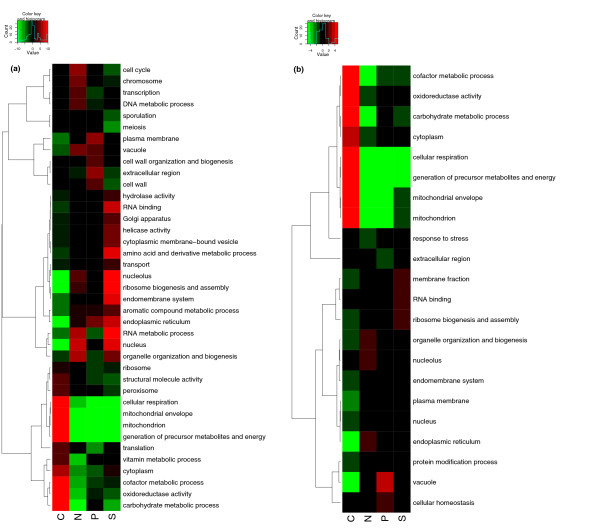
**GO_slim analysis of functional categories up- and down-regulated in each nutrient-limitation and detected at the (a) transcript and (b) protein levels**. Colours indicate the significance of the association of the given GO term with up- (red) or down- (green) regulation in a given nutrient-limitation relative to the other three. *Value *in the colour key is defined as -log.10(*P*) for up-regulated terms, and log.10(*P) *for down-regulated terms. Only GO_slim categories that showed a significant nutrient effect (FDR < 0.01) were included. The dendrogram showing the relationship between the terms is calculated based on the correlation coefficient between each pair of terms followed by complete linkage hierarchical clustering.

A Gene Ontology (GO) slim [33] analysis was made to get a global picture of the functions of the genes involved in these transcriptional changes. Functional analysis was performed using a cutoff-free method similar to LRpath [34] that identifies which GO terms annotate genes whose expression showed a significant tendency to be up- or down-regulated in each nutrient limitation; all *P *values quoted are corrected for multiple testing using the method of Benjamini and Hochberg [35]. Figure 2(a) shows the association of each GO slim functional category with up and down regulation at the transcript level in each nutrient limitation.

To complement the transcriptome analysis, we performed a similar analysis using the proteome data. Peptides corresponding to 1,869 open reading frames (ORFs) were detected. We found that the correlation of the protein-level changes with those of the corresponding mRNAs was significant, though weak, in each of the four conditions; although it is considerably weaker in carbon limitation (R: Carbon = 0.44, Nitrogen = 0.60, Phosphorus = 0.55, Sulphur = 0.59; P < 1 × 10^-16 ^in all cases), showing the relevance of post-transcriptional control of cell growth. Plots of these correlations are shown in Additional Files 1, 2, 3, 4. We also calculated the fold change in protein levels for each detected ORF between each nutrient-limiting condition and the overall mean level across all conditions. The GO_slim analysis performed on these data is shown in Figure 2(b).

The specific response to carbon limitation is reflected in the transcriptional and proteomic patterns shown in Figure 2, together with its interrelationship with sulphur metabolism. Cellular respiration, mitochondrial functions and oxidoreductase activity are all significantly up-regulated under carbon limitation relative to carbon excess. The GO terms that appear most significantly down-regulated under this condition are nucleolar and ribosomal terms such as *ribosome biogenesis and assembly*. The same biological processes also appear significantly up-regulated under sulphur limitation, along with nucleus, nucleolar, and amino-acid related terms. These situations point to a role for carbon and sulphur availability in regulating ribosomal biogenesis. The fact that genes involved in ribosomal biogenesis and amino acid derivative processes appear up-regulated under sulphur limitation also points to the induction of mechanisms to overcome detrimental effects than may occur when sulphur is scarce, such as biosynthesis of methionine, methionyl-tRNA and S-adenosylmethionine (SAM, AdoMet) and ribosomal RNA methylations.

Under nitrogen limitation, genes associated with nuclear activities appear up-regulated, along with those in the functional categories *cell cycle*, *organelle organization and biogenesis*, and *vacuole*. Genes involved in vacuolar functions are also up-regulated under phosphorus limitation, which points to a role for these organelles in the redistribution of intracellular amino acid and polyphosphate pools under these conditions. As expected, mitochondrial functions and genes involved in cellular respiration towards generation of energy appear significantly down-regulated under phosphorus limitation. Terms relating to proteins involved in signal transduction (for example, kinases and phosphatases) do not appear to be significantly up- or down-regulated at the transcriptional level, which does not exclude their being regulated post-transcriptionally.

To gain further insight into these results, we performed a full functional analysis on the transcriptome and proteome data using GO [33], Kegg [36] and Yeastract [37] annotations to identify the functional classes, pathways, and transcription factors associated with up- and down-regulation in each nutrient-limiting condition. The full list of significant GO terms, Kegg pathways, and transcription factors are shown in Additional Files 5, 6, 7, 8, 9, 10, 11, 12, 13, 14.

#### Carbon limitation

Although *S. cerevisiae *can incorporate carbon from a number of different molecules, glucose is always used as the preferred carbon source [30]. When glucose is limiting, yeast undergoes a central reprogramming of its metabolism, with changes in the concentration of internal metabolites, stability of mRNAs and proteins, activity of enzymes, and the rate of transcription of a high number of genes. Genes encoding enzymes such as hexokinase (*HXK1*), glucokinase (*GLK1*) and glycerol kinase (*GUT1*), and transporters such as the high-affinity glucose transporters (*HXT6/7*) are amongst those that show the greatest degree of up-regulation (FDR < 1% for these and all the individual genes quoted below). This group also includes genes encoding transcriptional regulators themselves, such as *ADR1, CAT8, USV1*, and *MTH1*, demonstrating a role for transcriptional control in the signal transduction pathways regulating carbon metabolism in response to glucose availability.

At the proteome level, 21 proteins show a more than two-fold increase in abundance in carbon-limited conditions relative to the mean abundance across all conditions, including hexokinase (Hxk1p), the glyoxylate cycle enzymes malate synthase (Mls1p) and isocitrate lyase (Icl1p), succinate dehydrogenase subunits 1 to 3 (Sdh1/2/3p), aldehyde dehydrogenases 1 and 3 (Ald1/3p) and the glycogen-debranching enzyme (Gdb1p).

Growth of *S. cerevisiae *under carbon-limitation in the presence of glucose and small amounts of ethanol has previously been reported to induce the expression of the enzymes of the glyoxylate shunt [38], and their expression is also subject to glucose repression (that is, higher expression at low glucose concentrations) [39]. Recent studies by Regenberg and coworkers have also shown that these enzymes may appear transcriptionally up-regulated at low growth rates compared to high growth rates (>0.3 h^-1^) under carbon-limitation with higher glucose concentrations [40]. We do not see repression of the glyoxylate shunt enzymes at higher growth rates under carbon-limitation in our data, but the highest growth rate tested here (0.2 h^-1^) is considerably lower than the upper limit used by Regenberg *et al*, so this does not conflict with their observations.

The functions and pathways most significantly associated with increased expression and protein abundance in carbon-limitation are related to the different pattern of energy-yielding metabolism under carbon excess conditions (respirofermentative) compared to carbon-limited conditions (respiratory), though *glycogen metabolic process *is also found to be associated with up-regulation (*P *< 1 × 10^-6^). Transcription factors (TFs) associated with up-regulation include Hap2/3/4/5p (*P *< 2 × 10^-34^), Nrg1/2p (*P *< 1 × 10^-15^), Msn2/4p (*P *< 5 × 10^-25^), Adr1p (*P *< 1 × 10^-19^), Cat8p (*P *< 1 × 10^-11^), Gis1p (*P *< 1 × 10^-7^), Mig2p (*P *< 1 × 10^-6^) and Rsf2p (*P *< 1 × 10^-5^). All these TFs are known to have roles in mitochondrial gene expression and the stress response [41].

Genes that are down-regulated at the transcriptional level include those specifying the low-affinity glucose transporters Hxt1/3p and transcriptional regulators Gcr1p and Std1p. At the protein level, seven proteins show a more than two-fold decrease in abundance in carbon-limitation (all of which are also down-regulated at the transcriptional level); these include Hxt3p, alcohol dehydrogenase IV (Adh4p), DL-glycerol-3-phosphatase (Rhr2p) and D-lactate dehydrogenase (Dld3p).

In agreement with the GO slim analysis, terms associated with genes showing decreased expression in carbon limitation include *ribosome biogenesis and assembly *(*P *< 1 × 10^-15^), *nucleolus *(*P *< 1 × 10^-10^) and *rRNA metabolic process *(*P *< 1 × 10^-11^). Down-regulation of expression is also observed for genes regulated by transcription factors such as the zinc-regulated Zap1p (*P *< 1 × 10^-5^), the ribosome synthesis regulator Sfp1p (*P *< 1 × 10^-6^) and the glucose transporter regulator Rgt1p (*P *< 1 × 10^-7^). The significance of these findings will be discussed in more detail later.

#### Nitrogen limitation

Many studies on nitrogen regulation compared the response to the presence versus the absence of a nitrogen source, or the relative responses to different nitrogen sources. In our experiments, we study the role of nitrogen abundance by comparing ammonia limitation to ammonia excess conditions.

Although the size of the response is less extensive than that to glucose derepression, nitrogen limitation elicits a transcriptional response analogous to that of nitrogen catabolite repression (NCR) that involves the up-regulation of a number of pathways [42]. Genes that respond most strongly include the allantoin pathway genes *DAL1*/*2*/*4*/*5*/*7*/*80*; proline-utilization genes *PUT1*/*2*; genes for glutamate-metabolizing enzymes *GLT1*/*GDH1*; and those for amino-acid and ammonium transporters such as *GAP1*, *MEP2*, *VBA1*, and *AVT1*/*4*.

The main up-regulated functions under nitrogen-limitation appear to be related to the vacuole and cell cycle (*vacuole*: *P *< 1 × 10^-4^; *mitotic cell cycle*: *P *< 1 × 10^-4^), while up-regulated TF genes include the nitrogen degradation pathway regulator Dal80p (*P *< 1 × 10^-12^), and the NCR regulators Gln3p (*P *< 1 × 10^-13^) and Gat1p (*P *< 1 × 10^-7^)

Down-regulated genes include those encoding amino-acid transporters *GNP1*, *BAP2/3*, *AGP1 *and *TAT2*, and some enzymes involved in amino-acid metabolism such as *GLY1 *and *LYS1*. The only proteins showing a more than two-fold decrease in protein expression under nitrogen-limitation are those that are up-regulated in carbon-limitation relative to all other conditions and do not appear to be nitrogen-specific.

#### Phosphorus limitation

Similarly to nitrogen-limitation, phosphate-limitation induces a smaller expression response, in terms of the number of genes involved [43], than does carbon limitation. This is, perhaps, due to the small number of phosphate sources that *S. cerevisiae *can utilize relative to the large number of carbon sources it can assimilate. Those genes that are under phosphate control show a strong effect, however, including *PHO5*/*8*/*11*/*81*/*84*/*86*/*89 *and other genes involved in polyphosphate accumulation and metabolism, such as those for the vacuolar proteins Vtc2/3/4p. Vtc4p also has a more than two-fold higher protein abundance in this condition, along with three other proteins: the aspartic protease Yps1p, the purine-cytosine permease Fcy2p, and ribosomal protein Rpl15Ap.

GO analysis of significantly up-regulated genes in phosphate-limited cultures show *microautophagy *(*P *< 1 × 10^-10^) and phosphate-related terms (*phosphate transport*; *P *< 1 × 10^-6^) as the most overrepresented GO terms. KEGG pathways including significantly up-regulated genes include glycolysis (*P *< 1 × 10^-3^), which is also detected in the GO analysis. The gene encoding the phosphate-responsive TF, Pho4p, is strongly up-regulated (*P *< 1 × 10^-17^) along with TFs that have roles in DNA synthesis and repair such as Swi4p (*P *< 1 × 10^-10^).

In contrast to the up-regulation of glycolytic genes in phosphate-limitation, GO analysis of significantly down-regulated genes shows that respiration (*oxidative phosphorylation*: *P *< 1 × 10^-25^) and several genes encoding respiratory enzymes such as *COX5A*/*8*, *SDH1*/*2*/*4 *and the ubiquinol cytochrome C reductase *RIP1*(YEL024w) are down-regulated. As with nitrogen-limitation, the only proteins showing a large down-regulation response to phosphorus limitation are those up-regulated in carbon-limitation (that is, due to glucose derepression).

#### Sulphur limitation

Sulphur limitation evokes the smallest specific transcriptional response of the four conditions tested, and few sulphur-specific processes were identified as being under transcriptional control. That said, individual genes with roles in sulphur metabolism such as *SAM4 *(a controller of the methionine/S-adenosylmethionine ratio), *OAC1 *(encoding a mitochondrial sulphate transporter), *CYS3 *(involved in the trans-sulphuration pathway), and *MET22 *(involved in methionine biosynthesis) are found to be up-regulated in this condition. Three proteins had more than two-fold increases in protein abundance in the sulphur-limited samples: the RNA-metabolizing proteins, Rtc3p and Erb1p, and the alcohol dehydrogenase, Adh3p. None of the genes for these proteins show a significant change in mRNA levels in this condition, suggesting post-transcriptional regulation of these proteins in response to sulphur-limitation.

GO terms associated with up-regulated genes in sulphur limitation are ribosome and amino-acid related (*ribonucleoprotein complex biogenesis and assembly*; *P *< 1 × 10^-26^; *amino acid metabolic process*; *P *< 1 × 10^-9^) along with sulphur-specific terms such as *sulphur metabolic process *(*P *< 1 × 10^-4^) and *methionine biosynthetic process *(*P *< 0.001). KEGG pathways relating to amino-acid metabolism were also found to be up-regulated (*phenylalanine, tyrosine and tryptophan biosynthesis*: *P *< 1 × 10^-5^).

Few significant terms were found to be associated with genes down-regulated under sulphur limitation. However, a number of genes encoding proteins involved in the oxidative stress response that use glutathione or disulphide bonds in their mechanisms (*TSA2*, *GRX2*, *GRX6*) were amongst the most down-regulated. At the protein level, three significantly down-regulated (a more than two-fold decrease) proteins were detected: the isocitrate dehydrogenase Idp2p, the essential nucleolar protein Mak5p, and the RNA polymerase I subunit A14 (Rpa14p).

The correlations between transcriptome and proteome expression patterns and the results from Figure 2 show that changes at the transcriptome level are broadly transmitted to the proteome level. However, the correlation coefficients are relatively low, so we expect post-transcriptional control to play a significant role in the response to nutrient limitation. The extent of post-transcriptional control estimated in this way must be considered an underestimate, since many other mechanisms (for example, post-translational modifications) will need to be studied in order to get a complete picture. Previously, we showed that it is possible to quantify the relative changes in translational efficiency between two conditions, for example at two different growth rates, and defined the *translational control efficiency *(TCE) as the ratio of relative change in the level of a protein to the relative change in the level of its cognate mRNA between two states [7].

**Figure 3:**
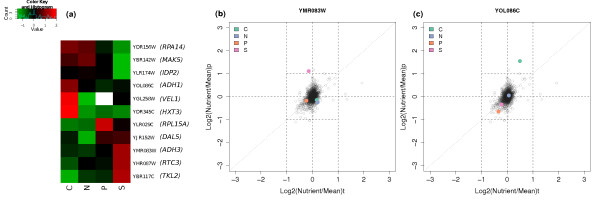
**Post-transcriptional control due to nutrient-limitation**. **a) **Individual genes that show significant changes in translational control efficiency between nutrients. Colours indicate the log. ratio of translational control efficiency in one nutrient compared to the other three. Only genes with a more than two-fold difference in translational efficiency in at least one condition relative to the overall mean, as well as more than two-fold changes in either protein or transcript levels, are shown. Proteins not detected in a given condition are shown in white. **b) **Protein and transcript log. fold changes in each nutrient-limiting condition relative to the overall mean for *ADH3 *(YMR083W). A random sample of fold changes from other genes is shown in grey for comparison. **c) **Protein and transcript log. fold changes for *ADH1 *(YOL086C).

Our data allow us to investigate nutritional effects on the TCE of each gene. In this case, we compare the change in mRNA level in a given nutrient limitation relative to the overall mean to the change in protein abundance in that condition relative to the overall mean. Figure 3(a) shows the 11 ORFs where the TCE is more than two in one or more conditions and more than two-fold changes are seen in either mRNA or protein abundance. These outliers in the proteome/transcriptome correlation plots may constitute important control steps for eukaryote cell growth, and hence are subjected to careful regulation.

A high TCE for a gene in a particular nutrient limitation relative to the others implies that some mechanism is boosting protein levels relative to mRNA levels in that condition. Conversely, a low TCE means some mechanism is reducing the protein level relative to the mRNA level. Plots of the transcript and protein level changes across the different conditions for all the ORFs shown in Figure 3(a) are given in Additional Files 15, 16, 17, 18, 19, 20, 21, 22, 23, 24, 25.

As expected, all three proteins found to be down-regulated at the protein level in sulphur-limitation also show a low TCE in this condition. In the case of Rpa14p (YDR156W) and Mak5p (YBR142W) there is no significant change in mRNA levels in any condition, so the regulation appears to be entirely post-transcriptional. For Idp2p (YLR174W), transcript and protein abundance both increase in carbon-limitation (but in proportionate amounts, so the TCE is unaffected); while in nitrogen, phosphorus- and sulphur-limitation, gene expression levels are similar. Uniquely in sulphur-limitation, however, we observe the protein abundance to be very low relative to the other conditions, suggesting that post-transcriptional regulation acts in this condition.

Three ORFs show a high TCE in sulphur-limitation: Adh3p (YMR083W), Rtc3p (YHR087W) and the transketolase Tkl2p (YBR117C). Again, for the first two, there are no large effects on transcript levels due to nutrient limitation, but protein abundance is more than two-fold higher in sulphur-limitation in both cases. The pattern of changes for Adh3p is shown in Figure 3(b). The pattern for Tkl2p is more complicated: the level of its mRNA is high in carbon-limitation relative to the other three conditions, but protein abundance is approximately the same in both carbon and sulphur-limitation, suggesting that some post-transcriptional regulation occurs in both these two conditions, lowering TCE in carbon-limited conditions and raising it in sulphur-limited conditions.

Three ORFs have a high TCE in carbon-limitation: Adh1p (YOL086C), Vel1p (YGL258W; a protein of unknown function), and Hxt3p (YDR345C; a high-affinity glucose transporter). Adh1p shows a small increase in its mRNA level in carbon-limited conditions, but a much higher increase in protein abundance leading to a high TCE (see Figure 3(c)). While Vel1p and Hxt3p have much lower mRNA levels in carbon-limitation relative to the other conditions (approximately 16-fold in the case of *HXT3*), but the much smaller reductions in protein abundance (less than four-fold for Hxt3p) suggest that some post-transcriptional mechanism prevents the full reduction in mRNA level from impacting on protein abundance.

**Figure 4:**
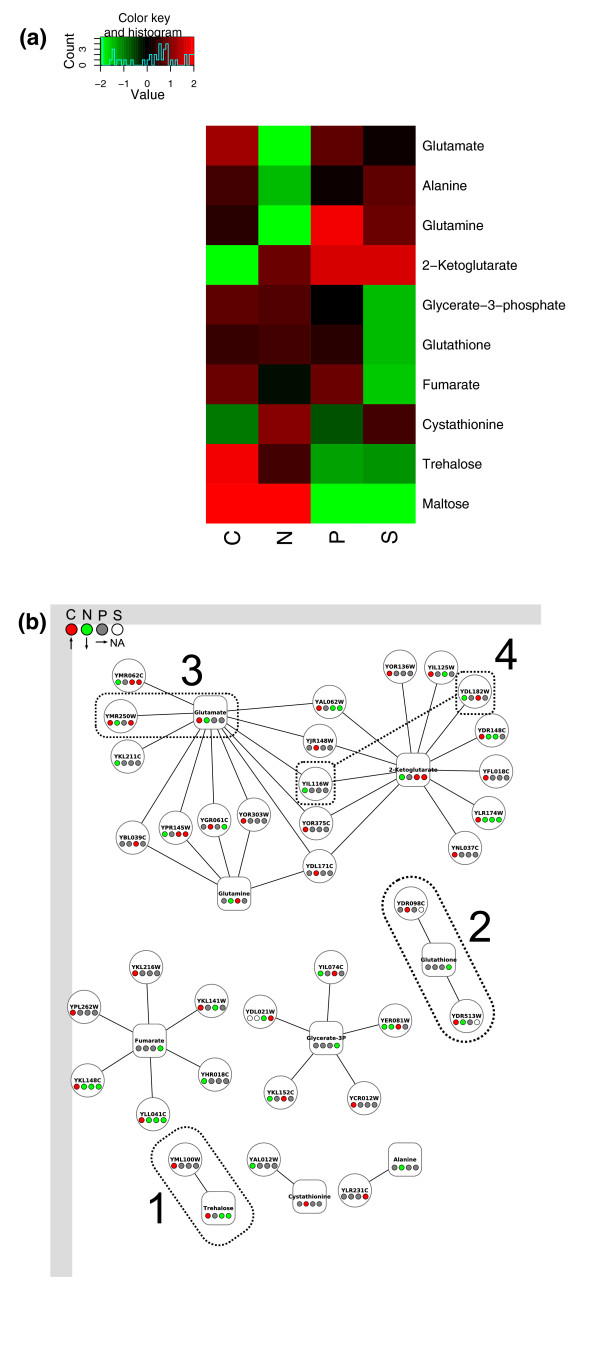
**Compounds that show significant changes in abundance between nutrients**. **(a)**. Colours indicate the log. ratio of the abundance in one nutrient compared to the overall mean. Only compounds with a more than two-fold change in abundance in at least one condition relative to the mean are shown. Other details are the same as Figure 1. The same compounds are shown in a simplified metabolic network in **(b) **connected to their cognate enzymes. Colours in each node represent the fold change in the measured metabolite (more than two-fold) or protein (>1.25-fold) abundances. Nodes referred to in the text are highlighted and numbered.

Finally in this section, we integrated data from our endometabolome analyses. Figure 4(a) shows those compounds that show a more than two-fold higher or lower intracellular abundance in one or more of the nutrient limitations relative to the overall mean. Nitrogen-limitation appears to have a strong effect on the levels of certain amino acids, with decreases in the levels of glutamate, glutamine and alanine visible in this condition. Surprisingly, however, levels of another amino acid, cystathionine, are elevated more than two-fold in nitrogen-limitation.

In carbon-limitation, we observe high levels of the carbon storage molecule trehalose, as well as another sugar, maltose. The responses to carbon limitation relating to carbon storage molecules such as trehalose (and glycogen whose levels we did not measure directly) are discussed further below. We also see significantly lower levels of the tricarboxylic acid (TCA) cycle intermediate 2-ketoglutarate in carbon-limited growth. Of the other TCA cycle intermediates measured, citrate also showed a decreased level (approximately 1.5-fold lower) while fumarate, malate and succinate levels were elevated (1.2- to 1.5-fold) in this condition, particularly relative to the phosphate and sulphur-limited conditions. Succinate for example was 1.9-fold lower in phosphorus-limitation, just below our cutoff.

The other three metabolites that showed a strong effect are glycerate-3-phosphate, fumarate, and glutathione, which are all observed at low levels in sulphur limitation. Glutathione's connection to sulphur metabolism is obvious, and glycerate-3-phosphate is consumed in the first step of the biosynthesis of the sulphur-containing amino acids cysteine and methionine. However, the connection of fumarate to sulphur metabolism is not clear.

Figure 4(b) shows these metabolites connected to those enzymes which either consume or produce them in a recently produced consensus model of the *S. cerevisiae *metabolic network [44] and whose protein levels vary by >1.25-fold in one or more nutrient-limitations relative to the overall mean. In most cases we find little or no correlation between the level of the enzymes and the metabolites, suggesting that metabolite levels are controlled by the system-level properties of the metabolic network, rather than by individual enzymes. Having said this, a number of observations can be made: 1) trehalose levels increase in carbon-limitation as does the abundance of Tls1p (YML100W) a subunit of the trehalose-synthesising enzyme trehalose 6-phosphate synthase. 2) Glutathione levels fall in sulphur-limitation, under which conditions two glutathione-dependent oxidoreductases Grx2p (YDR098C) and Grx3p (YDR513W) are undetectable. 3) Glutamate levels are raised in carbon-limitation and reduced in nitrogen-limitation, a pattern matched by the glutamate-catabolising enzyme, Gad1p (YMR250W). 4) Levels of 2-ketoglutarate fall in carbon-limitation, a pattern that is reversed in almost all its consuming/producing enzymes, most of which are mitochondrial and whose protein levels rise in carbon-limitation. Exceptions to this rule are His5p (YIL116W) and Lys20p (YDL182W), which are involved in amino-acid biosynthetic pathways and whose levels fall in this condition.

### Nutrient- and growth-rate dependent regulation at the transcriptome, proteome and metabolome levels

Having identified systems regulated by nutrient availability, next we looked for those regulated by growth rate in each condition. As shown in Figure 1, for the transcript data a linear regression of expression levels against growth was made for each gene in each nutrient limitation in turn, and the slope of the regression was determined. Table 2 shows the number of genes with significant non-zero slopes in each condition. Although, as we saw above, carbon-limitation elicits a large change in the expression levels of many genes, the number of genes that are under growth-rate regulation appears to be low in this condition. Phosphorus-limitation, in contrast, has the most growth-rate-regulated genes.

**Table 2:** Number of genes significantly regulated with increasing growth rate in each nutrient-limiting conditions (FDR < 5%).

	Carbon	Nitrogen	Phosphorus	Sulphur
**Up regulated**	801	1,199	1,552	1,091
**Down regulated**	588	1,158	1,783	1,124
**Total**	1,389	2,357	3,335	2,215

**Figure 5:**
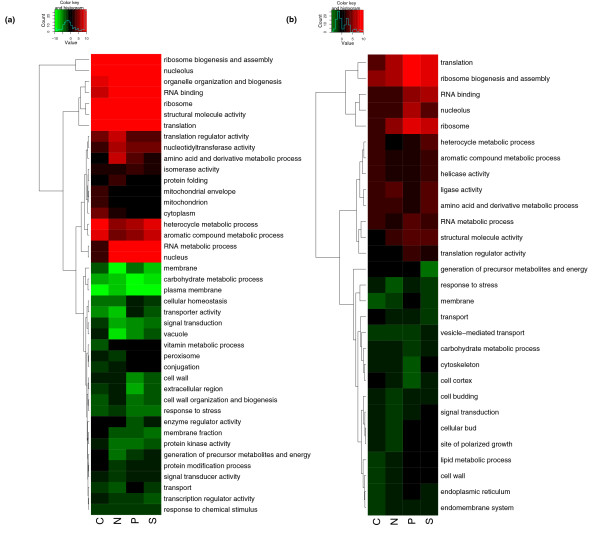
**GO_slim analysis of functional categories associated with growth rate related changes in mRNA (a) and protein levels (b)**. Colours indicate the significance of the association of the given GO term with up- (red) or down- (green) regulation with increasing growth rate in a given nutrient limitation. Other details are the same as for Figure 1.

Figure 5(a) shows, for each of the four nutrient-limiting conditions, the association of each GO slim term with up- or down-regulation of mRNAs with increasing growth rate. As expected from previous studies [7,8], ribosomal terms show a clear up-regulation with increasing growth rate in all four conditions. In contrast, membrane, cell wall and stress-related terms tend to be associated with genes that are down-regulated with growth rate in all four conditions. Analysis of the enrichment of TF target gene sets with growth-rate regulated genes agreed with previous studies [8] in finding a number of TFs associated with either up- (Sfp1p, Fhl1p, Rap1p) or down-regulation (Pdr1p) of their target gene expression levels.

For the proteome, we measured the association of GO slim terms with positive (protein-level increases with increasing growth rate) or negative (protein-level decreases with increasing growth rate) changes, when comparing the two highest growth rates. This is shown in Figure 5(b). Broadly, the same effects as those seen at the transcript level are observed, with ribosome-related terms strongly up-regulated across all conditions and proteins with stress, membrane and cell-wall terms down-regulated. Results of the full GO, Kegg and Yeastract analyses of these parameters are given in Additional Files 26, 27, 28, 29, 30, 31, 32, 33, 34, 35.

Of the GO_slim terms associated with significant changes in expression with growth rate, none appears to show a qualitative difference between two different conditions (that is, increasing with growth rate in one while decreasing in another). Even when the same analysis is applied to all GO terms, only four unique terms are found where a significant up-regulation of expression is observed with increasing growth rate in one nutrient-limitation while a significant down-regulation of expression is observed in another. The four terms are: phosphate transport, vitamin B6 metabolic process, oxidative phosphorylation and carbohydrate binding.

**Figure 6:**
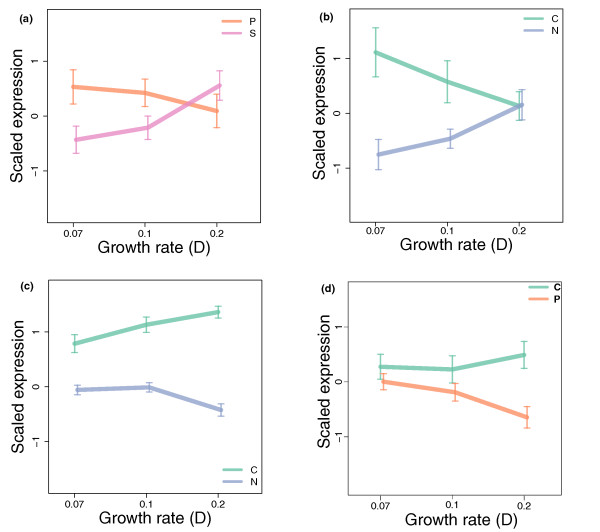
**Expression profiles of the genes for each of the four GO terms showing significant differences in growth rate responses in different conditions**: **a) **phosphate transport, **b) **vitamin B6 metabolic process, **c) **oxidative phosphorylation and **d) **carbohydrate transport. The expression values are scaled such that they have mean 0 and standard deviation 1. The error bars represent one standard error. For clarity, plotting positions on the x-axis are shifted slightly for each condition.

Genes concerned with phosphate transport are down-regulated with increasing growth rate in phosphate-limited cells and up-regulated in cells growing under the other nutrient conditions. The expression profile for these genes (see Figure 6(a)) shows that their transcript levels are elevated at low growth rates in phosphate-limited cells as compared to sulphate-limited ones, but that this difference diminishes as growth rate increases until, at the highest growth rate used in this study (0.2 h^-1^), the mRNA levels of these genes is approximately the same in the two conditions. In fact, as we discuss further in the Conclusion, this effect is quite general and similar effects are observed under nitrogen- and sulphur-limitation, although not in carbon-limited conditions.

Of the 11 genes annotated with the *vitamin B6 metabolic process *GO term, only three show a high degree of congruence with the pattern shown in Figure 6(b): *SNO1*, *SNZ1*, and *SNZ2 *(all members of a family of genes whose expression is induced in stationary phase, but whose functions are poorly defined) [45]. *SNO1 *and *SNZ1 *show the strongest effect and are believed to form a glutamine amidotransferase complex [46,47]. Given the pattern of expression in carbon and nitrogen limitation, and glutamine's role in the metabolism of both nutrients [42] this is an interesting observation. However, further biochemical characterization of the function of this enzyme would be required to make any firm hypothesis concerning its role.

As one might expect, given the importance of respiration in carbon-limiting conditions, genes related to oxidative phosphorylation show an increase in expression with increasing growth rate in carbon limitation (Figure 6(c)). However, our analysis shows that, particularly at the highest growth rate (D = 0.2 h^-1^), expression of these genes tends to decrease in nitrogen-limited cells. Similarly, genes annotated with the *carbohydrate binding *term show a small increase in expression with increasing growth rate in carbon limitation, while decreasing in expression in phosphate limitation (Figure 6(d)).

Two proteins show more than two-fold changes of opposite signs in their protein levels in two different nutrient-limited conditions: Ser3p and Erg1p. Ser3p is the 3-phosphoglycerate dehydrogenase that uses the glycolytic intermediate 3-phosphoglycerate to catalyse the first step in the serine and glycine biosynthesis pathway that is active during growth on glucose [48] (the alternative pathway uses glyoxylate as the precursor). Compared to growth at D = 0.1 h^-1^, Ser3p shows a more than two-fold decrease in abundance at D = 0.2 h^-1 ^in phosphate limitation and a more than two-fold *increase *under carbon limitation. Erg1p, a squalene epoxidase that catalyses a key step in ergosterol biosynthesis [49], shows the opposite trend to Ser3p, increasing more than two-fold in abundance in phosphate limitation while decreasing more than two-fold in carbon limitation.

**Figure 7:**
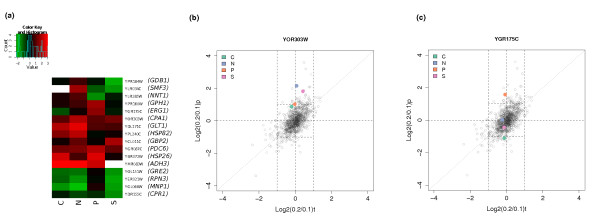
**Nutrient-limitation specific post-transcriptional control due to growth rate changes**. **a) **Individual genes that show significant changes in translational control efficiency between growth rates in each nutrient limitation. Colours indicate the log. ratio of translational control efficiency between the two growth rates. Only genes with a more than three-fold change in translational efficiency are shown. Genes not detected in a given condition are shown in white. Other details are the same as Figure 3(a). **b) **Protein and transcript log. fold changes for the shift from D = 0.1 h^-1 ^to D = 0.2 h^-1 ^in each nutrient-limiting condition for *CPA1 *(YOR303W). A random sample of fold changes from other genes is shown in grey for comparison. **c) **Protein and transcript log. fold changes for *ERG1 *(YGR175C).

We can identify growth-rate related control at the post-transcriptional level by looking for changes in TCE between growth at D = 0.2 h^-1 ^and D = 0.1 h^-1 ^for each condition (ORFs with more than three-fold changes are shown in Figure 7(a)), rather than between nutrient-limitations as was done earlier. The ORFs with more than two-fold TCE changes and plots of the transcript and protein level changes across growth rates in each of the different conditions for the ORFs shown in Figure 7(a) are given in Additional Files 36, 37, 38, 39, 40, 41, 42, 43, 44, 45, 46, 47, 48, 49, 50, 51, 52.

As described previously [7], some ORFs, such as Cpa1p (YOR303W), which is known to be regulated post-transcriptionally [50], show a positive TCE with increasing growth rate in all four conditions (Figure 7(b)). Others in this category include the nicotinamide adenine dinucleotide (NAD)-dependent glutamate synthase Glt1p (YDL171C) and the mitochondrial alcohol dehydrogenase Adh3p (YMR083W) both of which show small increases in gene expression but much larger increases in protein abundance when growth rate is increased. The growth-rate-linked post-transcriptional up-regulation of Adh3p may be responsible for the increased ethanol production observed in all conditions at the highest growth-rate (see Additional File 53). Also, high levels of Adh3p are necessary for the oxidation of surplus mitochondrial NADH, produced via the increased biosynthesis of amino acids such as leucine at the higher growth rate. The actions of Adh1p and Adh3p then act as a redox shunt providing a flux of NADH from the mitochondria to the cytosol [51].

Other ORFs show nutrient-specific effects. For instance, the ergosterol biosynthesis enzyme Erg1p (YGR175C) shows an increased TCE with increasing growth rate under phosphorus limitation and a decreased TCE under carbon limitation (Figure 7(c)). Ergosterol is a major constituent of the plasma membrane and Erg1p, as a specific target of anti-fungal allylamine drugs such as terbinafine, has been well studied [52]. Another enzyme in the pathway, Erg11p, has been reported as potentially being under post-transcriptional regulation in *Candida glabrata *[53], but this is the first report of post-transcriptional regulation for *ERG1 *in *S. cerevisiae*.

**Figure 8:**
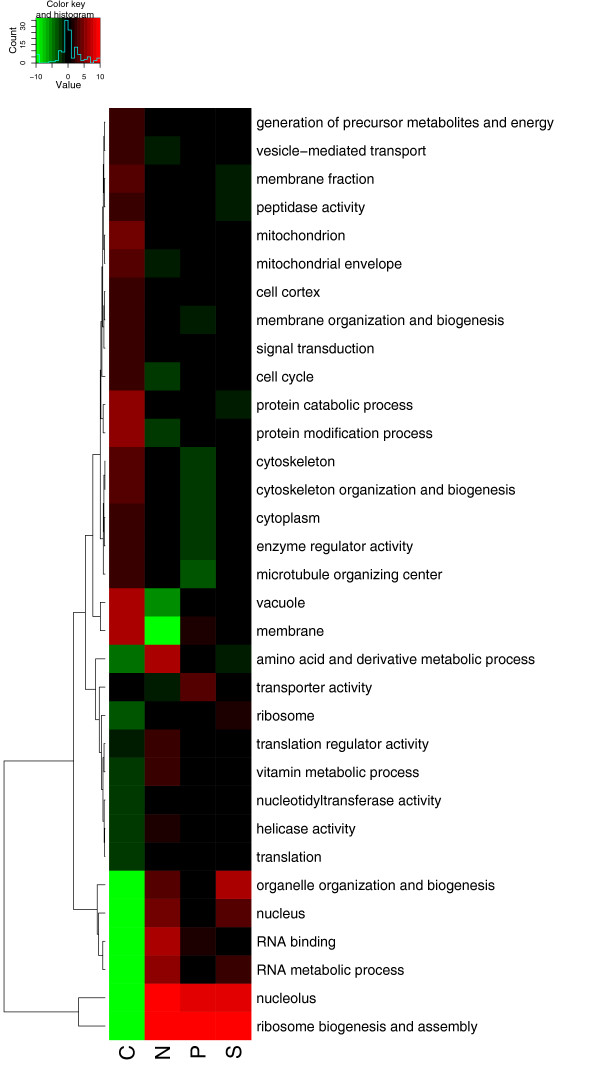
**GO_slim analysis of functional categories associated with differences in the gene expression response to growth rate in different nutrient-limiting conditions**. Colours indicate the significance of the association of the given GO term with relative up- (red) or down- (green) regulation with increasing growth rate in a given nutrient-limitation compared to the overall response across all limitations. Other details are the same as Figure 1.

From the above analyses, we can see that qualitative differences in regulation by growth-rate between different nutrient environments are quite rare. However, significant quantitative variations in the degree of growth-rate regulation between different conditions are more common, as we show below. To identify genes showing these differences, we used ANCOVA on the transcript data to compare the slope of the relationship between gene expression and growth rate obtained from one condition with the overall slope obtained across all four conditions. Figure 8 shows the association of GO slim terms with these *differences *between slopes.

The result from this analysis is that, although genes with ribosome-related terms are strongly up-regulated with growth rate in all conditions (see Figure 5(a)), there is a significantly weaker up-regulation in the carbon-limited case (*P *< 1 × 10^-71^). In contrast, genes associated with glycogen and trehalose metabolism, whose expression tends to fall with increasing growth rate, have a much weaker fall in carbon limitation (*P *< 1 × 10^-10^). We describe the expression patterns observed for these two processes below using proteome data where appropriate.

Nutrient specific growth-rate effects are observed in other conditions as well. In nitrogen limitation, genes associated with the vacuole, which tend to be down-regulated with increasing growth rate, show a greater degree of down-regulation (*P *< 1 × 10^-6^) while genes involved in amino-acid biosynthesis, particularly of branched-chain amino acids, show a greater degree of up-regulation in this condition (*P *< 1 × 10^-5^). As expected, in phosphate limitation, the slope of regression for phosphate transport genes is significantly lower than the mean (*P *< 1 × 10^-5^) and genes involved in microautophagy and the starvation response show a similar effect (*P *< 0.001). Full GO, Kegg and Yeastract analyses are given in Additional Files 54, 55, 56, 57, 58, 59, 60.

#### Ribosomal protein production

The transcription of genes encoding ribosomal proteins is highly regulated by both growth rate and nutrient availability. This regulation is crucial to cell growth because ribosome biogenesis accounts for >50% of total transcription in eukaryotic cells [54]. The primary regulator of the transcription of ribosomal protein genes is the TF Sfp1p [55,56], which we find (in agreement with previous studies [8]) to be strongly associated with the expression of growth-rate up-regulated genes.

**Figure 9:**
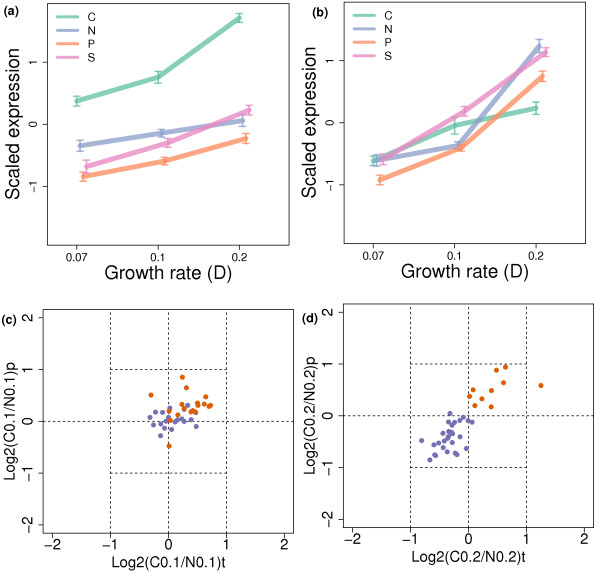
**Average patterns of expression of genes with a) *mitochondrial ribosome *and b) *ribosomal large subunit biogenesis and assembly *GO terms**. The expression values of each gene are scaled to mean 0 and standard deviation 1. Average scaled expression values are shown with error bars corresponding to one standard error. For clarity, plotting positions on the x-axis are shifted slightly for each condition. Scatter plots show the log. ratio of the transcript level in carbon-limitation to the level in nitrogen-limitation against the log. ratio of their respective protein levels for genes annotated with the *ribosomal large subunit biogenesis *GO term at **c) **D = 0.1 h^-1 ^and **d) **D = 0.2 h^-1^. These points are shown in blue. For comparison, mitochondrial ribosome genes are shown in orange.

Figure 9 shows the mRNA and cognate protein abundance profiles of the genes and proteins associated with two GO terms related to ribosome synthesis. We can see from this that, at the D = 0.1 h^-1 ^to D = 0.2 h^-1 ^shift, the change in expression of *mitochondrial ribosome *associated genes (Figure 9(a)) is significantly greater under conditions of carbon limitation than in carbon sufficiency (Wilcoxon *P *< 0.05), while the change in expression of *ribosomal large subunit biogenesis and assembly *associated genes is significantly smaller (Wilcoxon *P *< 1 × 10^-7^) (Figure 9(b)). Our proteome data confirm that this effect is transmitted to the protein level. Figures 9(c), (d) show plots of the ratio of protein levels of ribosome-biogenesis-related ORFs and mitochondrial ribosome ORFs in nitrogen limitation as compared to carbon limitation. The log. carbon to nitrogen ratios for transcript and protein levels both become significantly negative (Wilcoxon *P *< 1 × 10^-7^) at D = 0.2 h^-1^, having been no different from 0 at D = 0.1 h^-1^. This confirms that the relative levels of cytoribosomal proteins are lower in carbon-limitation compared to nitrogen-limitation, but only at the highest growth rate. One hypothesis for this effect is that, as the cell invests more and more resources and energy into mitochondrial biogenesis and mitochondrial protein synthesis in order to maintain respiration, cytoplasmic protein synthesis is sacrificed. The high cost of mitochondrial biogenesis may explain why a facultative organism, like yeast, favours fermentation over respiration despite the fact that it is less energy efficient [57].

#### Carbohydrate storage

**Figure 10:**
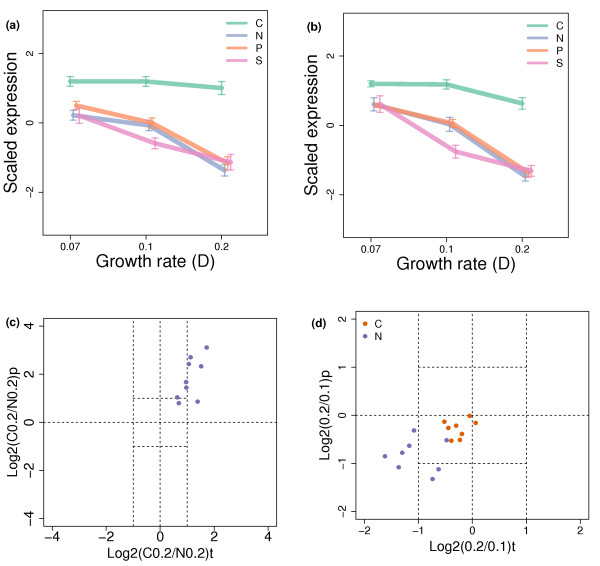
**Average patterns of expression of genes involved in glycogen and trehalose a) biosynthesis, and b) degradation**. The expression values of each gene are scaled to mean 0 and standard deviation 1. Average scaled expression values are shown with error bars corresponding to one standard error. For clarity, plotting positions on the x-axis are shifted slightly for each condition. **c) **Log. ratio of protein levels in carbon-limited culture to levels in nitrogen-limited culture versus the log. ratio of transcript levels for eight carbohydrate storage genes at D = 0.2 h^-1^. **d) **Log. ratio of protein levels at D = 0.1 h^-1 ^to protein levels at D = 0.2 h^-1 ^against the log. ratio of transcript levels for nitrogen-limited cultures (blue) and carbon-limited cultures (orange) for the same genes.

Carbohydrate storage-related GO terms show a strong nutrient-specific growth rate response in carbon-limitation. The genes annotated with these terms are associated both with significantly higher expression in carbon limitation relative to non-carbon limitation (*P *< 1 × 10^-5^), and a weaker fall in expression with increasing growth rate (*P *< 1 × 10^-10^). To better understand this effect, we selected all 12 genes from the Saccharomyces Genome Database (SGD) biochemical pathways *glycogen biosynthesis, glycogen catabolism, trehalose biosynthesis *and *trehalose degradation *and the essential biosynthetic enzyme UDP-glucose pyrophosphorylase. From these pathways, a natural division can be made into glycogen and trehalose biosynthetic and catabolic enzymes. Figure 10(a), (b) show the expression patterns for these two groups of genes. The up-regulation of these genes in carbon limitation at all growth rates is clear. It can also be seen that the drop in expression in carbon limitation with increased growth rate is significantly less than in non-carbon limitation. The only gene not conforming to this pattern is that encoding the sporulation-specific glucoamylase (*SGA1*) where expression drops a similar amount in all four conditions. *SGA1 *is induced to mediate glycogen catabolism in diploid cells during late sporulation, but is not thought to play a significant role in glycogen metabolism during vegetative growth [58,59].

Proteome data were available for 8 of the 12 enzymes. To assess whether the transcriptional effect was transmitted to protein levels, we calculated the ratio of the protein levels in carbon-limitation to the level in nitrogen-limitation at D = 0.2 h^-1^. Figure 10(c) shows the distribution of these ratios plotted against the equivalent transcript level ratio. The means of both the transcript and protein log. ratios are significantly greater than 0 (Wilcoxon test; *P *< 0.01), confirming that both mRNA and protein levels are significantly higher in carbon limitation at this growth rate. Figure 10(d) shows the ratios of the protein levels at D = 0.2 h^-1 ^to D = 0.1 h^-1 ^for both carbon and nitrogen limitation alongside the equivalent transcript ratios. In this case, the mean ratios for carbon and nitrogen limitations are both below 0, indicating that mRNA and protein levels fall when growth rate increases in both conditions. However, in the nitrogen-limited culture, the mean ratio is significantly lower than in carbon-limitation (Wilcoxon test; *P *< 0.01), confirming that the fall in protein levels is greater in nitrogen limitation.

Glycogen storage and release are known to be tightly coupled to nutrient availability, growth and the cell cycle, and the rate of glycogen deposition has been observed to be inversely proportional to growth rate [60-62]. However, this is the first time that this particular nutrient-specific growth-rate effect on the levels of glycogen-metabolizing enzymes has been observed. The transcriptional effect is most likely due to the activation of the transcriptional activators Msn2/4 binding to stress-responsive elements (STREs) [63]. The activities of Msn2/4p are themselves under the control of both TOR and the cAMP-PKA pathways [64,65], which are both tightly linked to growth control and nutrient sensing [66-69].

We note from our metabolome data, that trehalose, at least, is found at a higher abundance in the carbon-limited case (glycogen was not measured). However, the difficulty with making further conclusions from transcript and even protein-level data on this pathway is that the activity of glycogen synthase is regulated post-translationally by allosteric binding of glucose-6-phosphate and a series of phosphorylation/dephosphorylation processes under the control of the cAMP-PKA pathway [58]. In carbon limitation, we would expect low levels of the allosteric activator and high activity levels of the cAMP-PKA pathway, leading to low activity of this enzyme and low levels of glycogen deposition despite the high level of expression of these enzymes.

## Conclusions

It is important to understand that the complete biological response of the yeast cell (that is, all the genes and biological processes controlling cell growth under a specific nutrient-limiting condition) will always entail the integration of the nutrient-specific sensing, signal transduction, gene expression and metabolic networks together with the *core *of biological networks responsible for central cell growth.

In the course of this analysis, we have shown that the mRNA and protein levels of many *S. cerevisiae *genes are under the control of a combination of these nutrient-specific sensing mechanisms and growth rate. Many of these effects are well known from previous studies, but many unexpected interactions between nutrient availability, growth rate, and regulation have also been observed.

As expected from previous studies [10], of the four nutrients tested, carbon availability has the largest effect on the transcriptional pattern observed. This is due to the release of the effect of glucose repression on many genes [30,41], as indicated by the enrichment, in the up-regulated set, of those genes regulated by TFs such as Hap2/3/4/5 and Nrg1/2 [4], and the activation of respiratory metabolism, as indicated by large increases in the expression of genes related to mitochondrial functions. Less expected was the interaction between carbon limitation and growth rate that leads to changes in the expression of both the mitochondrial and respiratory genes (under carbon-limitation they increase their expression with increasing growth rate; see Figure 5(a)), as well as genes relating to synthesis and degradation of carbohydrate storage molecules (whose expression falls much less with increasing growth rate in carbon-limitation compared to other conditions; see Figure 10) and ribosomal biogenesis, whose expression increases less in carbon limitation at the highest growth rate shift (see Figures 8 and 9), perhaps due to the concentration of cellular resources on mitochondrial biogenesis. Carbon limitation is also used as a signal by the post-transcriptional regulatory machinery. The alcohol dehydrogenase gene *ADH1*, for instance, shows a more than two-fold increase in the abundance of its protein product in carbon-limitation while showing only a small increase in its mRNA level, suggesting post-transcriptional regulatory mechanisms are responding to nutrient availability in this case.

**Figure 11:**
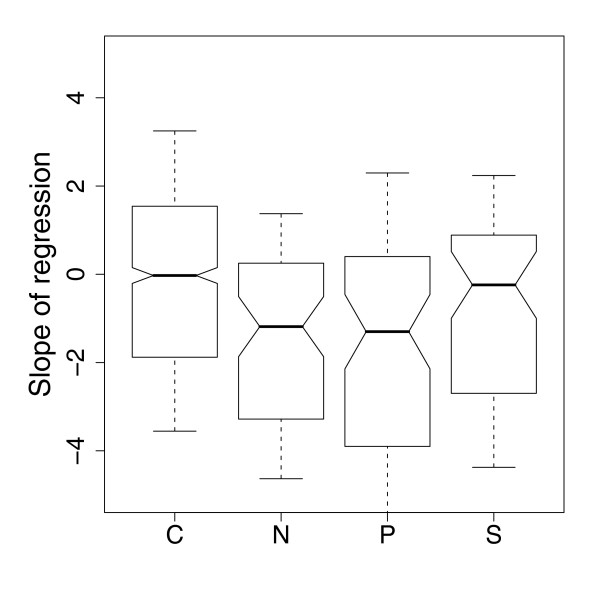
**Distribution of the regression slopes fit to expression data of genes significantly up regulated in the nutrient limitation indicated (FDR < 5%)**. Negative slopes mean the gene is down-regulated with increasing growth rate. Bold lines represent the median slope for that nutrient; boxes represent the inter-quartile range; boxes whose notches do not overlap have average slopes that are significantly different from each other (*P *< 0.05).

The nitrogen-, phosphorus- and sulphur-limitations induce changes in a relatively small subset of genes compared to carbon, and these changes tend to have specific roles in the metabolism of those nutrients. Examples include the genes under the control of the Dal80p TF that are known to respond to the availability of nitrogen sources [70] and the *PHO *system that responds to phosphate levels [43]. Sulphur-specific pathways were not found, but individual genes with roles in the production of sulphur-containing amino acids, such as *CYS3 *and *MET22*, were detected [71]. The expression responses of these pathways can be generalized to a pattern whereby they are up-regulated in the limitation in question, but only at low growth rates. At high growth rates, the up-regulation of these genes is reduced and often removed altogether (see the phosphate transporter in Figure 6(a)). This is in contrast to the effect of growth rate on the respiratory genes up-regulated in carbon limitation, which tends to not only increase in expression as growth rate increases, but increase faster in carbon limitation (see Figures 5 and 8). To confirm the generality of this observation, Figure 11 shows the average slope of the linear regression of expression against growth rate for those genes up-regulated in each nutrient limitation (FDR < 5%). Genes up-regulated in nitrogen, phosphorus and sulphur limitation all tend to have slopes < 0 (Wilcoxon test *P *< 0.01), indicating that the expression of these genes falls with increasing growth rate, while the mean slope of genes up-regulated in carbon limitation is not significantly different from 0. This observation may reflect the unique role of glucose as both a biosynthetic material and an energy source, while the other nutrients are purely biosynthetic.

At the growth rate level, ribosome synthesis is the dominant process in all conditions - in keeping with its position as the single most powerful consumer of both the energy and the biosynthetic resources required for growth [54]. This process is heavily regulated at the transcriptional level, principally by the transcriptional activator Sfp1p [55,56], which is downstream to, and a feedback regulator of, the central growth-rate-regulating kinase TOR [72]. It is not surprising, therefore, that we find genes related to ribosomal synthesis transcriptionally regulated by a complex mix of nutrient and growth-rate effects, with carbon, nitrogen and sulphur limitation all showing similar but distinct patterns of expression (see Figure 9). With our proteome data, we are able to confirm that at least some of these effects, including a significantly lower rate of synthesis of cytosolic ribosome levels at the highest growth rate under carbon limitation (perhaps due to the very high demand for mitochondrial ribosomes), are subsequently transmitted to protein levels.

The integration of proteome and transcriptome data also gives an important insight into post-transcriptional regulation due to both nutrient and growth-rate effects. While Adh1p, for example, shows evidence of nutrient mediated post-transcriptional regulation, another alcohol dehydrogenase (Adh3p) shows strong growth rate mediated regulation. The detection of differential post-transcriptional regulation between carbon and phosphate-limited conditions for a gene involved in ergosterol biosynthesis (*ERG1*) suggests that this pathway is regulated both by nutrient-sensing and growth-rate. Another biosynthetic enzyme, Ser3p, also appears to be differentially regulated by different nutrients in response to growth rate. Ser3p is part of a biosynthetic pathway leading to serine that is only active during growth on glucose [48,73]. The increase in Ser3p levels with increasing growth rate in carbon-limitation is interesting therefore, especially when contrasted with the situation in phosphate-limited growth, where Ser3p levels fall with increasing growth rate.

With regard to future work, although we are beginning to form a picture of the processes under nutrient and growth-rate control, the static snapshots that these measurements represent cannot truly reflect the inherently dynamic nature of metabolism and cell growth. A better understanding of these processes will require the integration of these data with dynamic measurements of pathway fluxes [74]. This will be necessary for both metabolic and signaling pathways, since the activity of many pathways is not determined by the levels of their protein components, but by the activity of those components, which are often controlled at a post-transcriptional (and, sometimes, purely metabolic) level. A more complete perspective would also require the integration of high-throughput data with information from molecular studies and screenings with mutants.

In conclusion, we have presented an integrated view of the transcriptional, translational, and metabolic responses of a eukaryotic cell to nutrient supply and growth rate. We show that each of the nutrients investigated induces a specific response at the transcriptional and post-transcriptional level and alters the response of many genes to changes in growth rate. We also show that carbon induces both a unique response and a unique pattern of response, possibly due to its role as both an energy source and a biosynthetic compound. Our integrated analysis also allows us to identify many novel examples of nutrient- and growth-rate-regulated post-transcriptional controls.

## Methods

### Yeast strain, media used and transcriptome/proteome/metabolome sampling

The diploid *Saccharomyces cerevisiae *strain FY1679 (*MAT**a***/*MATα ura3-52*/*ura3-52 leu2-1*/+ *trp1-63*/+ *his3-D200*/+ *ho*::*kan*MX4/*ho*::*kan*MX4) was used for all the experiments. Conditions for chemostat cultivation in a mineral medium under C, N, P and S nutrient limitation have been described previously [7]. Measurements of transcript, protein, and metabolite levels were made and then processed as described previously.

Proteome data from [7] were reanalyzed using updated software solutions. The MS/MS spectra were searched against an updated version of the *S. cerevisiae *protein database (current as of February 2010) using Mascot, and the peptide-spectrum matches resulting from this search were processed by Mascot Percolator [67] to calculate false discovery rate (q-value) scores. The peptide-spectrum matches were then paired with the iTRAQ reporter ion tag data using iSPY (manuscript in preparation), an updated software expanding the capabilities of iTRACKER [68]. An FDR threshold of 1% (q </= 0.01) was applied at the peptide level. After this, the individual peptide levels were log. transformed and MAD (mean absolute deviation) normalized. Protein abundances were then calculated by averaging the abundance of all detected peptides.

After this, the individual peptide levels were log. transformed and MAD (mean absolute deviation) normalized. Protein abundances were then calculated by averaging the abundance of all detected peptides.

### Detection of differential expression, protein levels and translational control efficiencies

For each gene, first, the overall mean mRNA level was calculated and a linear regression was performed of transcript level against specific growth rate, employing data from all four conditions and using the maximum likelihood estimation implementation in R [75]. To detect nutrient effects, the mean transcript level from a given condition was compared to the overall mean using Student's t-test. To detect genes responding to growth rate, separate linear regressions using maximum likelihood estimation were made for each condition and the significance of the difference of the slope from zero calculated. To detect nutrient-specific growth-rate responses, an ANCOVA model was used to determine the significance of the difference in slopes between the overall regression and the regression for each individual condition. A graphical representation of the different parameters calculated is given in Figure 1.

In all cases, false discovery rates (FDRs) were derived using the method of Benjamini and Hochberg [35] and the values of the appropriate statistic (t statistic or slope) were used for functional analysis.

Processed protein and metabolite levels were compared by calculating log. fold ratios of the measured abundance between different limiting nutrient or growth rate conditions as appropriate. The log. fold ratios were used for functional analysis. Proteome-transcriptome correlations and relative changes in translation efficiencies were calculated as described previously  [7,76].

### Functional analysis and GO term filtering

Functional analysis was performed using a method similar to LRpath [34]. Gene annotations were downloaded from GO [33], Kegg [36], and Yeastract [37]. For each functional classification (GO term, Kegg pathway or TF), the list of all genes detected in the experiment (transcriptome or proteome) was encoded as a vector (the function vector) of 1's (meaning the gene is annotated with the function in question) and 0's (meaning the gene is not annotated with the function in question). A second vector was then generated from the given statistic (t-statistic or slope for transcriptome studies, log. fold change for proteome) and a logistic regression performed of this vector against the function vector using maximum likelihood estimation.

The slope parameter of the regression then corresponds to the change in the log. odds of a gene belonging to the specific category (GO term, Kegg pathway, or TF) for a unit increase in the given statistic. Additional File 61 shows the use of the approach for the ribosome biogenesis GO term and its relationship to the slope of the linear regression.

*Heat maps *showing the results of the analyses were made using the heatmap.2 function from the gplots package of R. All other methods were implemented in either Ruby using the BioRuby package [77], RSRuby [78], or R [75] using packages from Bioconductor [79].

## Abbreviations

ANCOVA: Analysis of covariance; cAMP: Cyclic adenosine monophosphate; FDR: False discovery rate; GC/TOF-MS: Gas chromatography/time-of-flight mass spectrometry; GO: Gene ontology; iTRAQ: isobaric tag for relative and absolute quantitation; ORF: Open reading frame; MAD: Median absolute deviation; MS: Mass spectrometry; NAD: Nicotinamide adenine dinucleotide; NCR: Nitrogen catabolite repression; RNA: Ribonucleic acid; SAM: S-adenosyl methionine; SGD: Saccharomyces genome database; STRE: Stress responsive element; TCA: Tricarboxylic acid; TCE: Translational control efficiency; TF: Transcription factor.

## Competing interests

The authors declare that they have no competing interests.

## Authors' contributions

AG and PP carried out the statistical analyses. JIC, SGO, AG and PP interpreted results. PC and KL performed the proteomics MS/MS spectra analyses. AG, PP, JIC and SGO drafted the manuscript. SGO conceived of the study, and both SGO and KSL participated in its design and coordination. All authors read and approved the final manuscript.

## Supplementary Material

Additional file 1 **Proteome/transcriptome correlation (carbon)**. Log. fold changes on increasing growth rate from D = 0.1 h^-1 ^to D = 0.2 h^-1 ^for protein (p) and gene expression (t) levels in carbon limitation. Click here for file

Additional file 2 **Proteome/transcriptome correlation (nitrogen)**. Log. fold changes on increasing growth rate from D = 0.1 h^-1 ^to D = 0.2 h^-1 ^for protein (p) and gene expression (t) levels in nitrogen limitation. Click here for file

Additional file 3 **Proteome/transcriptome correlation (phosphorus)**. Log. fold changes on increasing growth rate from D = 0.1 h^-1 ^to D = 0.2 h^-1 ^for protein (p) and gene expression (t) levels in phosphorus limitation. Click here for file

Additional file 4 **Proteome/transcriptome correlation (sulphur)**. Log. fold changes on increasing growth rate from D = 0.1 h^-1 ^to D = 0.2 h^-1 ^for protein (p) and gene expression (t) levels in sulphur limitation. Click here for file

Additional file 5 **GO, KEGG, Yeastract transcriptome/proteome analysis (carbon)**. Logistic regression results for carbon up/down regulated genes/proteins from the transcriptome (trans) and proteome (prot) data using GO, KEGG and Yeastract annotations. Click here for file

Additional file 6 **GO, KEGG, Yeastract transcriptome/proteome analysis (nitrogen)**. Logistic regression results for nitrogen up/down regulated genes/proteins from the transcriptome (trans) and proteome (prot) data using GO, KEGG and Yeastract annotations. Click here for file

Additional file 7 **GO, KEGG, Yeastract transcriptome/proteome analysis (phosphorus)**. Logistic regression results for phosphorus up/down regulated genes/proteins from the transcriptome (trans) and proteome (prot) data using GO, KEGG and Yeastract annotations. Click here for file

Additional file 8 **GO, KEGG, Yeastract transcriptome/proteome analysis (sulphur)**. Logistic regression results for sulphur up/down regulated genes/proteins from the transcriptome (trans) and proteome (prot) data using GO, KEGG and Yeastract annotations. Click here for file

Additional file 9 **Nutrient regulated GO biological process terms (transcriptome)**. GO biological process terms associated with up- (red) or down- (green) regulation of gene expression in one or more conditions (FDR < 1%). Click here for file

Additional file 10 **Nutrient regulated GO molecular function terms (transcriptome)**. GO molecular function terms associated with up- (red) or down- (green) regulation of gene expression in one or more conditions (FDR < 1%). Click here for file

Additional file 11 **Nutrient regulated GO cellular component terms (transcriptome)**. GO cellular component terms associated with up- (red) or down- (green) regulation of gene expression in one or more conditions (FDR < 1%). Click here for file

Additional file 12 **Nutrient regulated GO biological process terms (proteome)**. GO biological process terms associated with up- (red) or down- (green) regulation of protein levels in one or more conditions (FDR < 1%). Click here for file

Additional file 13 **Nutrient regulated GO molecular function terms (proteome)**. GO molecular function terms associated with up- (red) or down- (green) regulation of protein levels in one or more conditions (FDR < 1%). Click here for file

Additional file 14 **Nutrient regulated GO cellular component terms (proteome)**. GO cellular component terms associated with up- (red) or down- (green) regulation of protein levels in one or more conditions (FDR < 1%). Click here for file

Additional file 15 **Post-transcriptional control of YDR156W**. Protein and transcript log. fold changes in each nutrient-limiting condition relative to the overall mean for YDR156W. Click here for file

Additional file 16 **Post-transcriptional control of YLR174W**. Protein and transcript log. fold changes in each nutrient-limiting condition relative to the overall mean for YLR174W. Click here for file

Additional file 17 **Post-transcriptional control of YBR142W**. Protein and transcript log. fold changes in each nutrient-limiting condition relative to the overall mean for YBR142W. Click here for file

Additional file 18 **Post-transcriptional control of YMR083W**. Protein and transcript log. fold changes in each nutrient-limiting condition relative to the overall mean for YMR083W. Click here for file

Additional file 19 **Post-transcriptional control of YHR087W**. Protein and transcript log. fold changes in each nutrient-limiting condition relative to the overall mean for YHR087W. Click here for file

Additional file 20 **Post-transcriptional control of YBR117C**. Protein and transcript log. fold changes in each nutrient-limiting condition relative to the overall mean for YBR177C. Click here for file

Additional file 21 **Post-transcriptional control of YOL086C**. Protein and transcript log. fold changes in each nutrient-limiting condition relative to the overall mean for YOL086C. Click here for file

Additional file 22 **Post-transcriptional control of YGL258W**. Protein and transcript log. fold changes in each nutrient-limiting condition relative to the overall mean for YGL258W. Click here for file

Additional file 23 **Post-transcriptional control of YDR345C**. Protein and transcript log. fold changes in each nutrient-limiting condition relative to the overall mean for YDR345C. Click here for file

Additional file 24 **Post-transcriptional control of YLR029C**. Protein and transcript log. fold changes in each nutrient-limiting condition relative to the overall mean for YL029C. Click here for file

Additional file 25 **Post-transcriptional control of YJR152W**. Protein and transcript log. fold changes in each nutrient-limiting condition relative to the overall mean for YJR152W. Click here for file

Additional file 26 **GO, KEGG, Yeastract transcriptome/proteome analysis (growth rate carbon)**. Logistic regression results for growth rate up/down regulated genes/proteins in carbon limitation from the transcriptome (trans) and proteome (prot) data using GO, KEGG and Yeastract annotations. Click here for file

Additional file 27 **GO, KEGG, Yeastract transcriptome/proteome analysis (growth rate nitrogen)**. Logistic regression results for growth rate up/down regulated genes/proteins in nitrogen limitation from the transcriptome (trans) and proteome (prot) data using GO, KEGG and Yeastract annotations. Click here for file

Additional file 28 **GO, KEGG, Yeastract transcriptome/proteome analysis (growth rate phosphorus)**. Logistic regression results for growth rate up/down regulated genes/proteins in phosphorus limitation from the transcriptome (trans) and proteome (prot) data using GO, KEGG and Yeastract annotations. Click here for file

Additional file 29 **GO, KEGG, Yeastract transcriptome/proteome analysis (growth rate sulphur)**. Logistic regression results for growth rate up/down regulated genes/proteins in sulphur limitation from the transcriptome (trans) and proteome (prot) data using GO, KEGG and Yeastract annotations. Click here for file

Additional file 30 **Growth rate regulated GO biological process terms (transcriptome)**. GO biological process terms associated with up- (red) or down- (green) regulation of gene expression with changes in growth rate in one or more conditions (FDR < 1%). Click here for file

Additional file 31 **Growth rate regulated GO molecular function terms (transcriptome)**. GO molecular function terms associated with up- (red) or down- (green) regulation of gene expression with changes in growth rate in one or more conditions (FDR < 1%). Click here for file

Additional file 32 **Growth rate regulated GO cellular component terms (transcriptome)**. GO cellular component terms associated with up- (red) or down- (green) regulation of gene expression with changes in growth rate in one or more conditions (FDR < 1%). Click here for file

Additional file 33 **Growth rate regulated GO biological process terms (proteome)**. GO biological process terms associated with up- (red) or down- (green) regulation of protein level with changes in growth rate in one or more conditions (FDR < 1%). Click here for file

Additional file 34 **Growth rate regulated GO molecular function terms (proteome)**. GO molecular function terms associated with up- (red) or down- (green) regulation of protein level with changes in growth rate in one or more conditions (FDR < 1%). Click here for file

Additional file 35 **Growth rate regulated GO cellular component terms (proteome)**. GO cellular component terms associated with up- (red) or down- (green) regulation of protein level with changes in growth rate in one or more conditions (FDR < 1%). Click here for file

Additional file 36 **ORFs under growth rate regulated post-transcriptional control**. Individual genes that show significant changes in translational control efficiency between growth rates in each nutrient limitation. Colours indicate the log. ratio of translational control efficiency between the two growth rates. Only genes with a more than two-fold change in translational efficiency are shown. Click here for file

Additional file 37 **Post-transcriptional control of YPR184W**. Protein and transcript log. fold changes for the shift from D = 0.1 h^-1 ^to D = 0.2 h^-1 ^in each nutrient-limiting condition for YPR184W. Click here for file

Additional file 38 **Post-transcriptional control of YLR034C**. Protein and transcript log. fold changes for the shift from D = 0.1 h^-1 ^to D = 0.2 h^-1 ^in each nutrient-limiting condition for YLR034C. Click here for file

Additional file 39 **Post-transcriptional control of YLR285W**. Protein and transcript log. fold changes for the shift from D = 0.1 h^-1 ^to D = 0.2 h^-1 ^in each nutrient-limiting condition for YLR285W. Click here for file

Additional file 40 **Post-transcriptional control of YPR160W**. Protein and transcript log. fold changes for the shift from D = 0.1 h^-1 ^to D = 0.2 h^-1 ^in each nutrient-limiting condition for YPR160W. Click here for file

Additional file 41 **Post-transcriptional control of YGR175C**. Protein and transcript log. fold changes for the shift from D = 0.1 h^-1 ^to D = 0.2 h^-1 ^in each nutrient-limiting condition for YGR175C. Click here for file

Additional file 42 **Post-transcriptional control of YOR303W**. Protein and transcript log. fold changes for the shift from D = 0.1 h^-1 ^to D = 0.2 h^-1 ^in each nutrient-limiting condition for YOR303W. Click here for file

Additional file 43 **Post-transcriptional control of YDL171C**. Protein and transcript log. fold changes for the shift from D = 0.1 h^-1 ^to D = 0.2 h^-1 ^in each nutrient-limiting condition for YDL171C. Click here for file

Additional file 44 **Post-transcriptional control of YPL240C**. Protein and transcript log. fold changes for the shift from D = 0.1 h^-1 ^to D = 0.2 h^-1 ^in each nutrient-limiting condition for YPL240C. Click here for file

Additional file 45 **Post-transcriptional control of YCL011C**. Protein and transcript log. fold changes for the shift from D = 0.1 h^-1 ^to D = 0.2 h^-1 ^in each nutrient-limiting condition for YCL011C. Click here for file

Additional file 46 **Post-transcriptional control of YGR087C**. Protein and transcript log. fold changes for the shift from D = 0.1 h^-1 ^to D = 0.2 h^-1 ^in each nutrient-limiting condition for YGR087C. Click here for file

Additional file 47 **Post-transcriptional control of YBR072W**. Protein and transcript log. fold changes for the shift from D = 0.1 h^-1 ^to D = 0.2 h^-1 ^in each nutrient-limiting condition for YBR072W. Click here for file

Additional file 48 **Post-transcriptional control of YMR083W**. Protein and transcript log. fold changes for the shift from D = 0.1 h^-1 ^to D = 0.2 h^-1 ^in each nutrient-limiting condition for YMR083W. Click here for file

Additional file 49 **Post-transcriptional control of YOL151W**. Protein and transcript log. fold changes for the shift from D = 0.1 h^-1 ^to D = 0.2 h^-1 ^in each nutrient-limiting condition for YOL151W. Click here for file

Additional file 50 **Post-transcriptional control of YER021W**. Protein and transcript log. fold changes for the shift from D = 0.1 h^-1 ^to D = 0.2 h^-1 ^in each nutrient-limiting condition for YER021W. Click here for file

Additional file 51 **Post-transcriptional control of YGL068W**. Protein and transcript log. fold changes for the shift from D = 0.1 h^-1 ^to D = 0.2 h^-1 ^in each nutrient-limiting condition for YGL068W. Click here for file

Additional file 52 **Post-transcriptional control of YDR155C**. Protein and transcript log. fold changes for the shift from D = 0.1 h^-1 ^to D = 0.2 h^-1 ^in each nutrient-limiting condition for YDR155C. Click here for file

Additional file 53 **Physiological parameters**. Specific rates of glucose comsumption (q_gluc_) and ethanol production (q_ethanol_) from the chemostat series under specific nutrient-limiting conditions. Click here for file

Additional file 54 **GO, KEGG, Yeastract transcriptome/proteome analysis (growth rate carbon specific)**. Logistic regression results for growth rate up/down regulated genes/proteins in carbon limitation relative to the overall growth rate effect from the transcriptome (trans) and proteome (prot) data using GO, KEGG and Yeastract annotations. Click here for file

Additional file 55 **GO, KEGG, Yeastract transcriptome/proteome analysis (growth rate nitrogen specific)**. Logistic regression results for growth rate up/down regulated genes/proteins in nitrogen limitation relative to the overall growth rate effect from the transcriptome (trans) and proteome (prot) data using GO, KEGG and Yeastract annotations. Click here for file

Additional file 56 **GO, KEGG, Yeastract transcriptome/proteome analysis (growth rate phosphorus specific)**. Logistic regression results for growth rate up/down regulated genes/proteins in phosphorus limitation relative to the overall growth rate effect from the transcriptome (trans) and proteome (prot) data using GO, KEGG and Yeastract annotations. Click here for file

Additional file 57 **GO, KEGG, Yeastract transcriptome/proteome analysis (growth rate sulphur specific)**. Logistic regression results for growth rate up/down regulated genes/proteins in sulphur limitation relative to the overall growth rate effect from the transcriptome (trans) and proteome (prot) data using GO, KEGG and Yeastract annotations. Click here for file

Additional file 58 **Nutrient specific growth rate regulated GO biological process terms (transcriptome)**. GO biological process terms associated with up- (red) or down- (green) regulation of gene expression with increasing growth rate in one or more conditions relative to the overall trend (FDR < 1%). Click here for file

Additional file 59 **Nutrient specific growth rate regulated GO molecular function terms (transcriptome)**. GO molecular function terms associated with up- (red) or down- (green) regulation of gene expression with increasing growth rate in one or more conditions relative to the overall trend (FDR < 1%). Click here for file

Additional file 60 **Nutrient specific growth rate regulated GO cellular component terms (transcriptome)**. GO cellular component terms associated with up- (red) or down- (green) regulation of gene expression with increasing growth rate in one or more conditions relative to the overall trend (FDR < 1%). Click here for file

Additional file 61 **Logistic regression example**. Association of positive slopes of regression with the ribosome biogenesis GO term. Slopes are calculated for each gene from a linear regression of gene expression against growth rate. Vertical tick marks show the slopes of ribosome biogenesis annotated genes. The proportion of all genes at a given slope that are annotated with the term is shown with the dashed line. The solid line shows the fitted logistic regression. Click here for file
