# Supplementary figures and images for: Nutrient control of eukaryote cell growth: a systems biology study in yeast
Source: BMC Biol. 2010 May 24;8:68. doi: 10.1186/1741-7007-8-68 (PMC2895586; doi:10.1186/1741-7007-8-68)

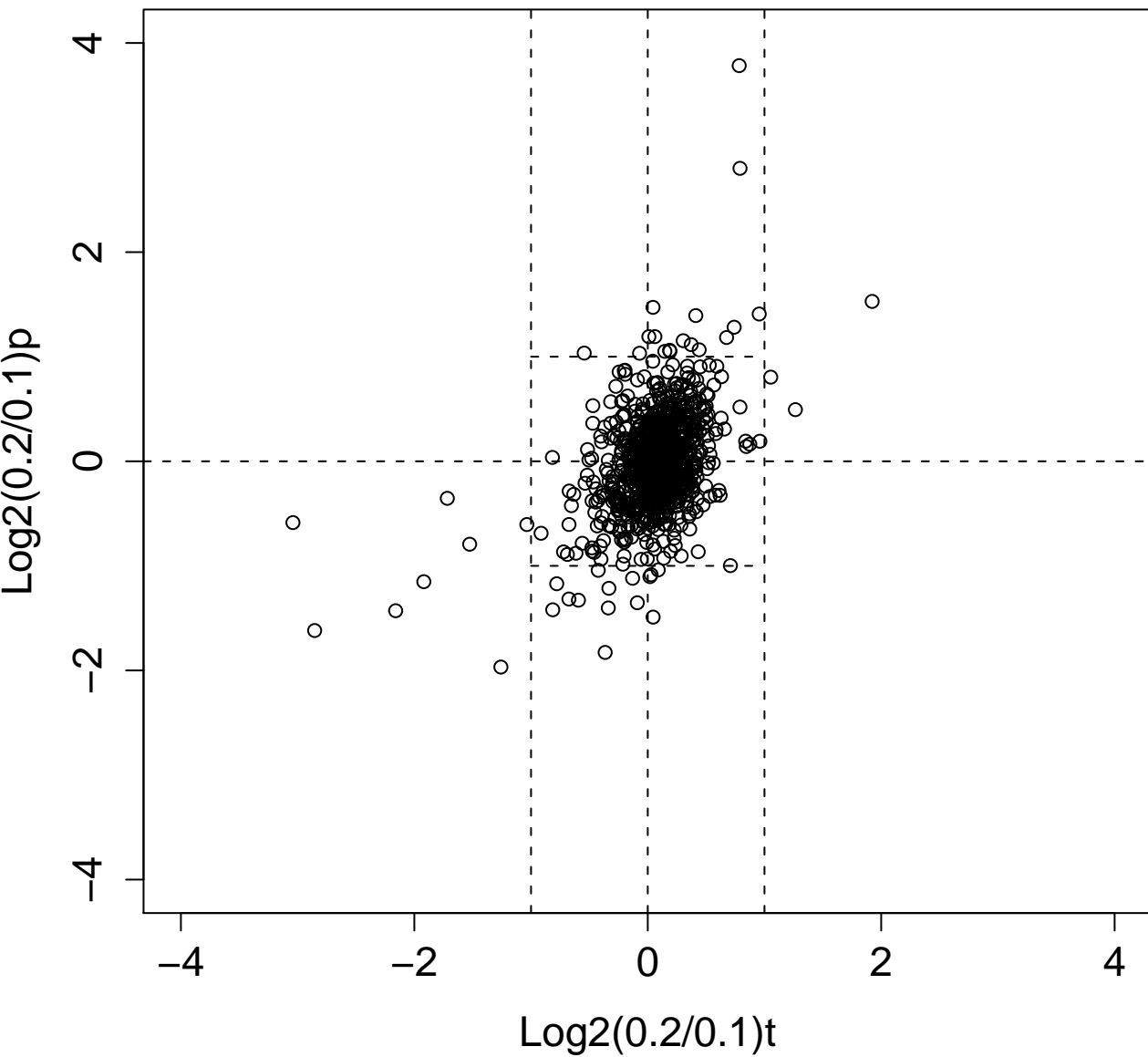

Supplement: Additional file 1 — Proteome/transcriptome correlation (carbon). Log. fold changes on increasing growth rate from D = 0.1 h-1 to D = 0.2 h-1 for protein (p) and gene expression (t) levels in carbon limitation. [file 1741-7007-8-68-S1.pdf]

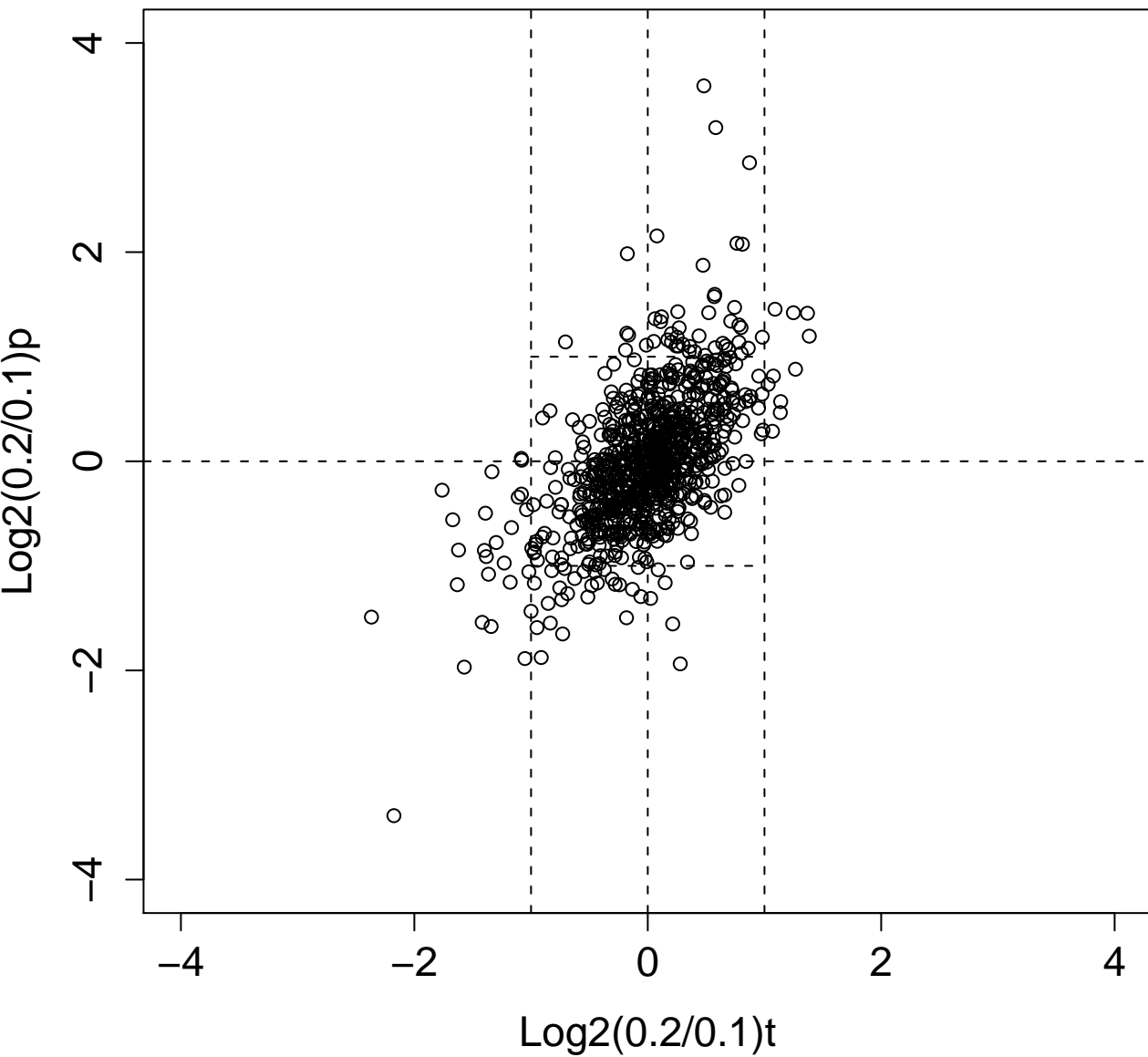

Supplement: Additional file 2 — Proteome/transcriptome correlation (nitrogen). Log. fold changes on increasing growth rate from D = 0.1 h-1 to D = 0.2 h-1 for protein (p) and gene expression (t) levels in nitrogen limitation. [file 1741-7007-8-68-S2.pdf]

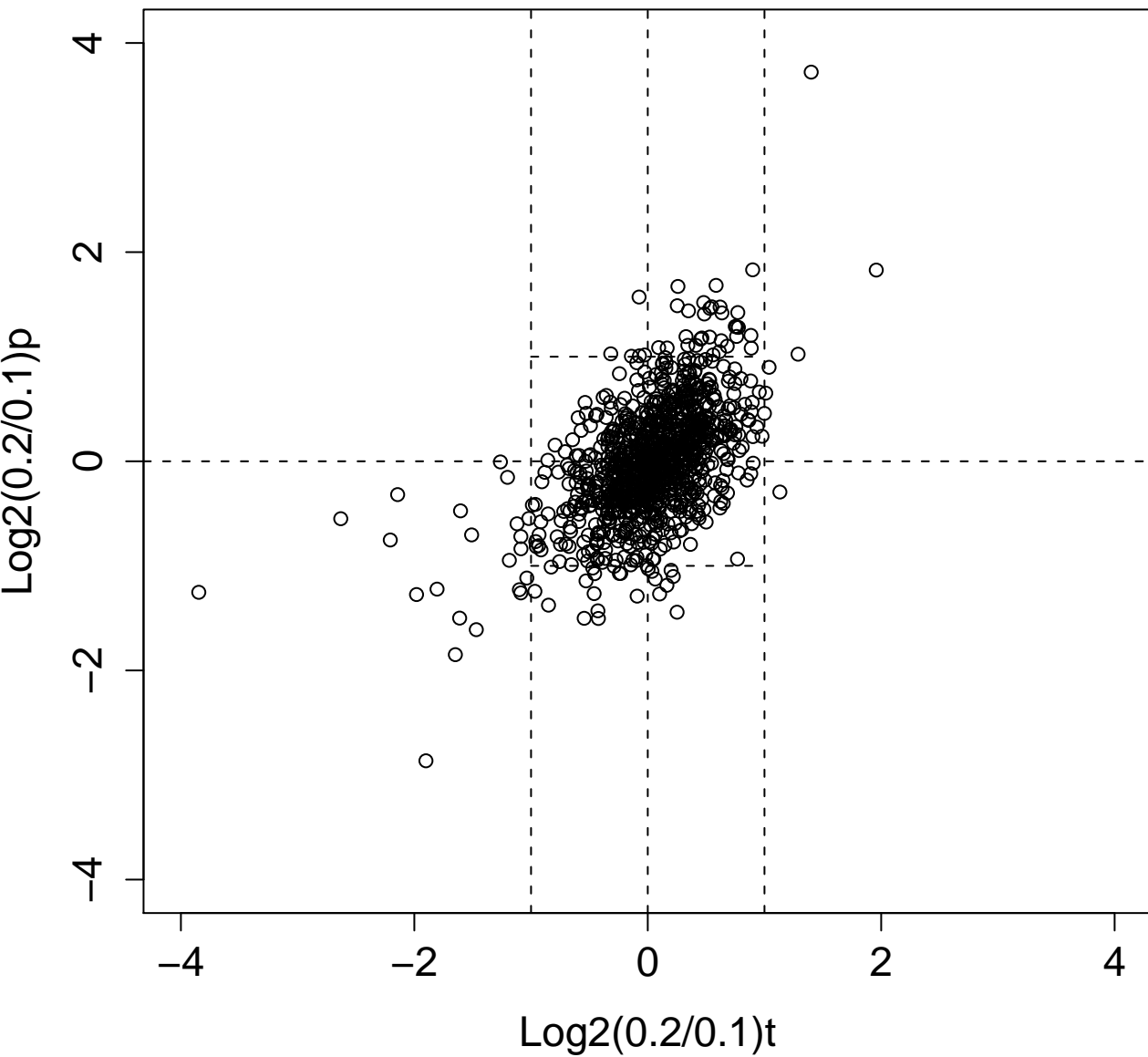

Supplement: Additional file 3 — Proteome/transcriptome correlation (phosphorus). Log. fold changes on increasing growth rate from D = 0.1 h-1 to D = 0.2 h-1 for protein (p) and gene expression (t) levels in phosphorus limitation. [file 1741-7007-8-68-S3.pdf]

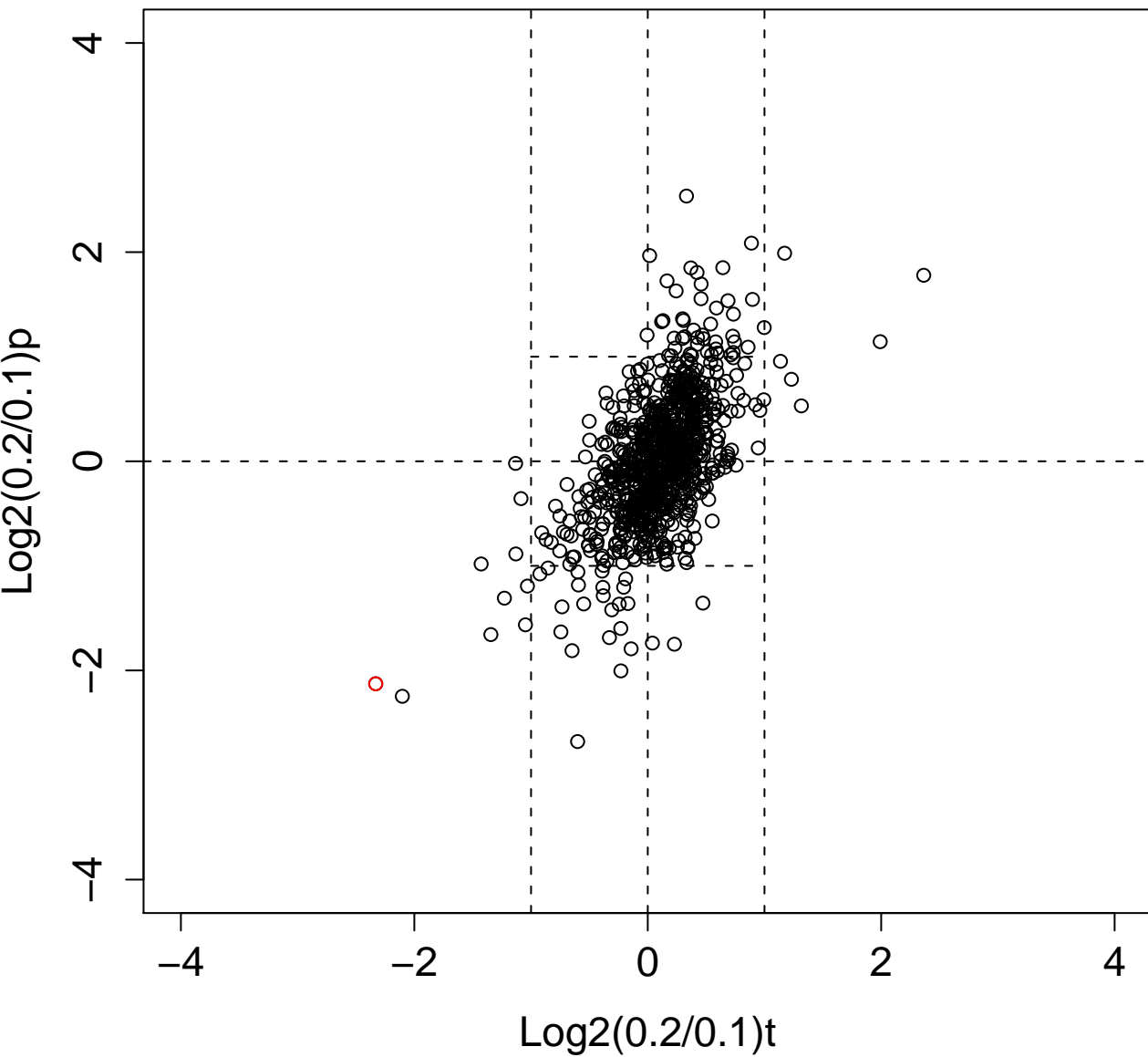

Supplement: Additional file 4 — Proteome/transcriptome correlation (sulphur). Log. fold changes on increasing growth rate from D = 0.1 h-1 to D = 0.2 h-1 for protein (p) and gene expression (t) levels in sulphur limitation. [file 1741-7007-8-68-S4.pdf]

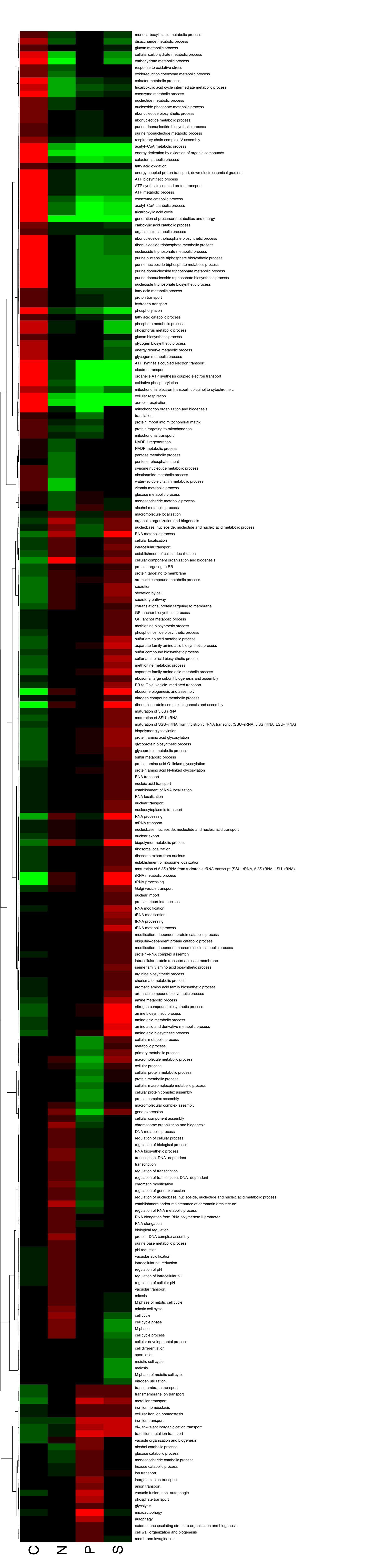

Supplement: Additional file 9 — Nutrient regulated GO biological process terms (transcriptome). GO biological process terms associated with up- (red) or down- (green) regulation of gene expression in one or more conditions (FDR < 1%). [file 1741-7007-8-68-S9.pdf]

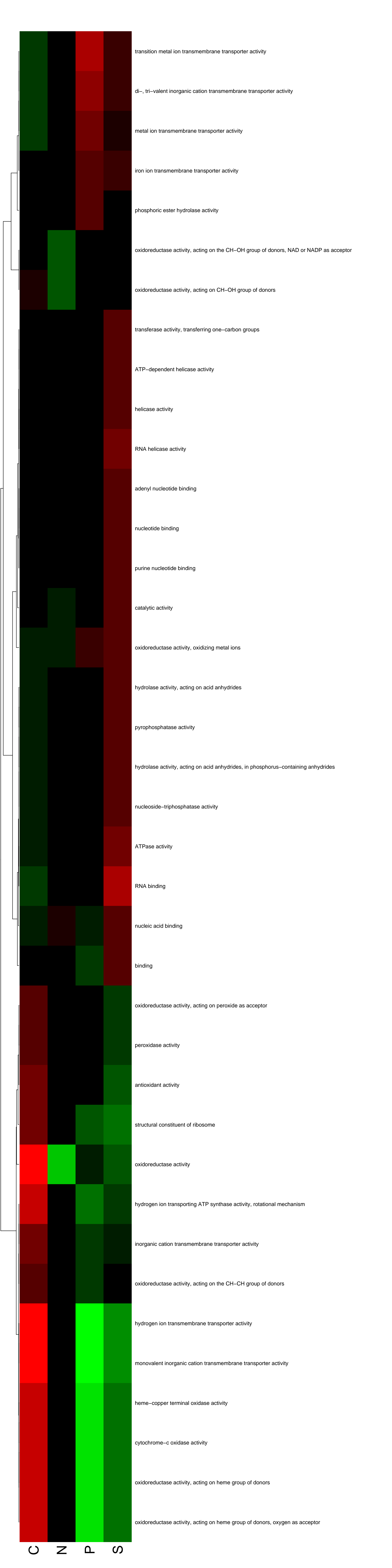

Supplement: Additional file 10 — Nutrient regulated GO molecular function terms (transcriptome). GO molecular function terms associated with up- (red) or down- (green) regulation of gene expression in one or more conditions (FDR < 1%). [file 1741-7007-8-68-S10.pdf]

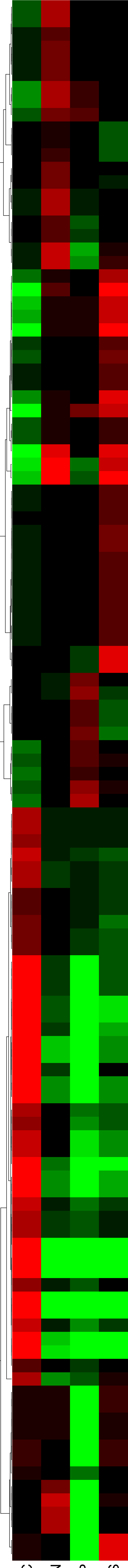

Supplement: Additional file 11 — Nutrient regulated GO cellular component terms (transcriptome). GO cellular component terms associated with up- (red) or down- (green) regulation of gene expression in one or more conditions (FDR < 1%). [file 1741-7007-8-68-S11.pdf]

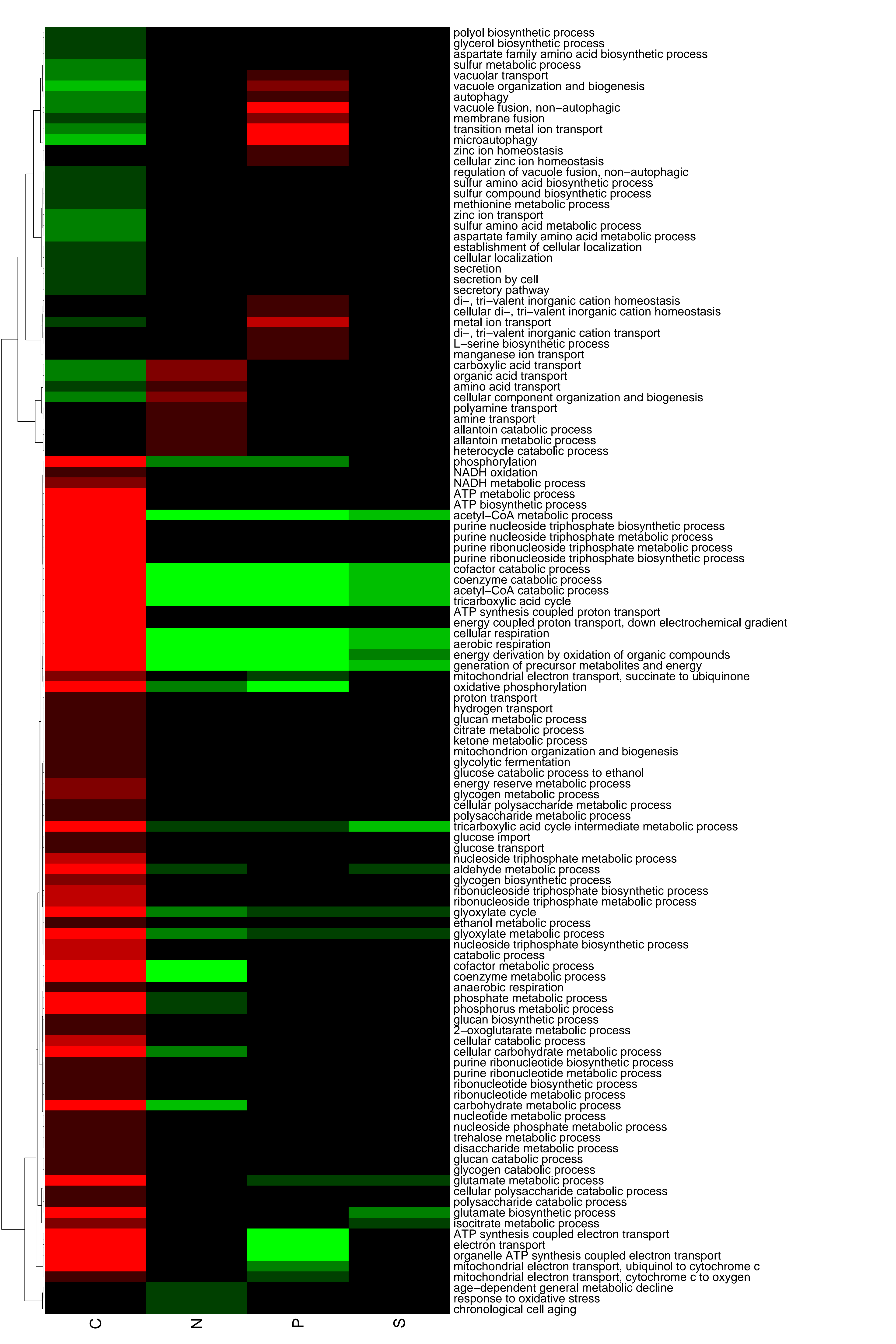

Supplement: Additional file 12 — Nutrient regulated GO biological process terms (proteome). GO biological process terms associated with up- (red) or down- (green) regulation of protein levels in one or more conditions (FDR < 1%). [file 1741-7007-8-68-S12.pdf]

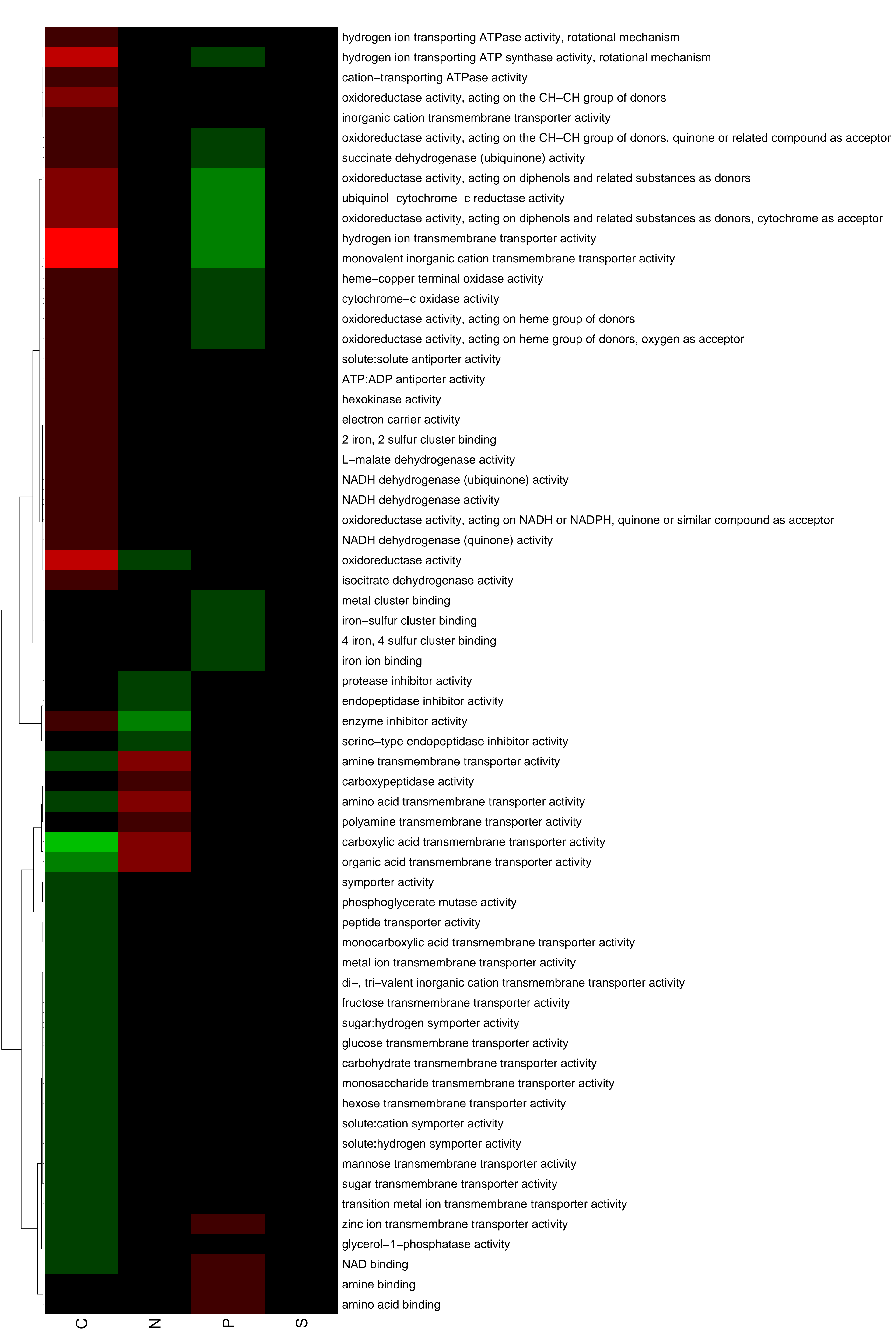

Supplement: Additional file 13 — Nutrient regulated GO molecular function terms (proteome). GO molecular function terms associated with up- (red) or down- (green) regulation of protein levels in one or more conditions (FDR < 1%). [file 1741-7007-8-68-S13.pdf]

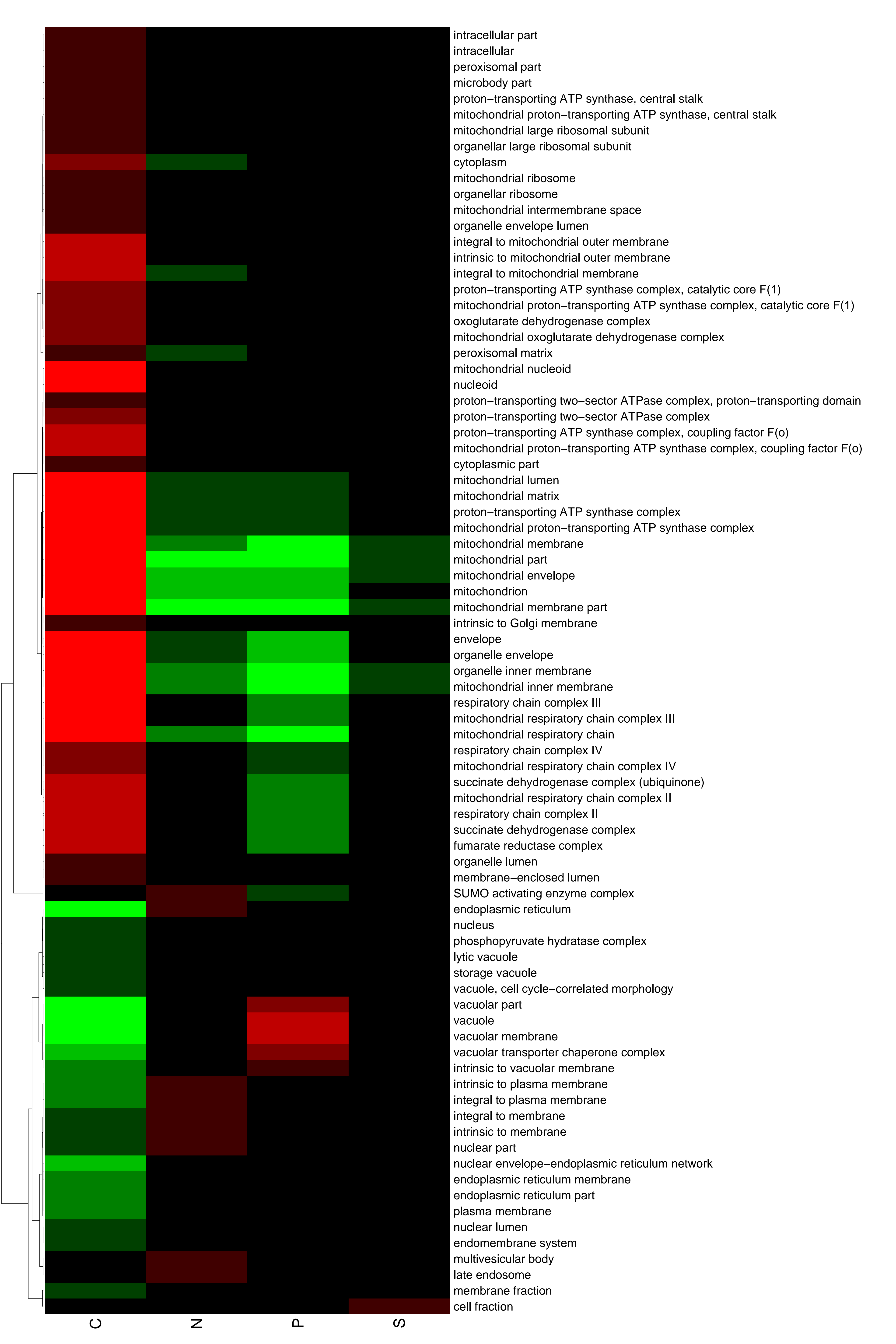

Supplement: Additional file 14 — Nutrient regulated GO cellular component terms (proteome). GO cellular component terms associated with up- (red) or down- (green) regulation of protein levels in one or more conditions (FDR < 1%). [file 1741-7007-8-68-S14.pdf]

# YDR156W

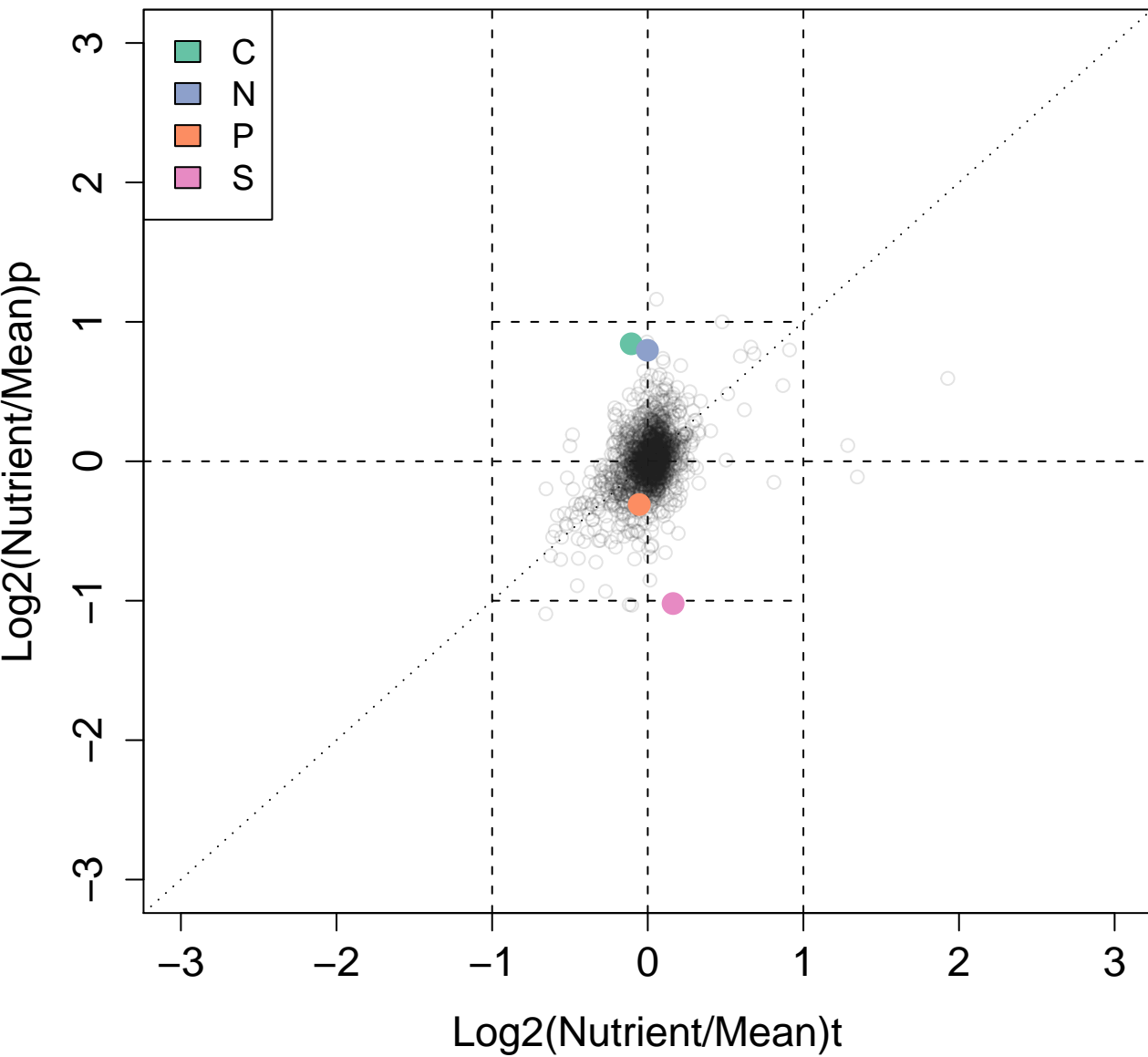

Supplement: Additional file 15 — Post-transcriptional control of YDR156W. Protein and transcript log. fold changes in each nutrient-limiting condition relative to the overall mean for YDR156W. [file 1741-7007-8-68-S15.pdf]

# YLR174W

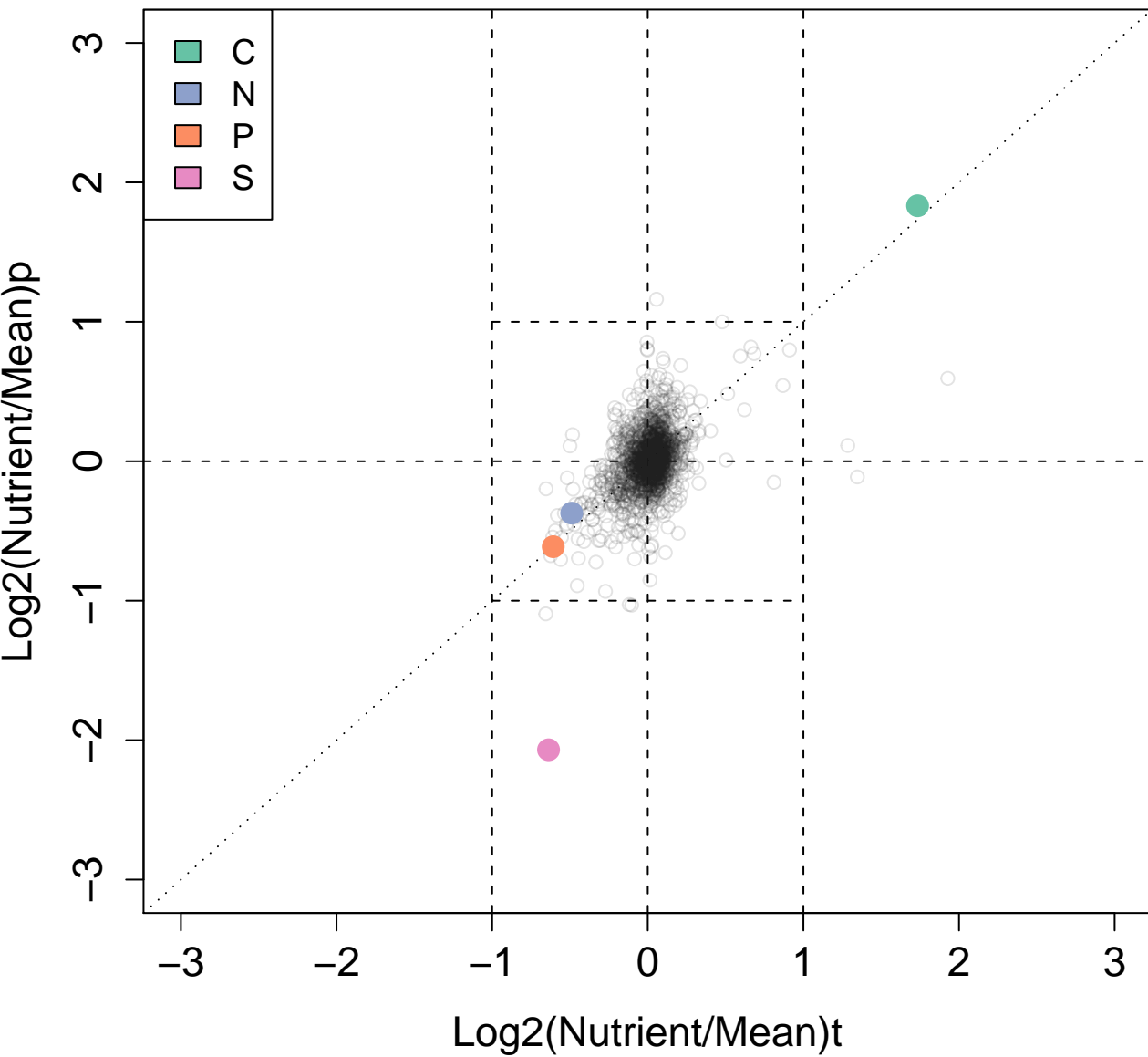

Supplement: Additional file 16 — Post-transcriptional control of YLR174W. Protein and transcript log. fold changes in each nutrient-limiting condition relative to the overall mean for YLR174W. [file 1741-7007-8-68-S16.pdf]

# YBR142W

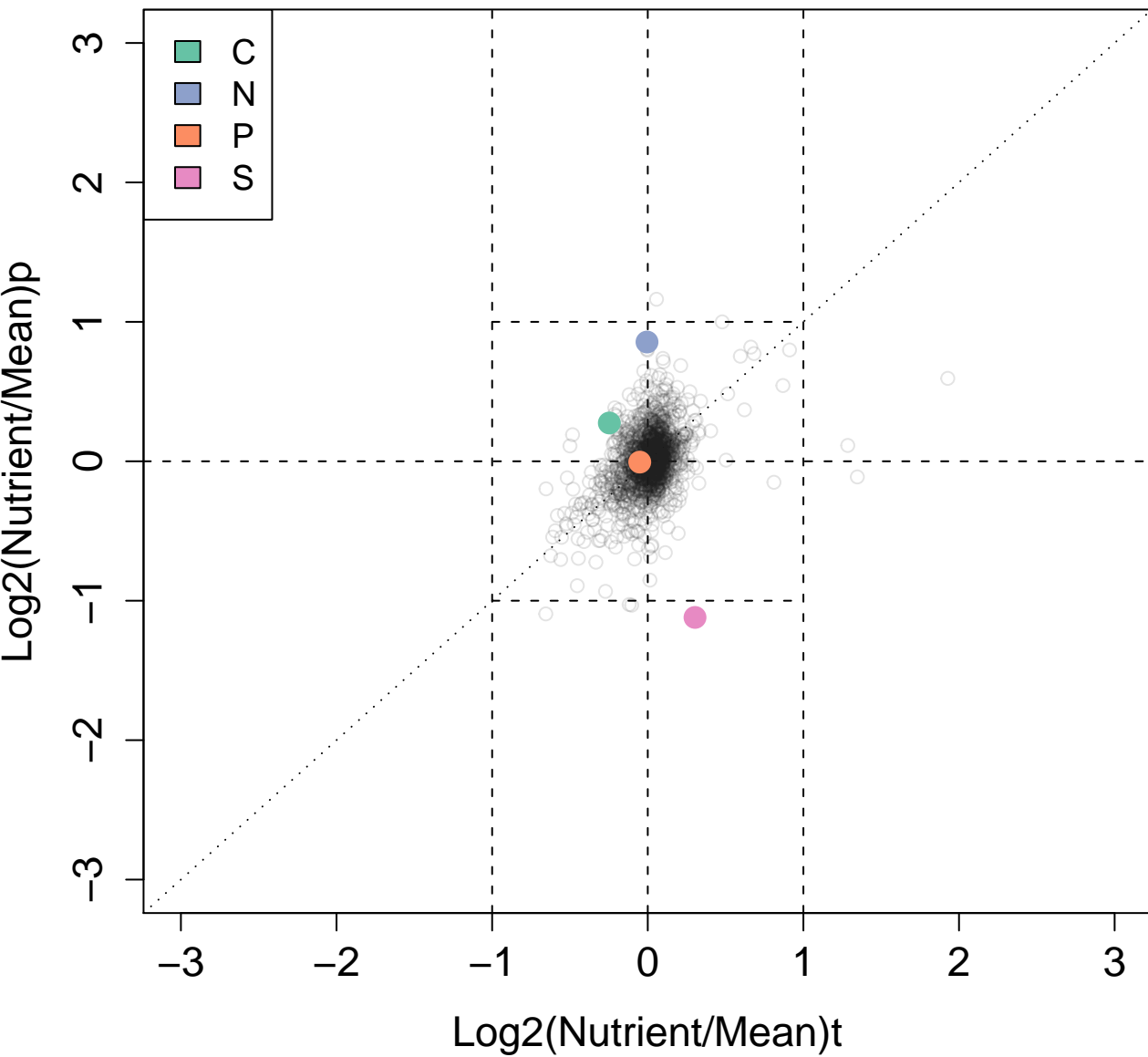

Supplement: Additional file 17 — Post-transcriptional control of YBR142W. Protein and transcript log. fold changes in each nutrient-limiting condition relative to the overall mean for YBR142W. [file 1741-7007-8-68-S17.pdf]

# YMR083W

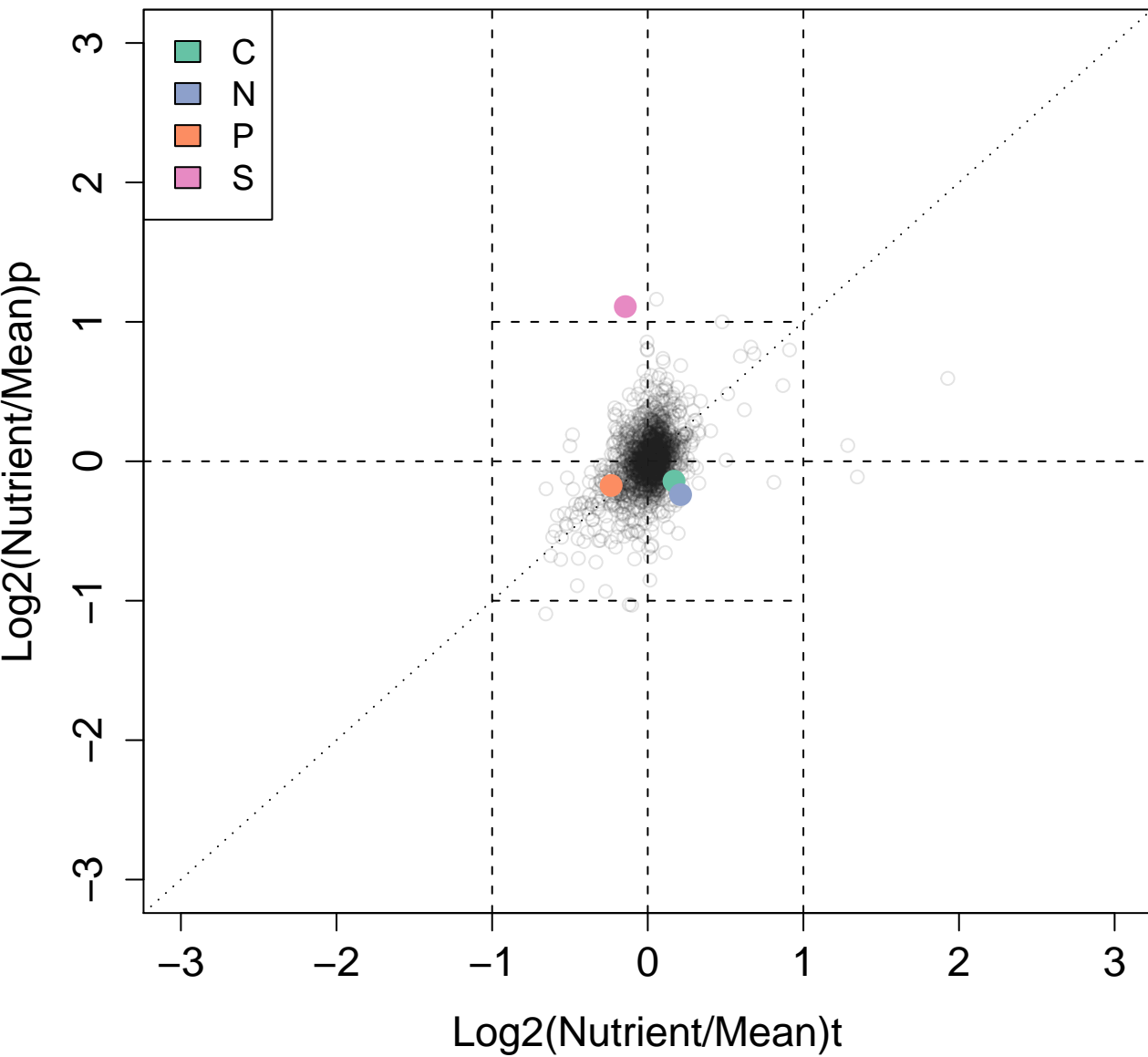

Supplement: Additional file 18 — Post-transcriptional control of YMR083W. Protein and transcript log. fold changes in each nutrient-limiting condition relative to the overall mean for YMR083W. [file 1741-7007-8-68-S18.pdf]

# YHR087W

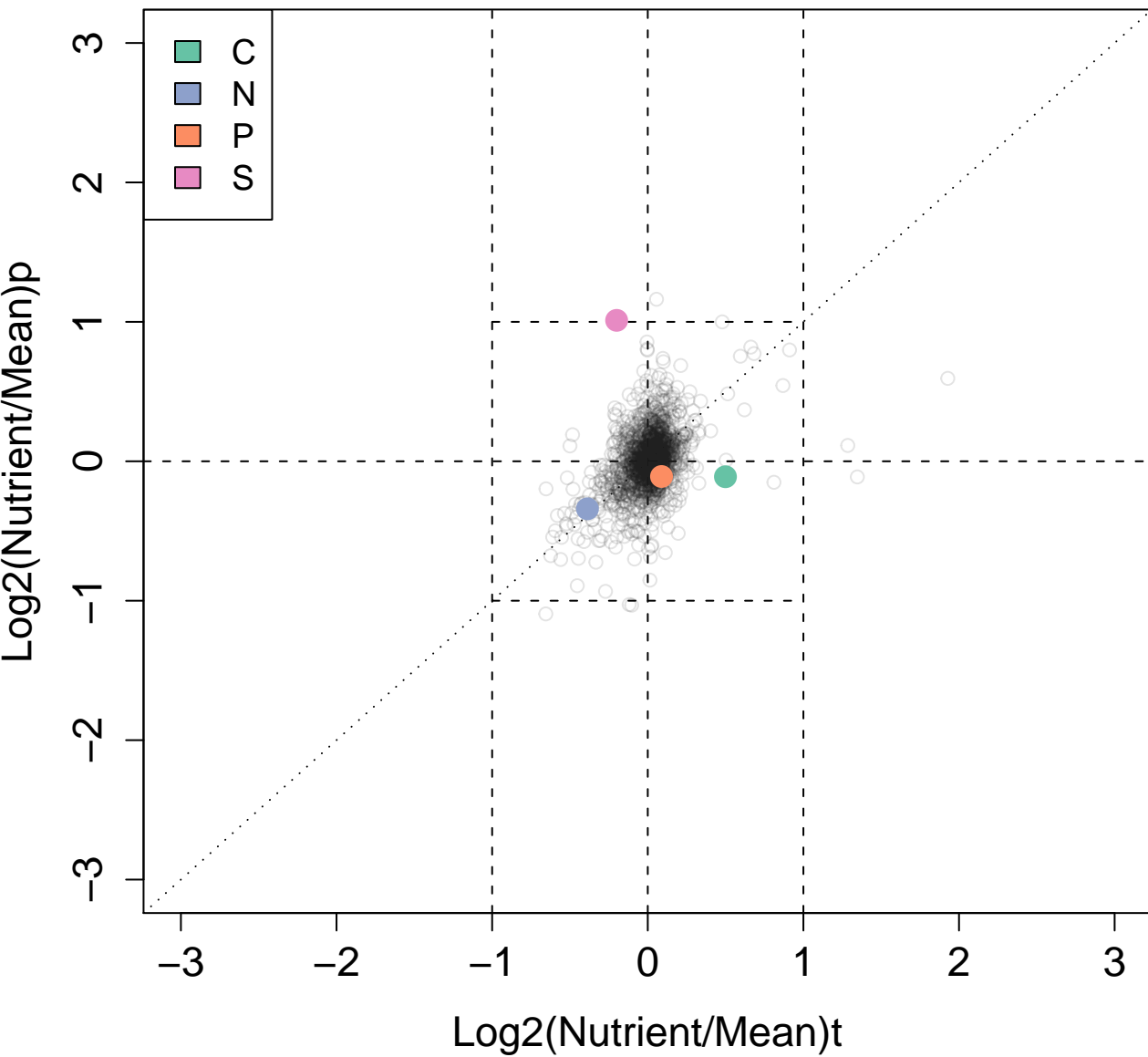

Supplement: Additional file 19 — Post-transcriptional control of YHR087W. Protein and transcript log. fold changes in each nutrient-limiting condition relative to the overall mean for YHR087W. [file 1741-7007-8-68-S19.pdf]

# YBR117C

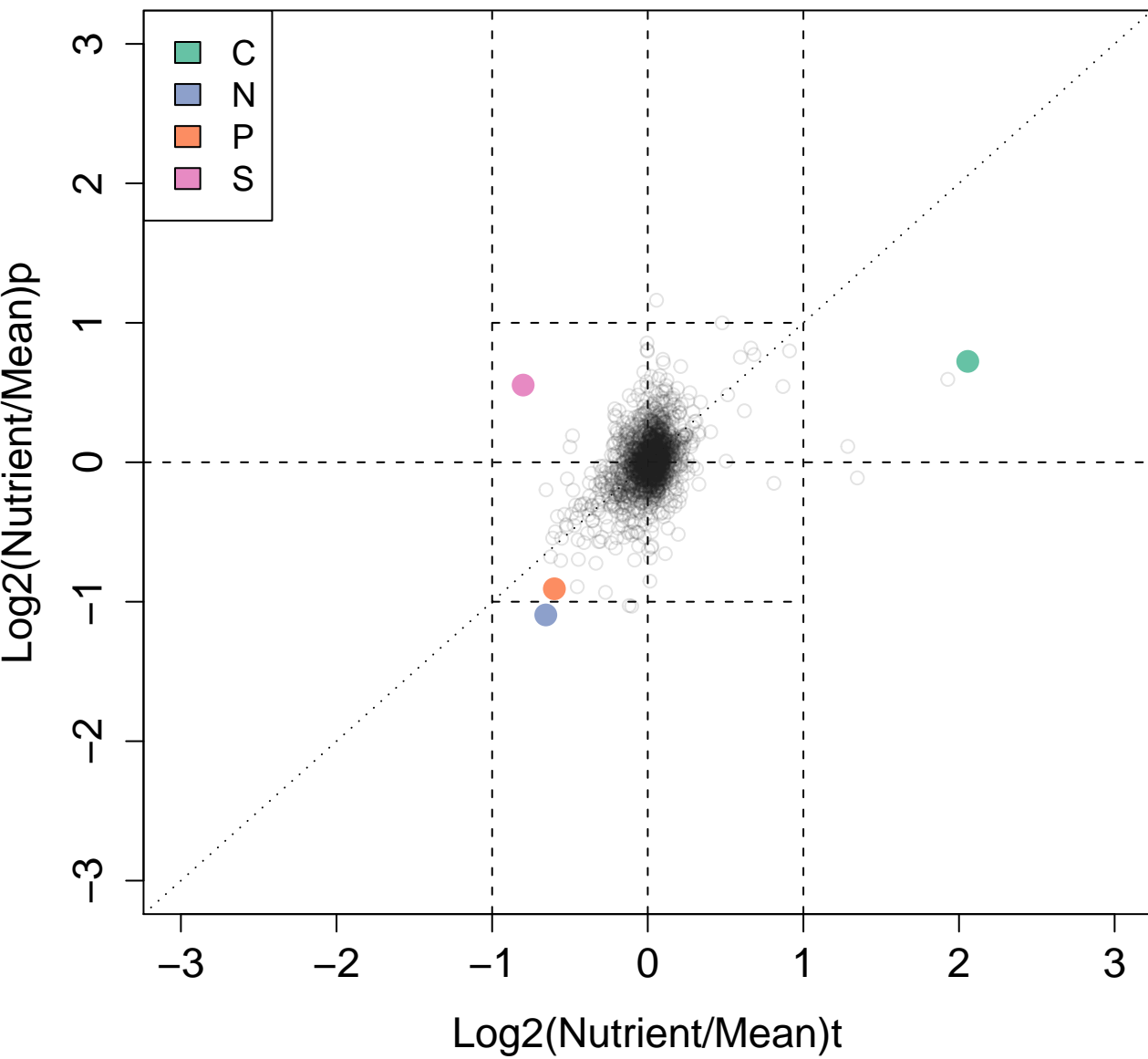

Supplement: Additional file 20 — Post-transcriptional control of YBR117C. Protein and transcript log. fold changes in each nutrient-limiting condition relative to the overall mean for YBR177C. [file 1741-7007-8-68-S20.pdf]

# YOL086C

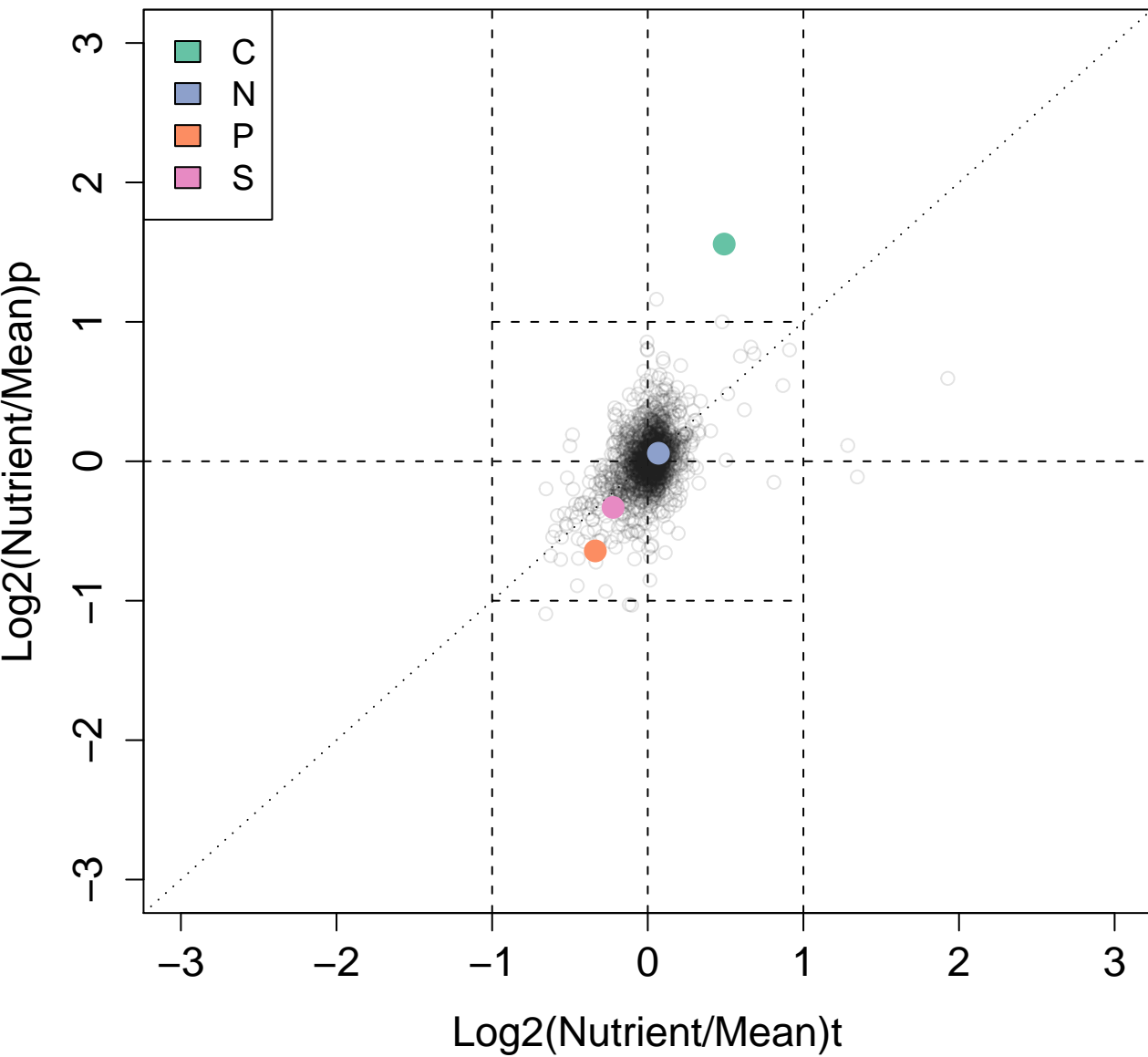

Supplement: Additional file 21 — Post-transcriptional control of YOL086C. Protein and transcript log. fold changes in each nutrient-limiting condition relative to the overall mean for YOL086C. [file 1741-7007-8-68-S21.pdf]

# YGL258W

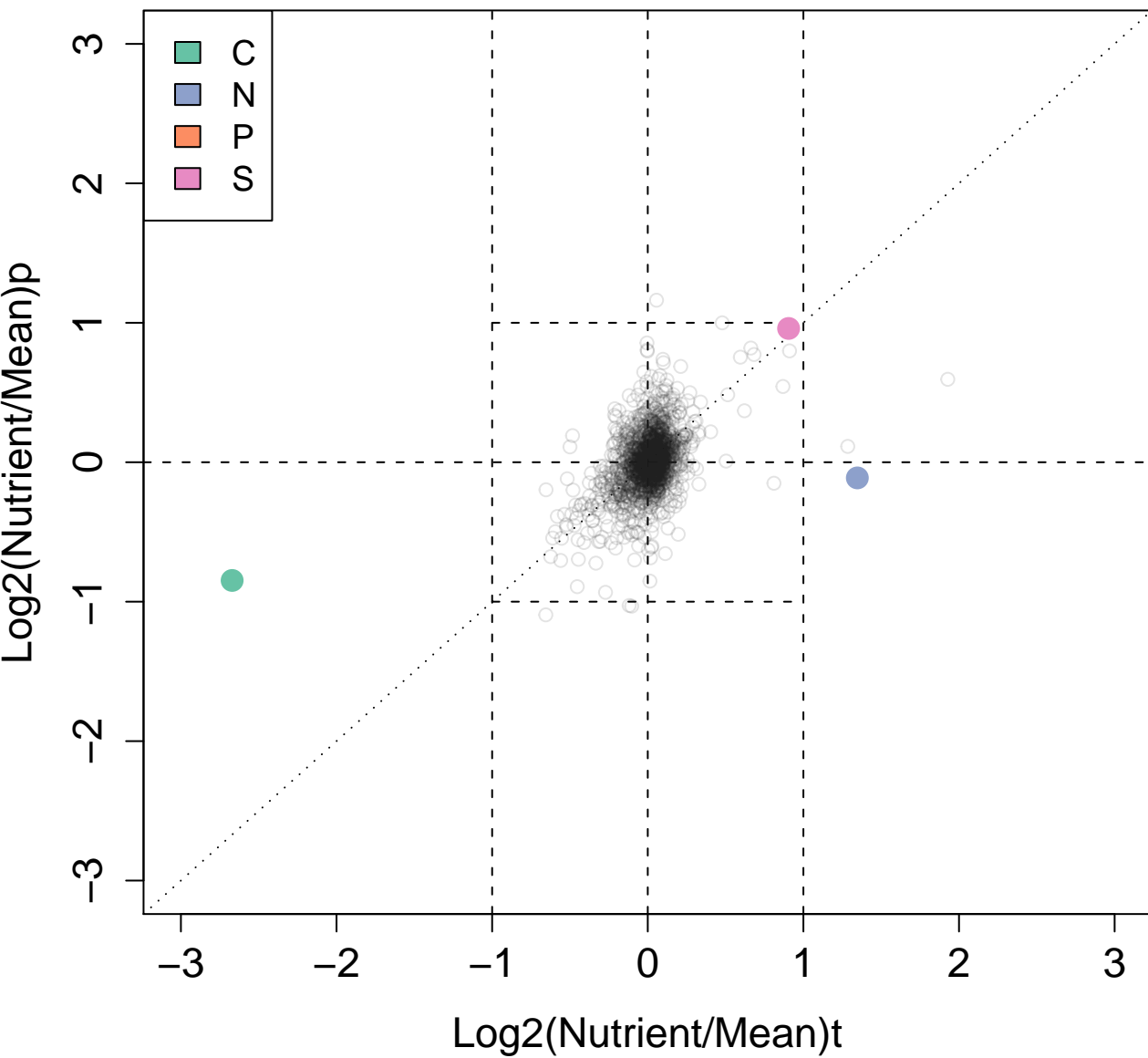

Supplement: Additional file 22 — Post-transcriptional control of YGL258W. Protein and transcript log. fold changes in each nutrient-limiting condition relative to the overall mean for YGL258W. [file 1741-7007-8-68-S22.pdf]

# YDR345C

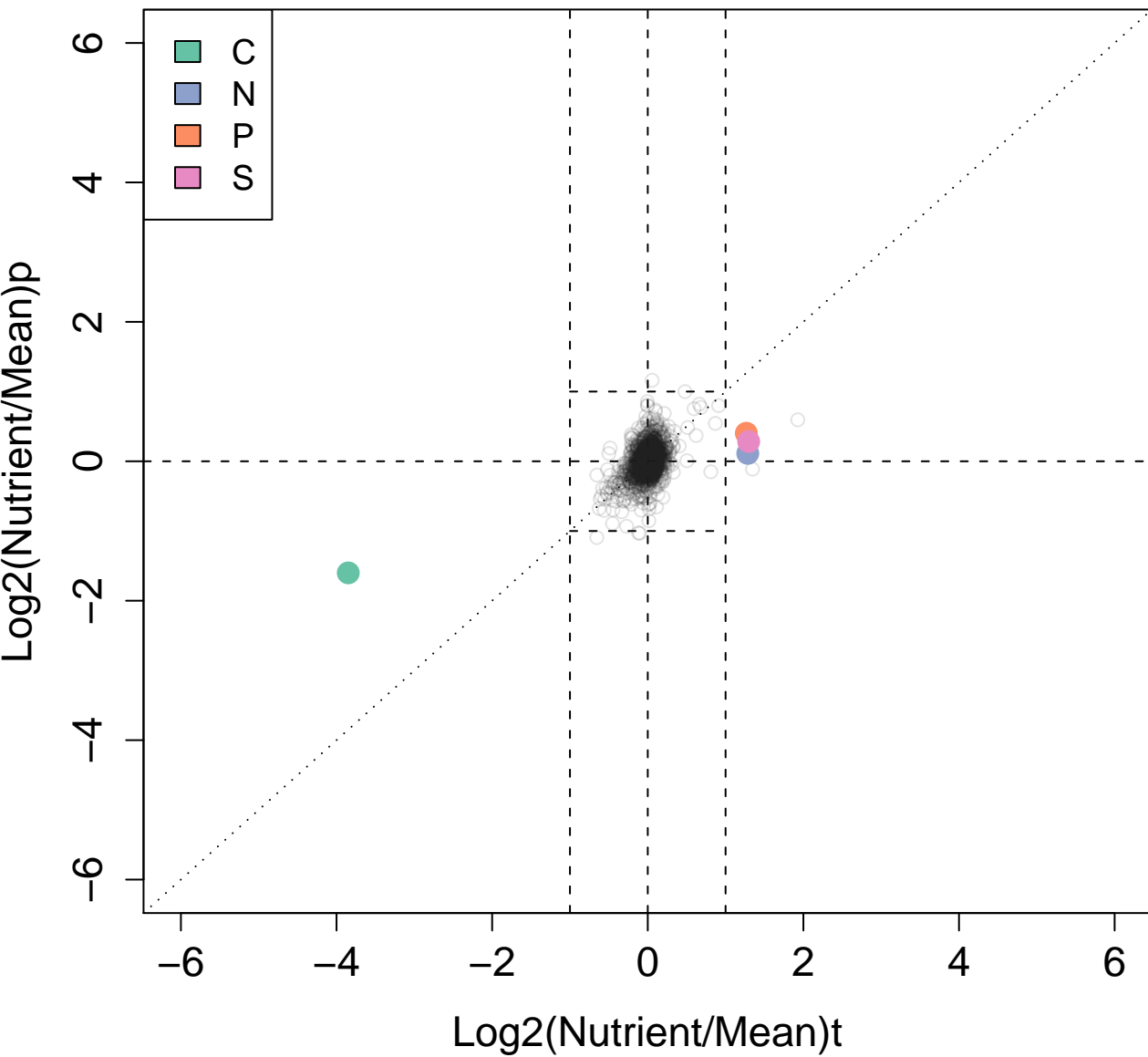

Supplement: Additional file 23 — Post-transcriptional control of YDR345C. Protein and transcript log. fold changes in each nutrient-limiting condition relative to the overall mean for YDR345C. [file 1741-7007-8-68-S23.pdf]

# YLR029C

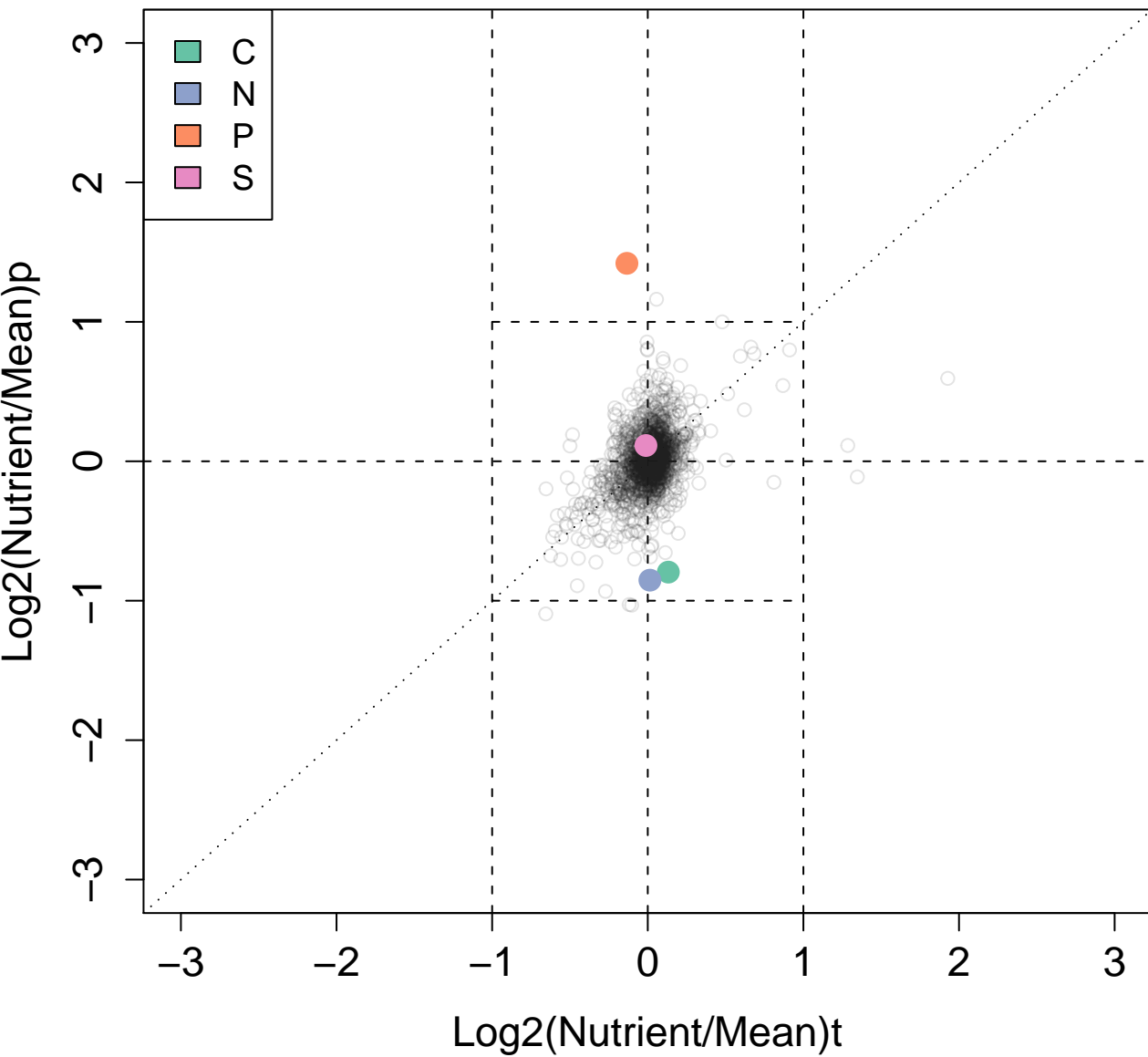

Supplement: Additional file 24 — Post-transcriptional control of YLR029C. Protein and transcript log. fold changes in each nutrient-limiting condition relative to the overall mean for YL029C. [file 1741-7007-8-68-S24.pdf]

# YJR152W

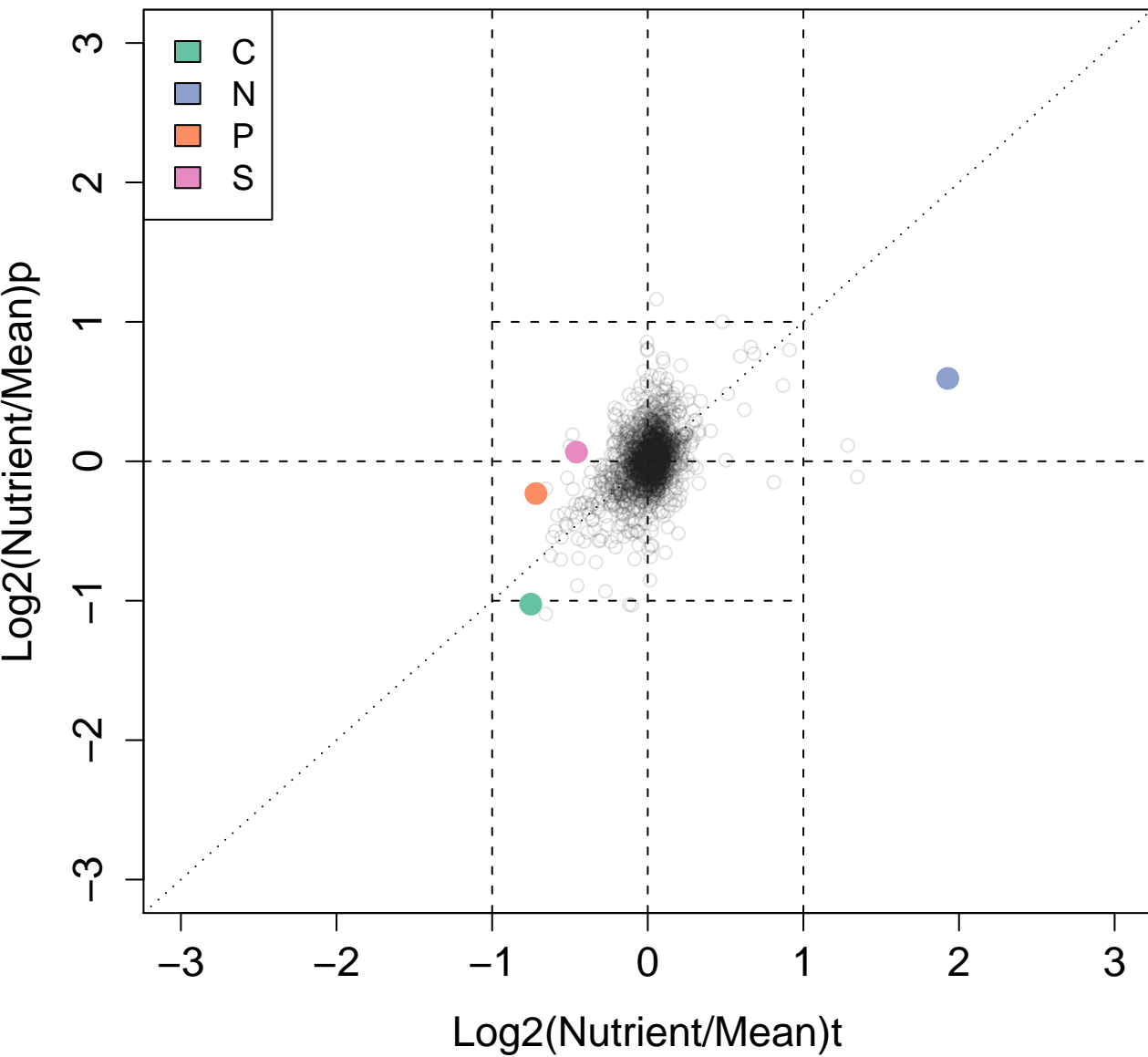

Supplement: Additional file 25 — Post-transcriptional control of YJR152W. Protein and transcript log. fold changes in each nutrient-limiting condition relative to the overall mean for YJR152W. [file 1741-7007-8-68-S25.pdf]

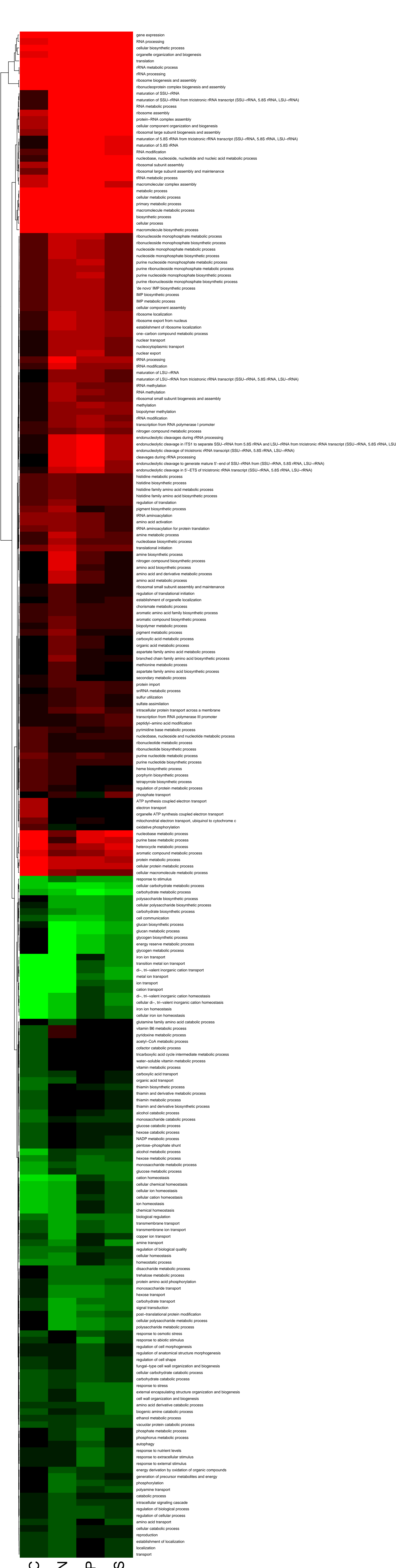

Supplement: Additional file 30 — Growth rate regulated GO biological process terms (transcriptome). GO biological process terms associated with up- (red) or down- (green) regulation of gene expression with changes in growth rate in one or more conditions (FDR < 1%). [file 1741-7007-8-68-S30.pdf]

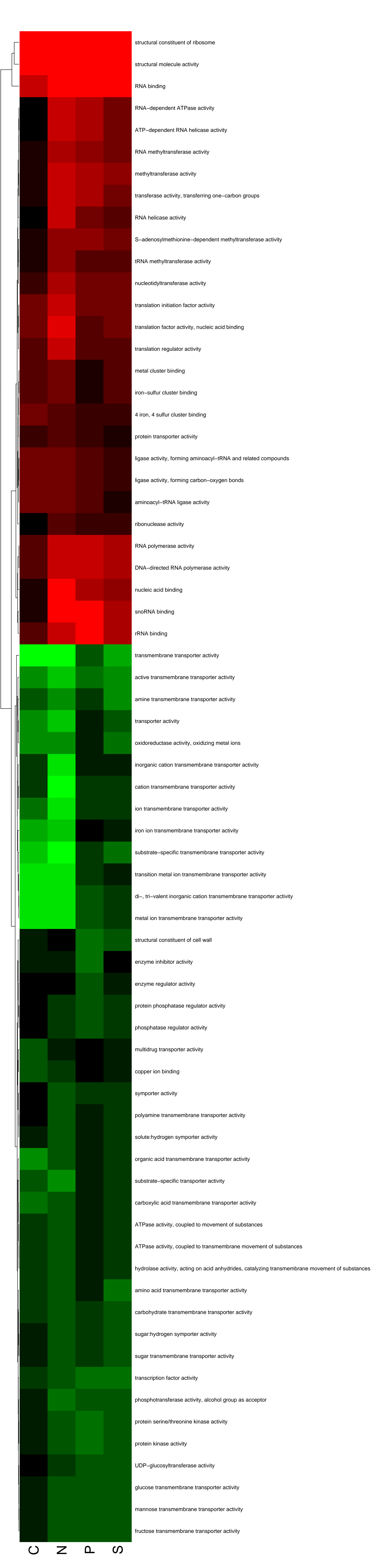

Supplement: Additional file 31 — Growth rate regulated GO molecular function terms (transcriptome). GO molecular function terms associated with up- (red) or down- (green) regulation of gene expression with changes in growth rate in one or more conditions (FDR < 1%). [file 1741-7007-8-68-S31.pdf]

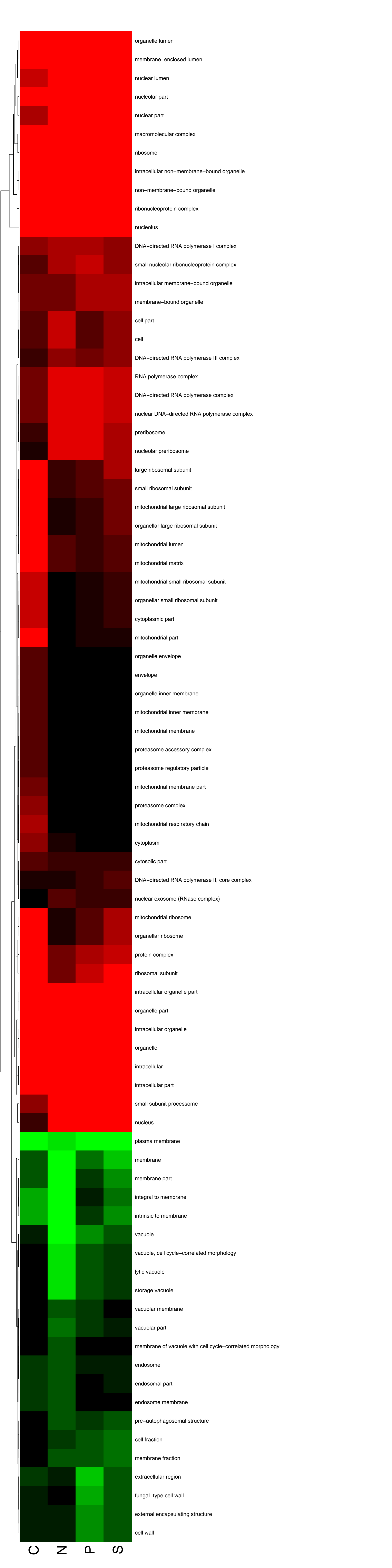

Supplement: Additional file 32 — Growth rate regulated GO cellular component terms (transcriptome). GO cellular component terms associated with up- (red) or down- (green) regulation of gene expression with changes in growth rate in one or more conditions (FDR < 1%). [file 1741-7007-8-68-S32.pdf]

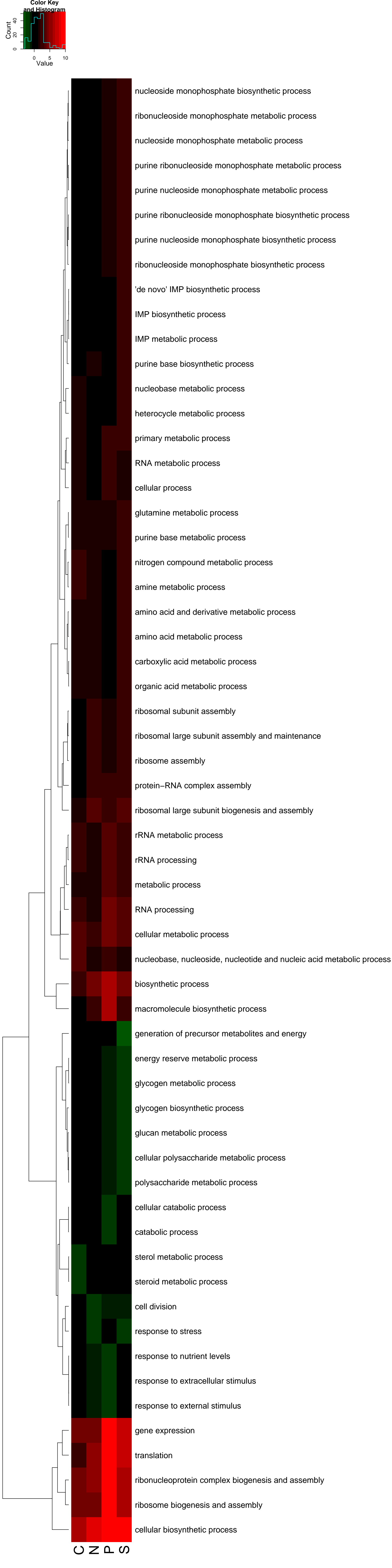

Supplement: Additional file 33 — Growth rate regulated GO biological process terms (proteome). GO biological process terms associated with up- (red) or down- (green) regulation of protein level with changes in growth rate in one or more conditions (FDR < 1%). [file 1741-7007-8-68-S33.pdf]

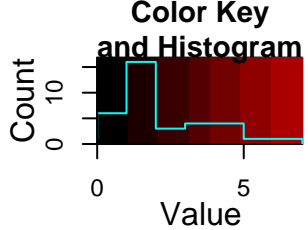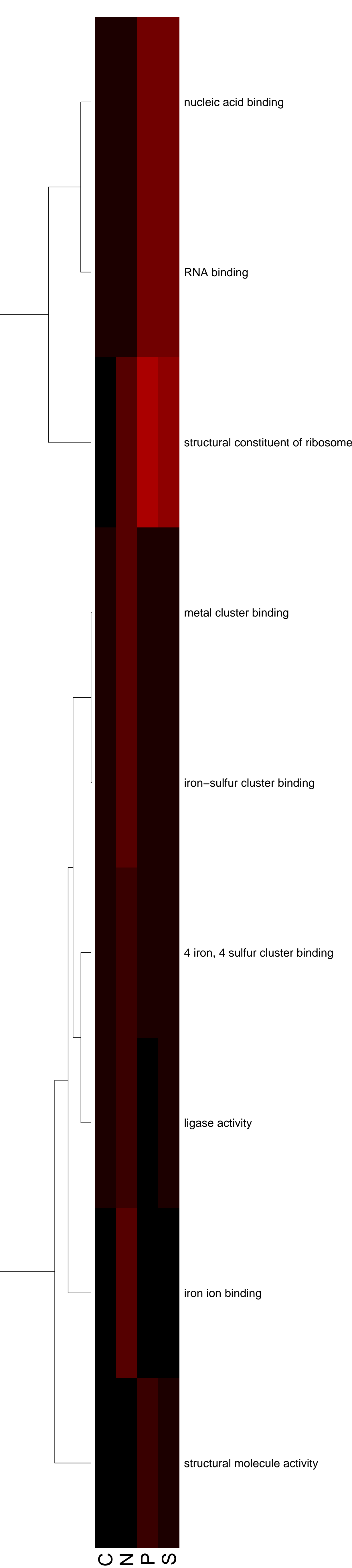

C Z P S

Supplement: Additional file 34 — Growth rate regulated GO molecular function terms (proteome). GO molecular function terms associated with up- (red) or down- (green) regulation of protein level with changes in growth rate in one or more conditions (FDR < 1%). [file 1741-7007-8-68-S34.pdf]

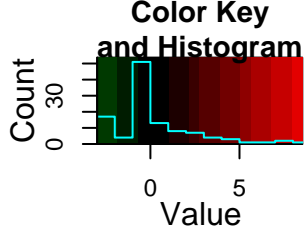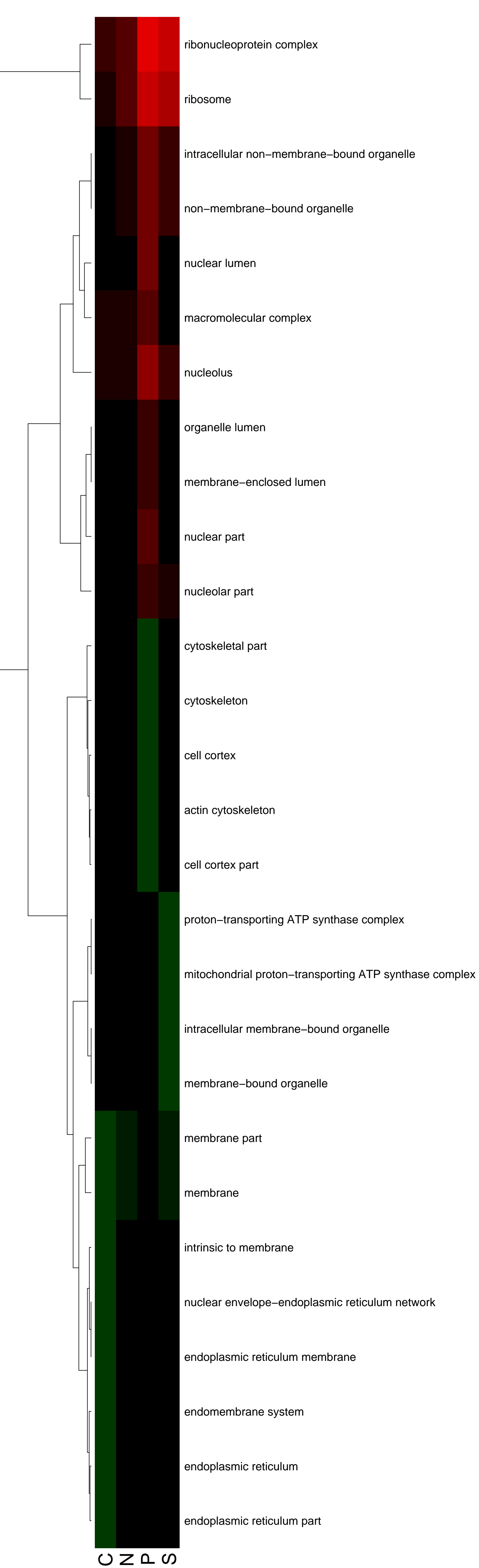

C Z P S

Supplement: Additional file 35 — Growth rate regulated GO cellular component terms (proteome). GO cellular component terms associated with up- (red) or down- (green) regulation of protein level with changes in growth rate in one or more conditions (FDR < 1%). [file 1741-7007-8-68-S35.pdf]

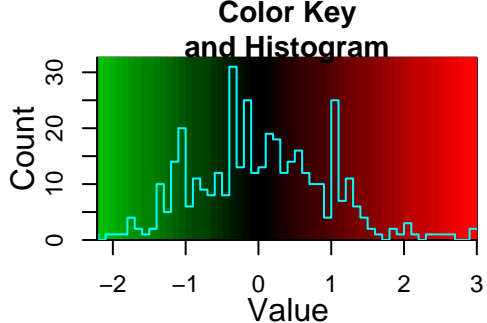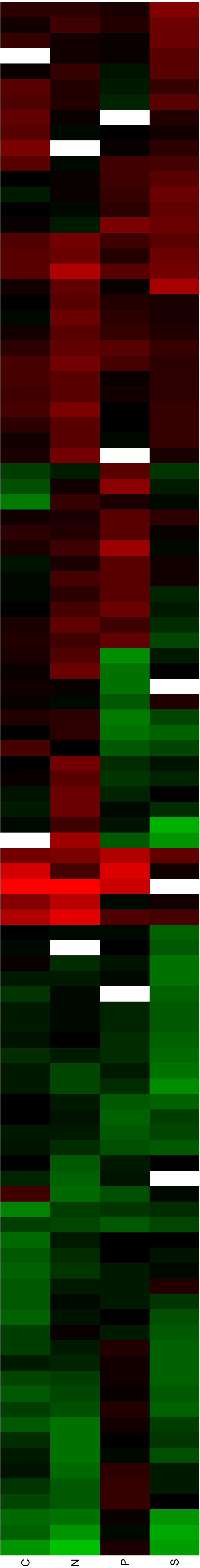

Supplement: Additional file 36 — ORFs under growth rate regulated post-transcriptional control. Individual genes that show significant changes in translational control efficiency between growth rates in each nutrient limitation. Colours indicate the log. ratio of translational control efficiency between the two growth rates. Only genes with a more than two-fold change in translational efficiency are shown. [file 1741-7007-8-68-S36.pdf]

# YPR184W

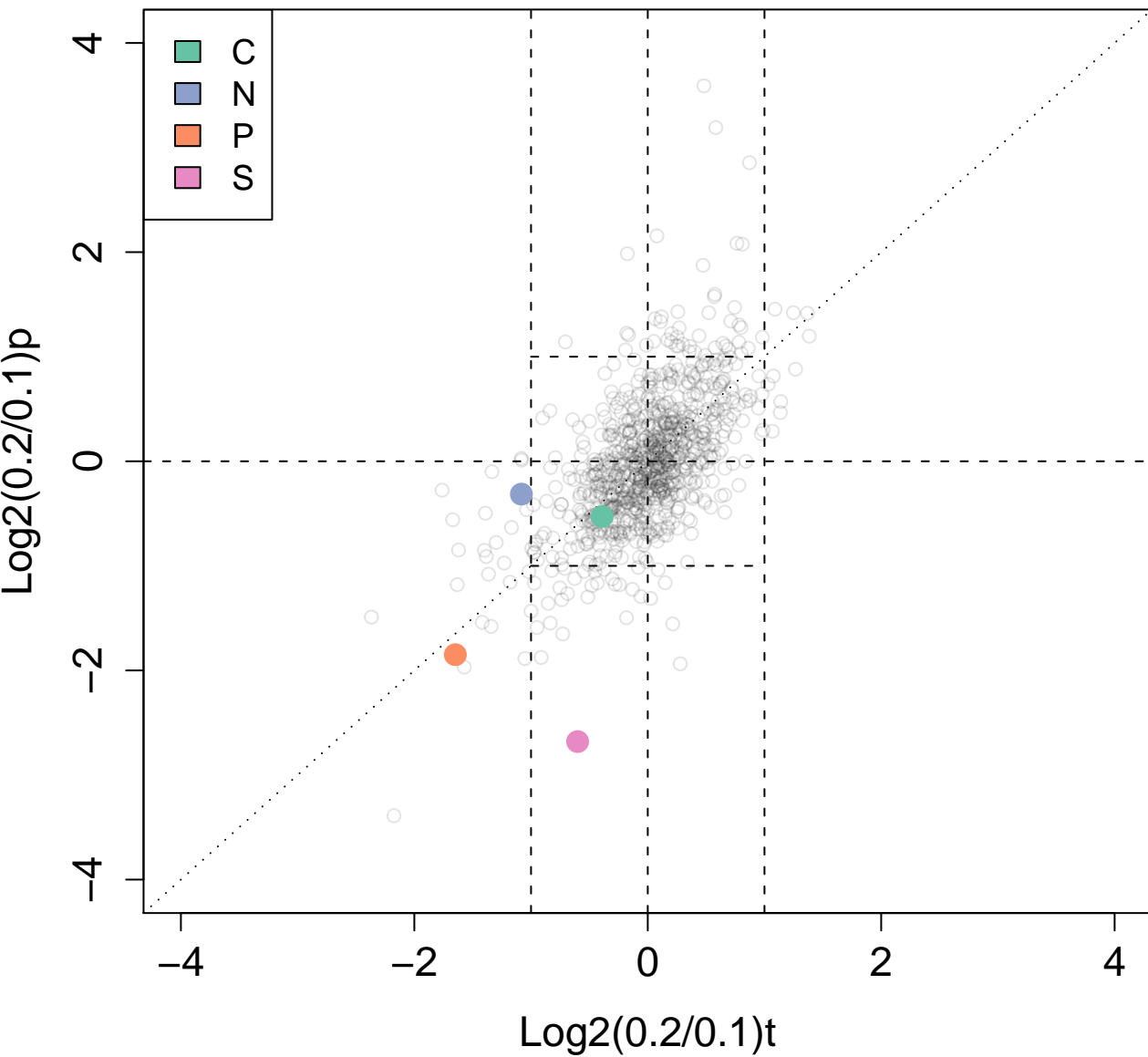

Supplement: Additional file 37 — Post-transcriptional control of YPR184W. Protein and transcript log. fold changes for the shift from D = 0.1 h-1 to D = 0.2 h-1 in each nutrient-limiting condition for YPR184W. [file 1741-7007-8-68-S37.pdf]

# YLR034C

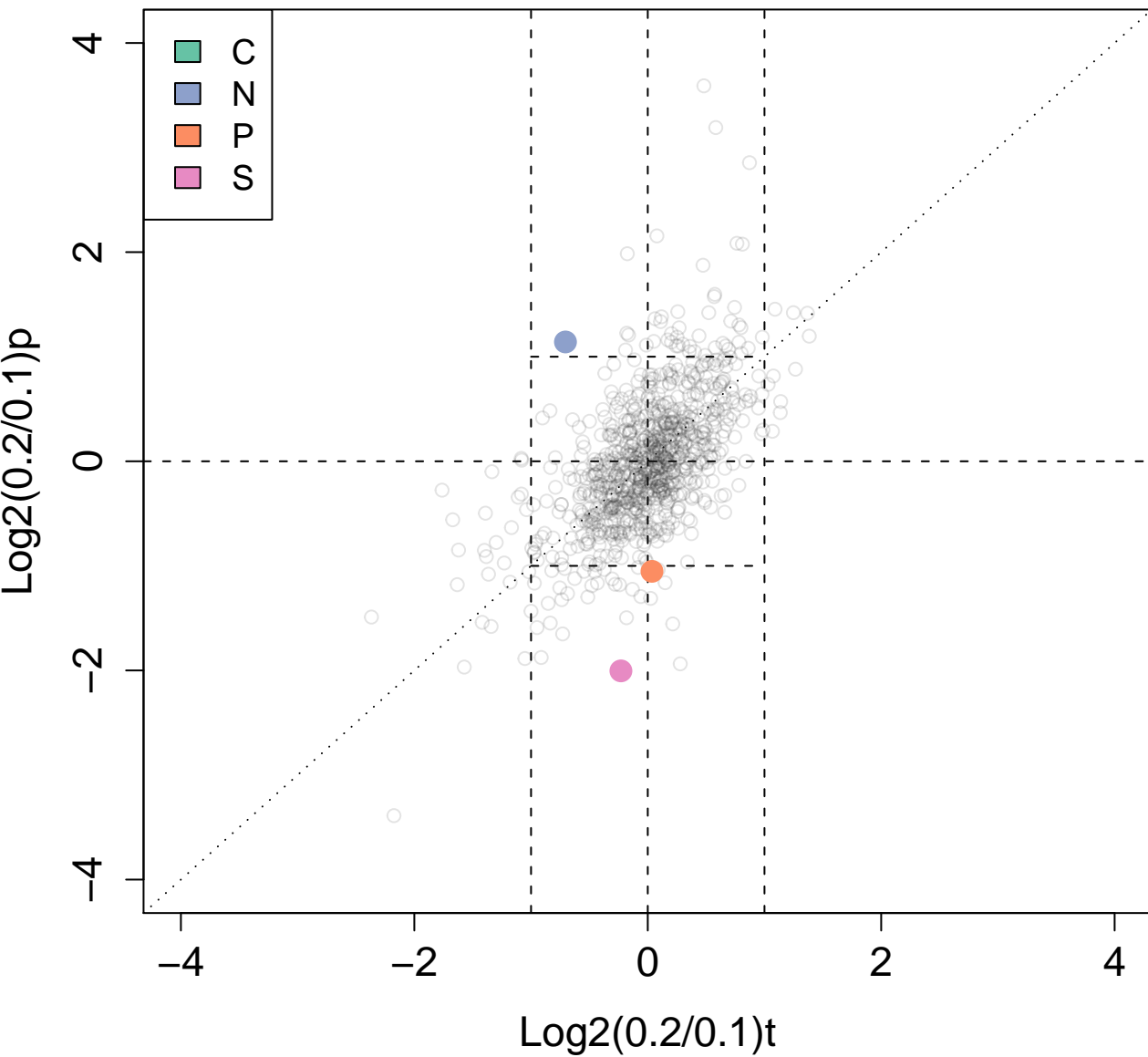

Supplement: Additional file 38 — Post-transcriptional control of YLR034C. Protein and transcript log. fold changes for the shift from D = 0.1 h-1 to D = 0.2 h-1 in each nutrient-limiting condition for YLR034C. [file 1741-7007-8-68-S38.pdf]

# YLR285W

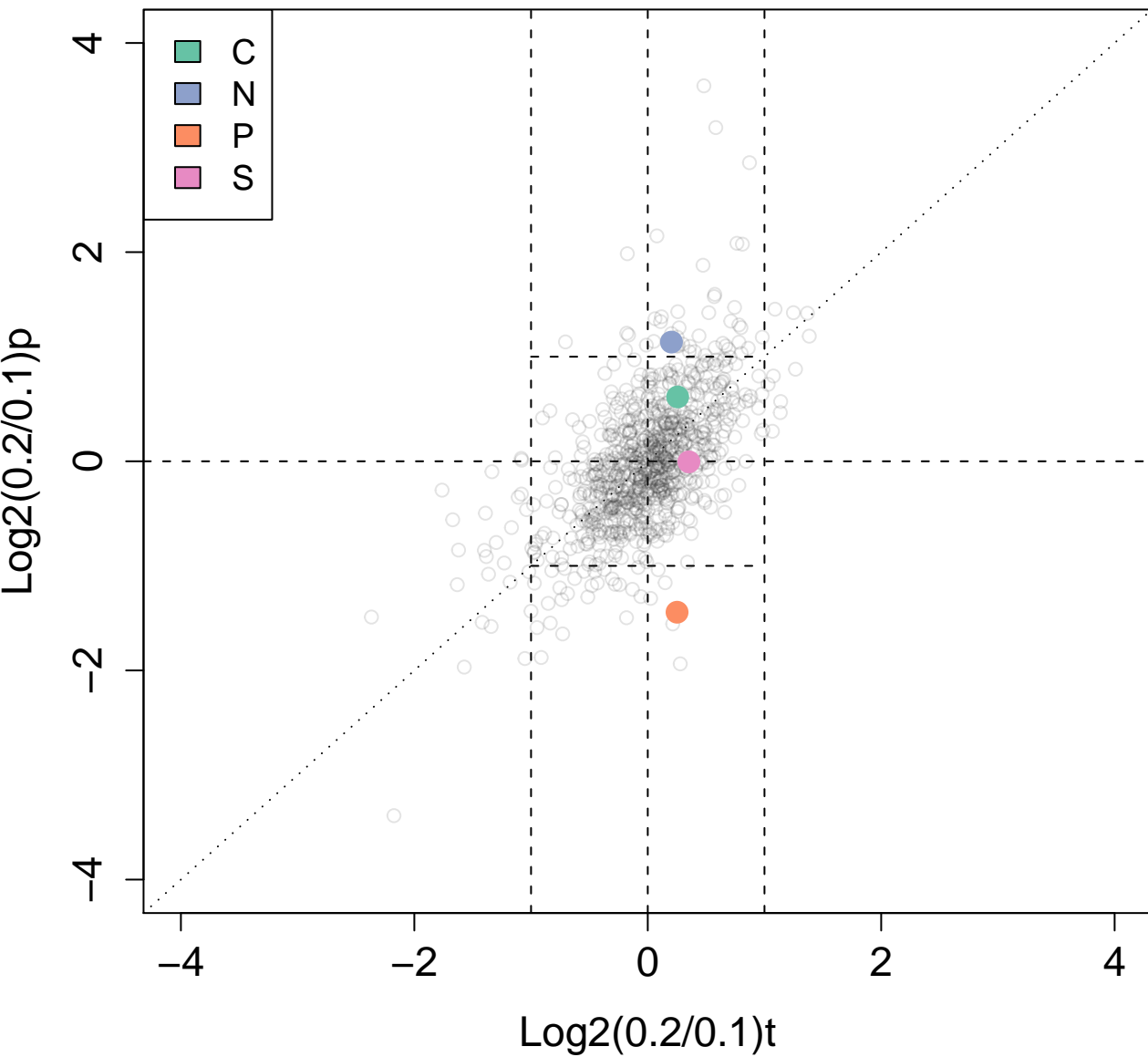

Supplement: Additional file 39 — Post-transcriptional control of YLR285W. Protein and transcript log. fold changes for the shift from D = 0.1 h-1 to D = 0.2 h-1 in each nutrient-limiting condition for YLR285W. [file 1741-7007-8-68-S39.pdf]

# YPR160W

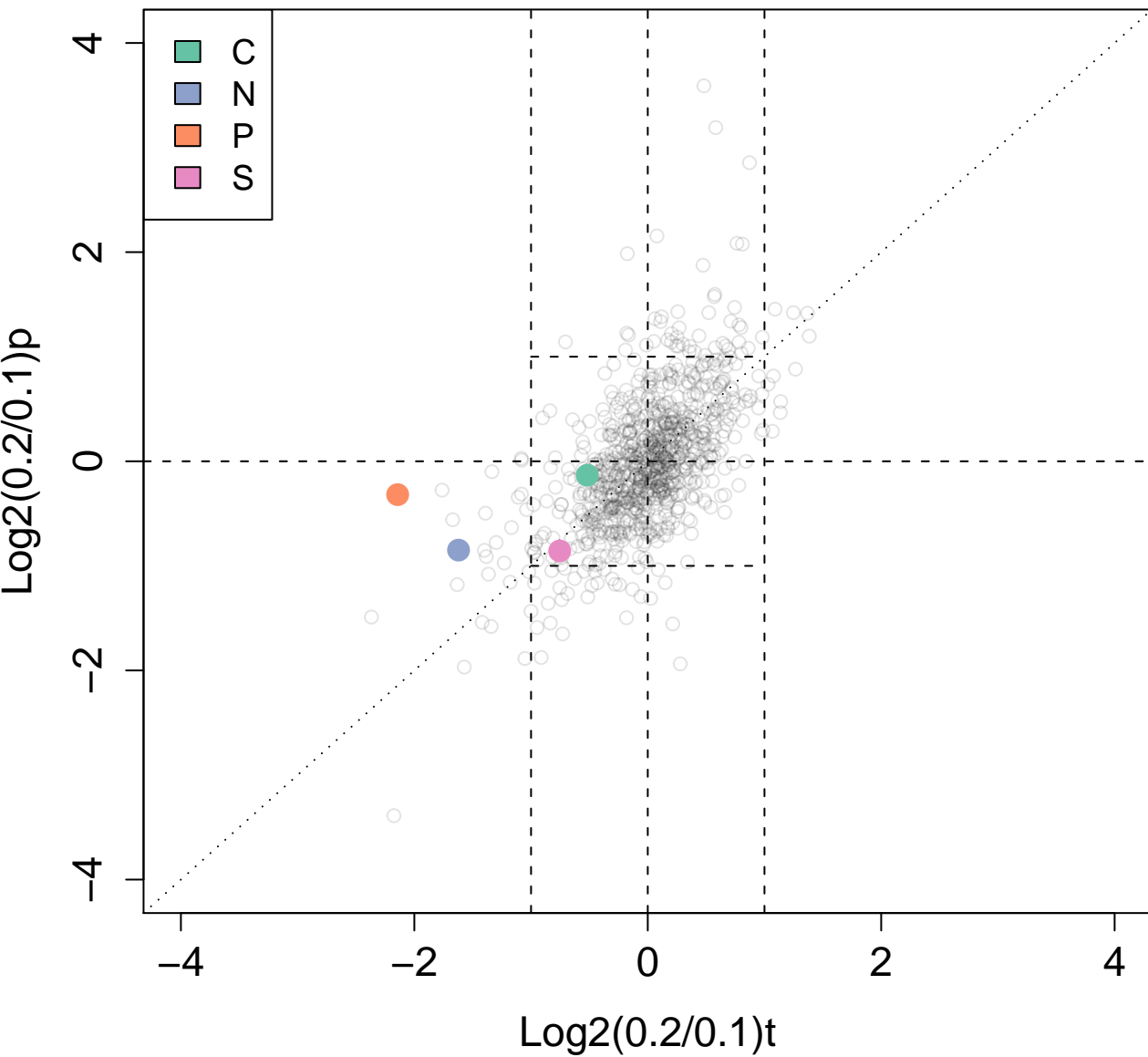

Supplement: Additional file 40 — Post-transcriptional control of YPR160W. Protein and transcript log. fold changes for the shift from D = 0.1 h-1 to D = 0.2 h-1 in each nutrient-limiting condition for YPR160W. [file 1741-7007-8-68-S40.pdf]

# YGR175C

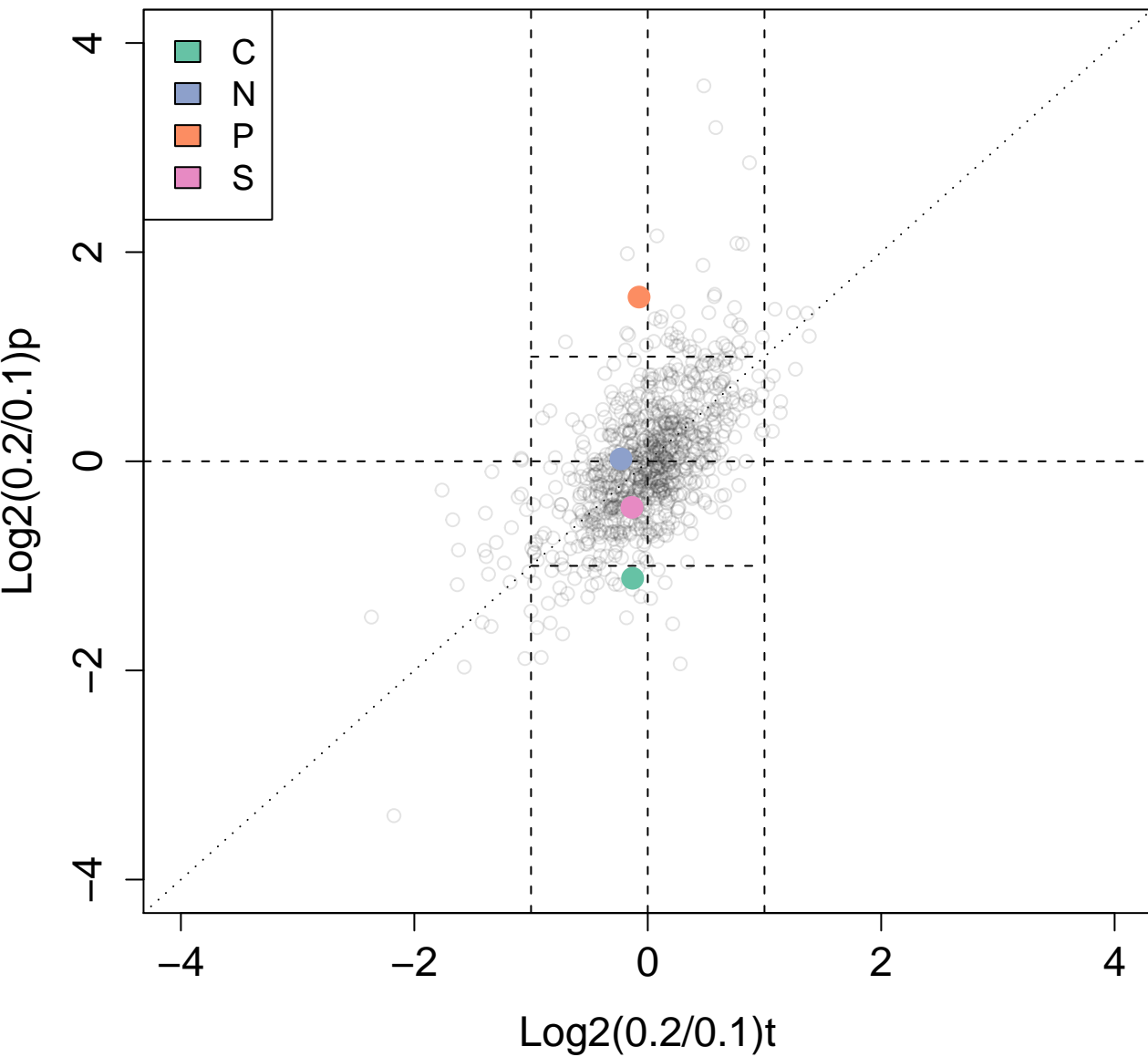

Supplement: Additional file 41 — Post-transcriptional control of YGR175C. Protein and transcript log. fold changes for the shift from D = 0.1 h-1 to D = 0.2 h-1 in each nutrient-limiting condition for YGR175C. [file 1741-7007-8-68-S41.pdf]

# YOR303W

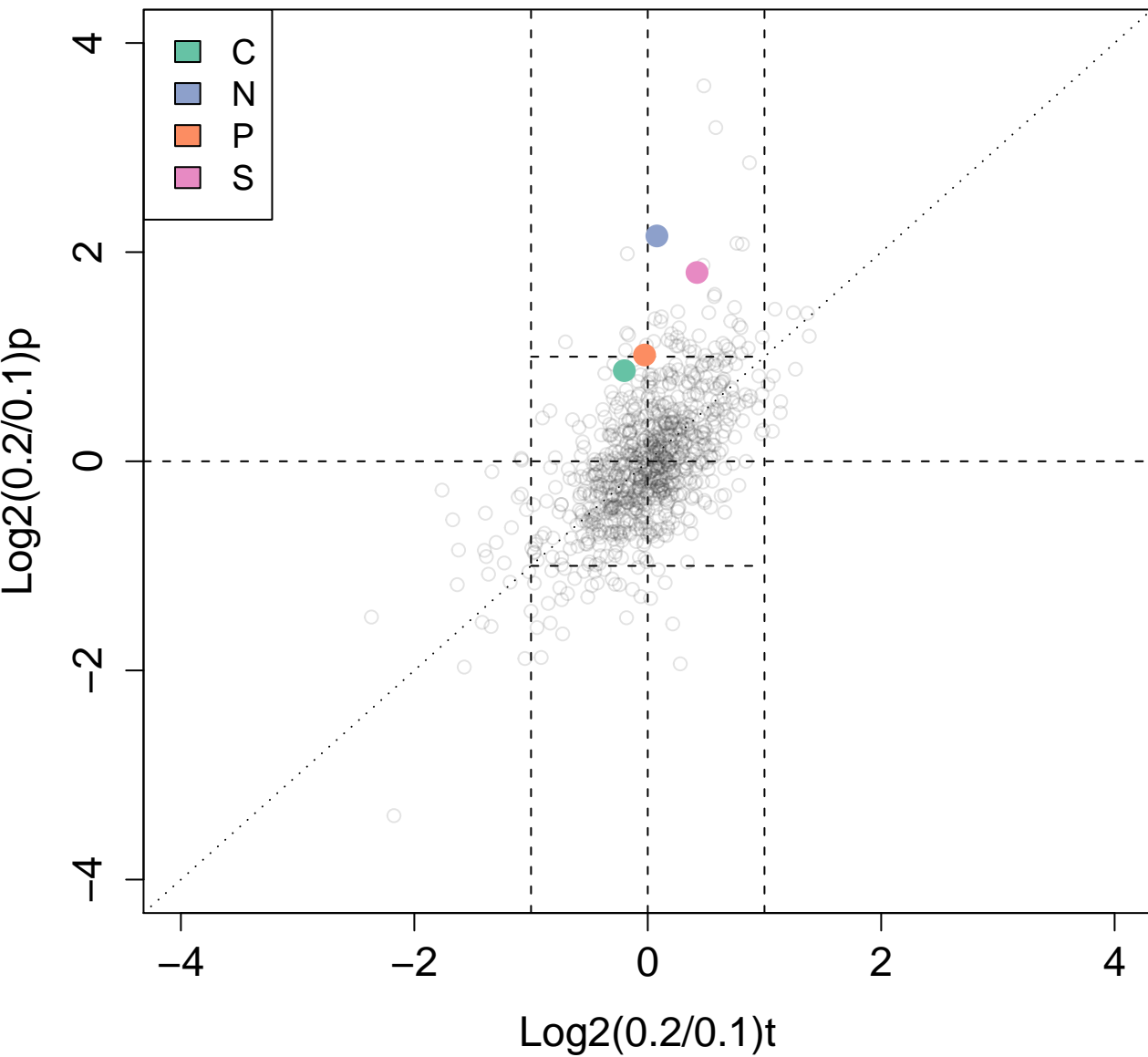

Supplement: Additional file 42 — Post-transcriptional control of YOR303W. Protein and transcript log. fold changes for the shift from D = 0.1 h-1 to D = 0.2 h-1 in each nutrient-limiting condition for YOR303W. [file 1741-7007-8-68-S42.pdf]

# YDL171C

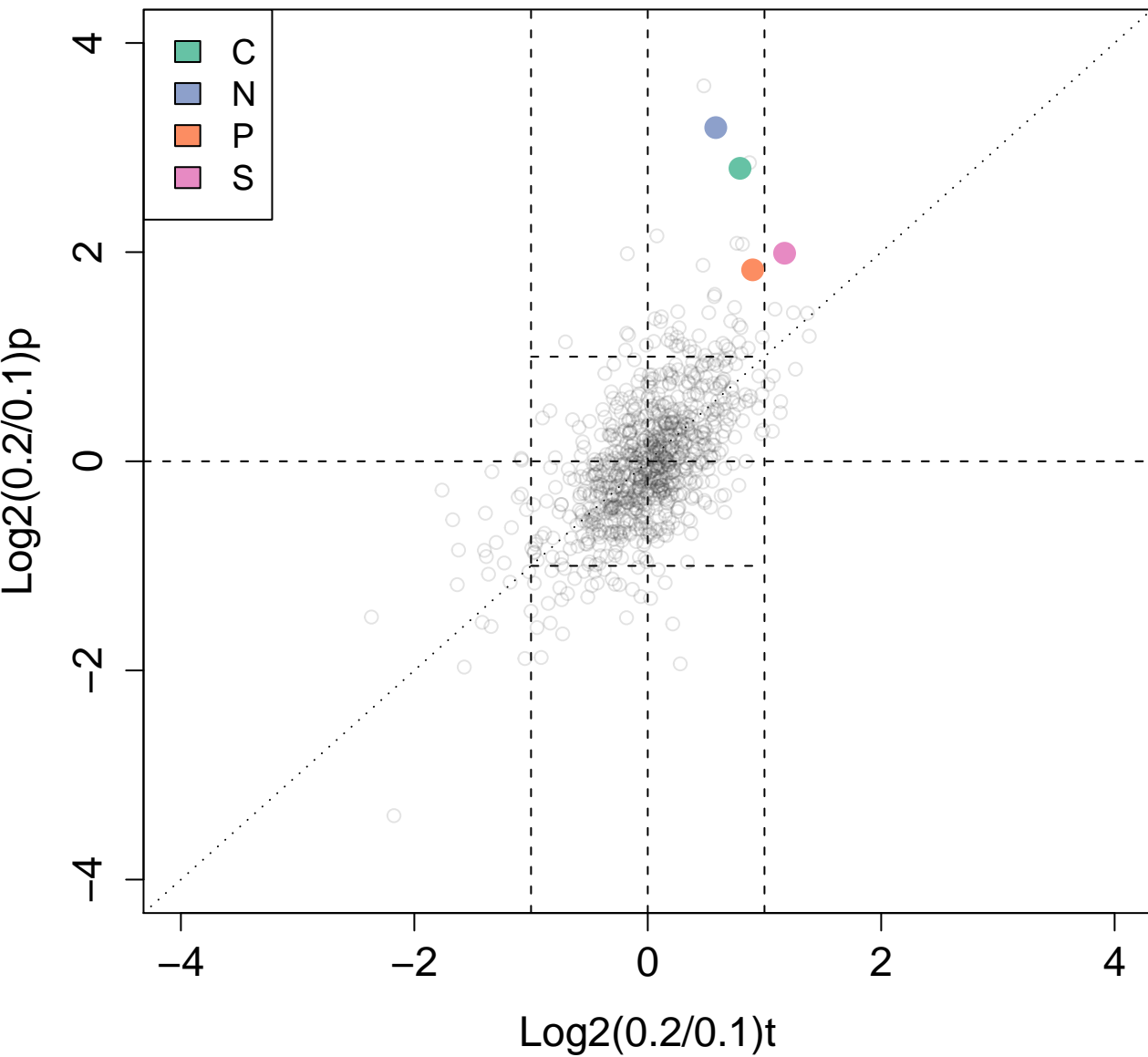

Supplement: Additional file 43 — Post-transcriptional control of YDL171C. Protein and transcript log. fold changes for the shift from D = 0.1 h-1 to D = 0.2 h-1 in each nutrient-limiting condition for YDL171C. [file 1741-7007-8-68-S43.pdf]

# YPL240C

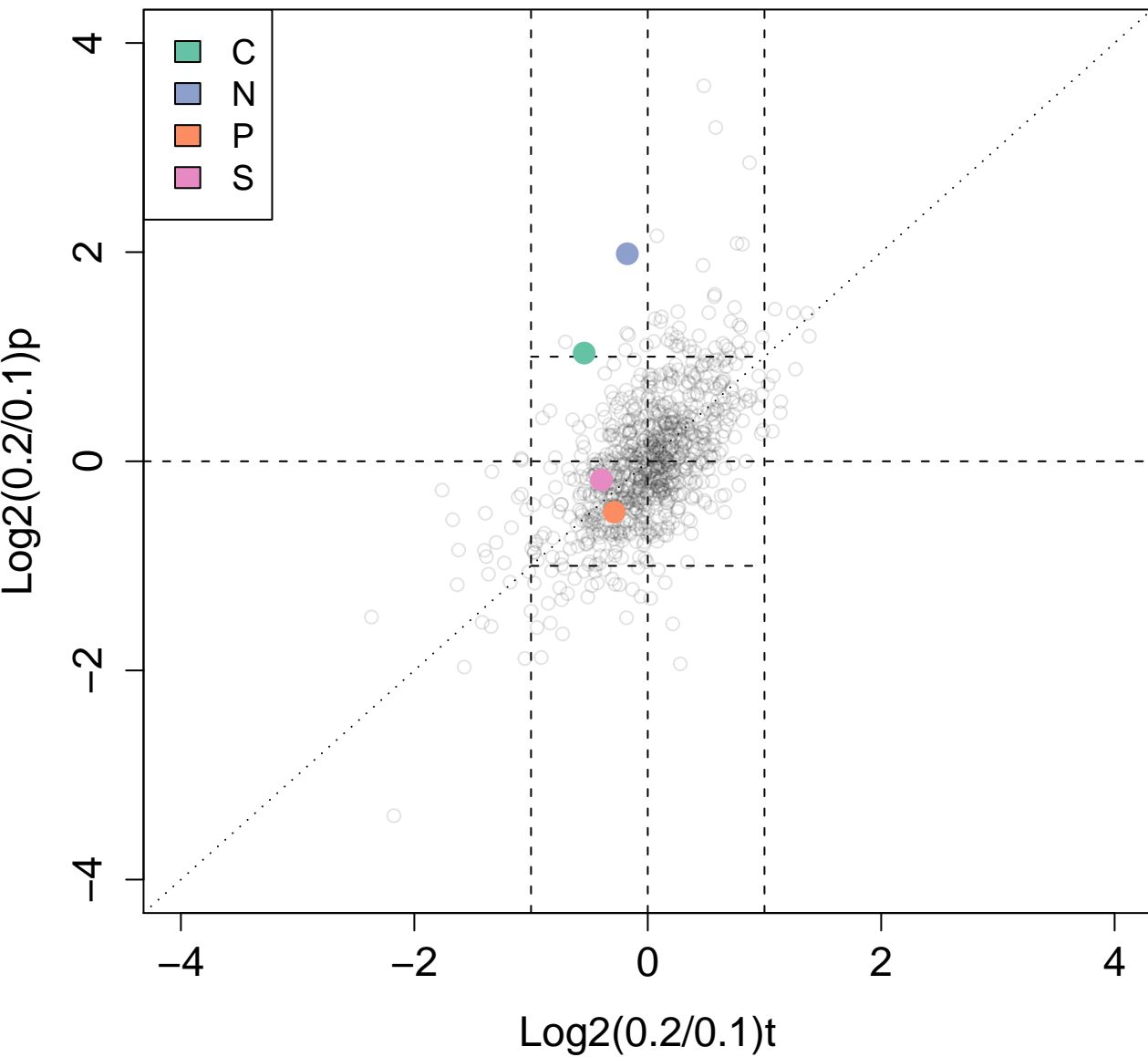

Supplement: Additional file 44 — Post-transcriptional control of YPL240C. Protein and transcript log. fold changes for the shift from D = 0.1 h-1 to D = 0.2 h-1 in each nutrient-limiting condition for YPL240C. [file 1741-7007-8-68-S44.pdf]

# YCL011C

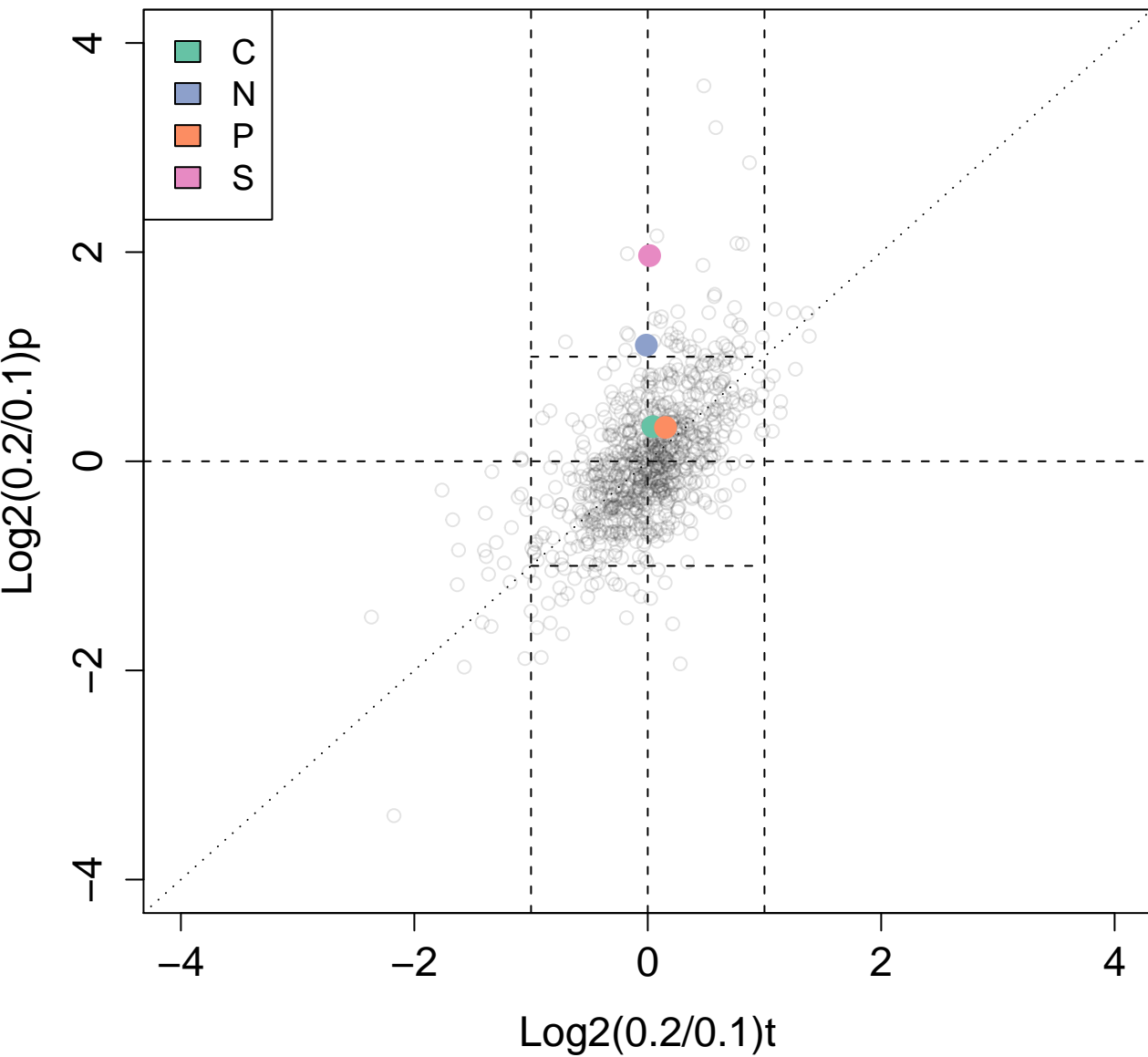

Supplement: Additional file 45 — Post-transcriptional control of YCL011C. Protein and transcript log. fold changes for the shift from D = 0.1 h-1 to D = 0.2 h-1 in each nutrient-limiting condition for YCL011C. [file 1741-7007-8-68-S45.pdf]

# YGR087C

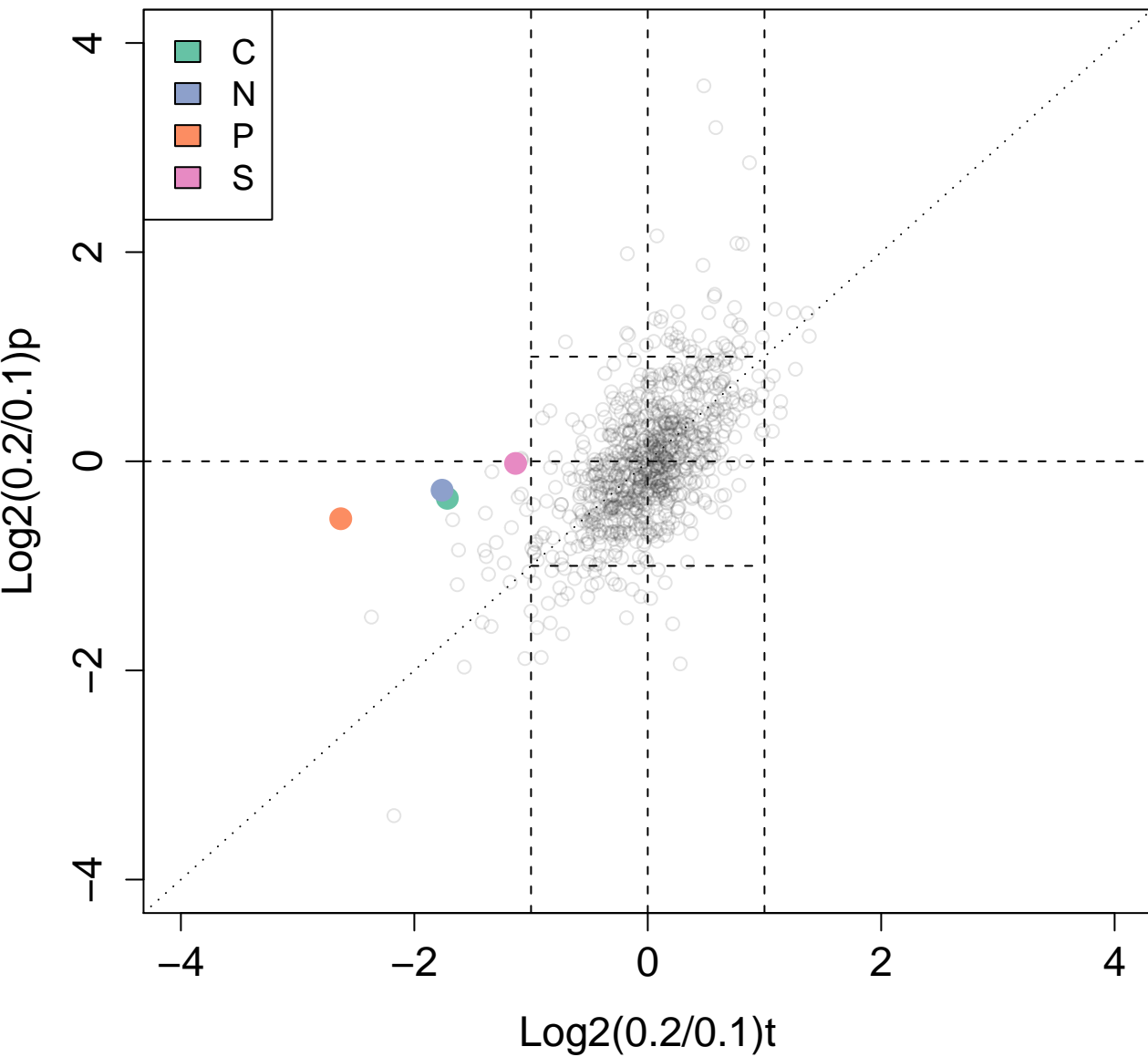

Supplement: Additional file 46 — Post-transcriptional control of YGR087C. Protein and transcript log. fold changes for the shift from D = 0.1 h-1 to D = 0.2 h-1 in each nutrient-limiting condition for YGR087C. [file 1741-7007-8-68-S46.pdf]

# YBR072W

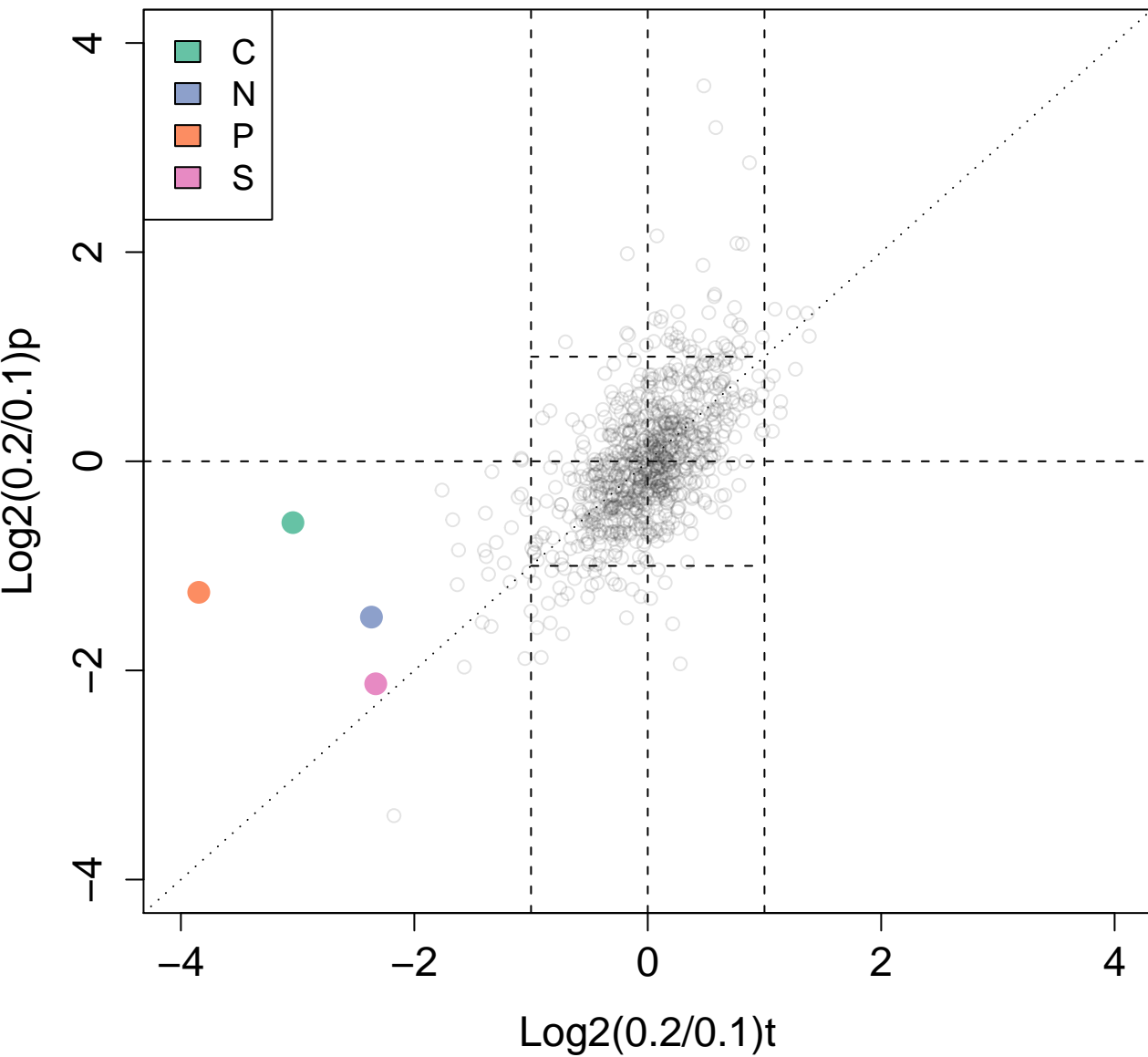

Supplement: Additional file 47 — Post-transcriptional control of YBR072W. Protein and transcript log. fold changes for the shift from D = 0.1 h-1 to D = 0.2 h-1 in each nutrient-limiting condition for YBR072W. [file 1741-7007-8-68-S47.pdf]

# YMR083W

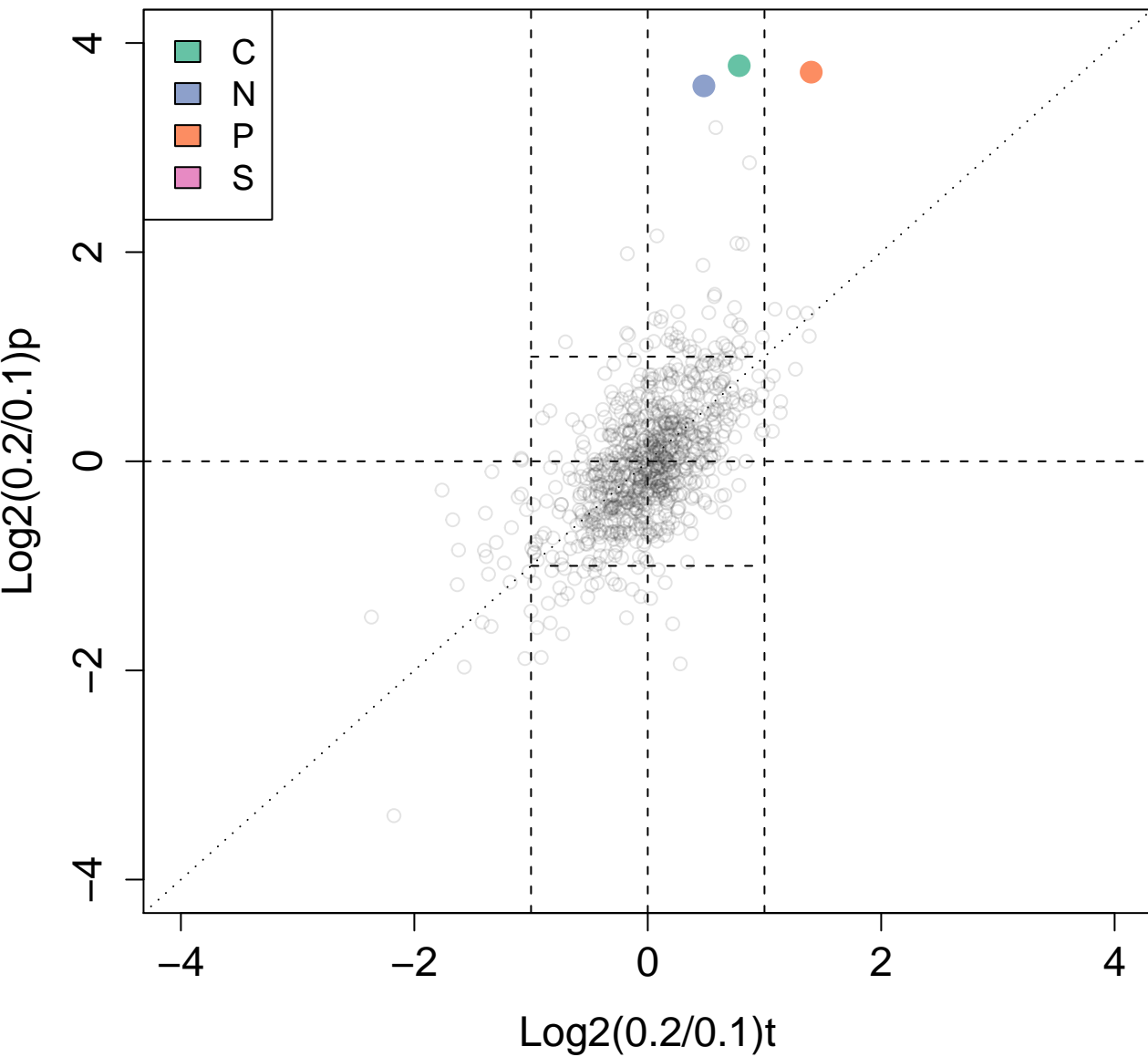

Supplement: Additional file 48 — Post-transcriptional control of YMR083W. Protein and transcript log. fold changes for the shift from D = 0.1 h-1 to D = 0.2 h-1 in each nutrient-limiting condition for YMR083W. [file 1741-7007-8-68-S48.pdf]

# YOL151W

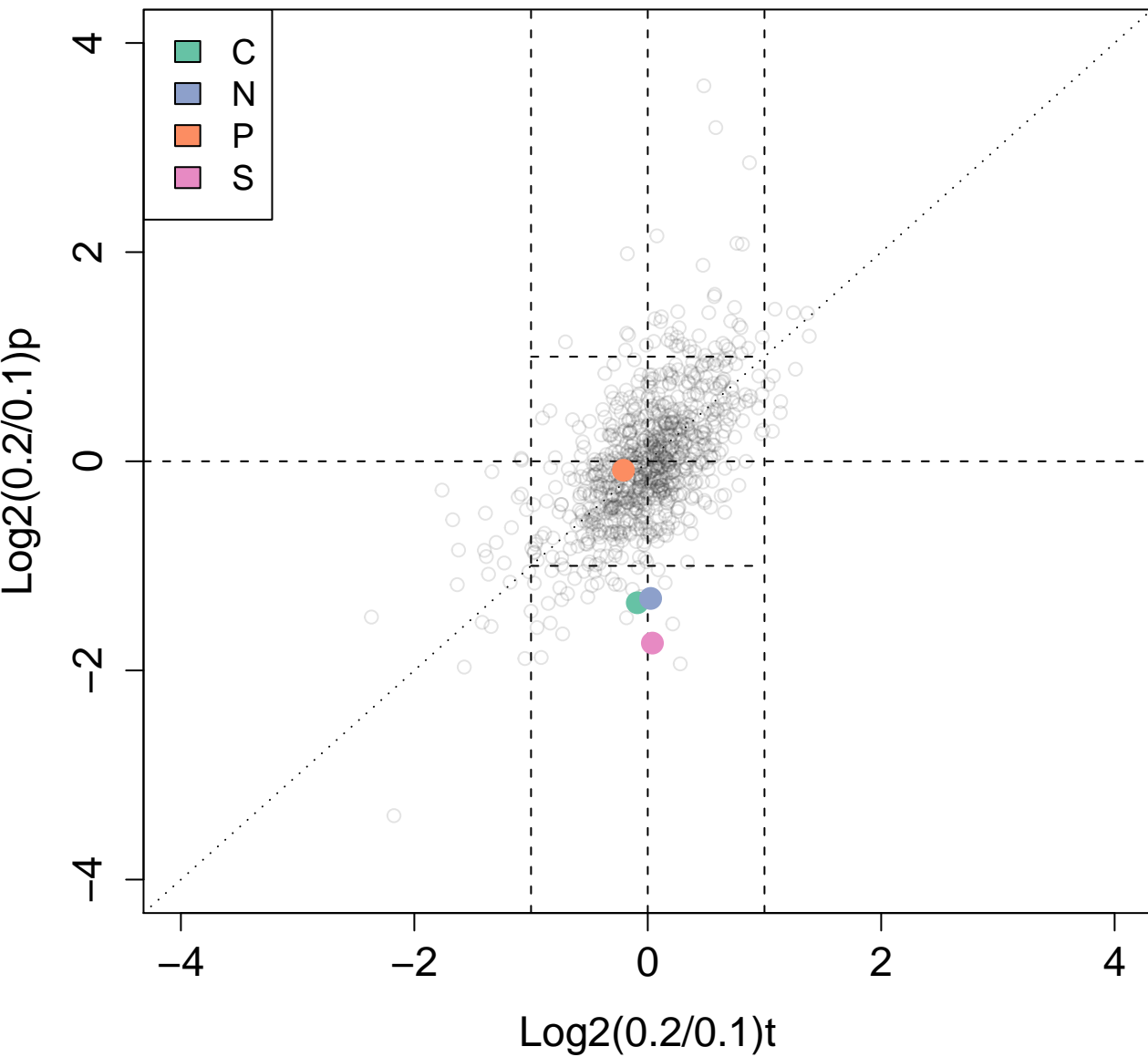

Supplement: Additional file 49 — Post-transcriptional control of YOL151W. Protein and transcript log. fold changes for the shift from D = 0.1 h-1 to D = 0.2 h-1 in each nutrient-limiting condition for YOL151W. [file 1741-7007-8-68-S49.pdf]

# YER021W

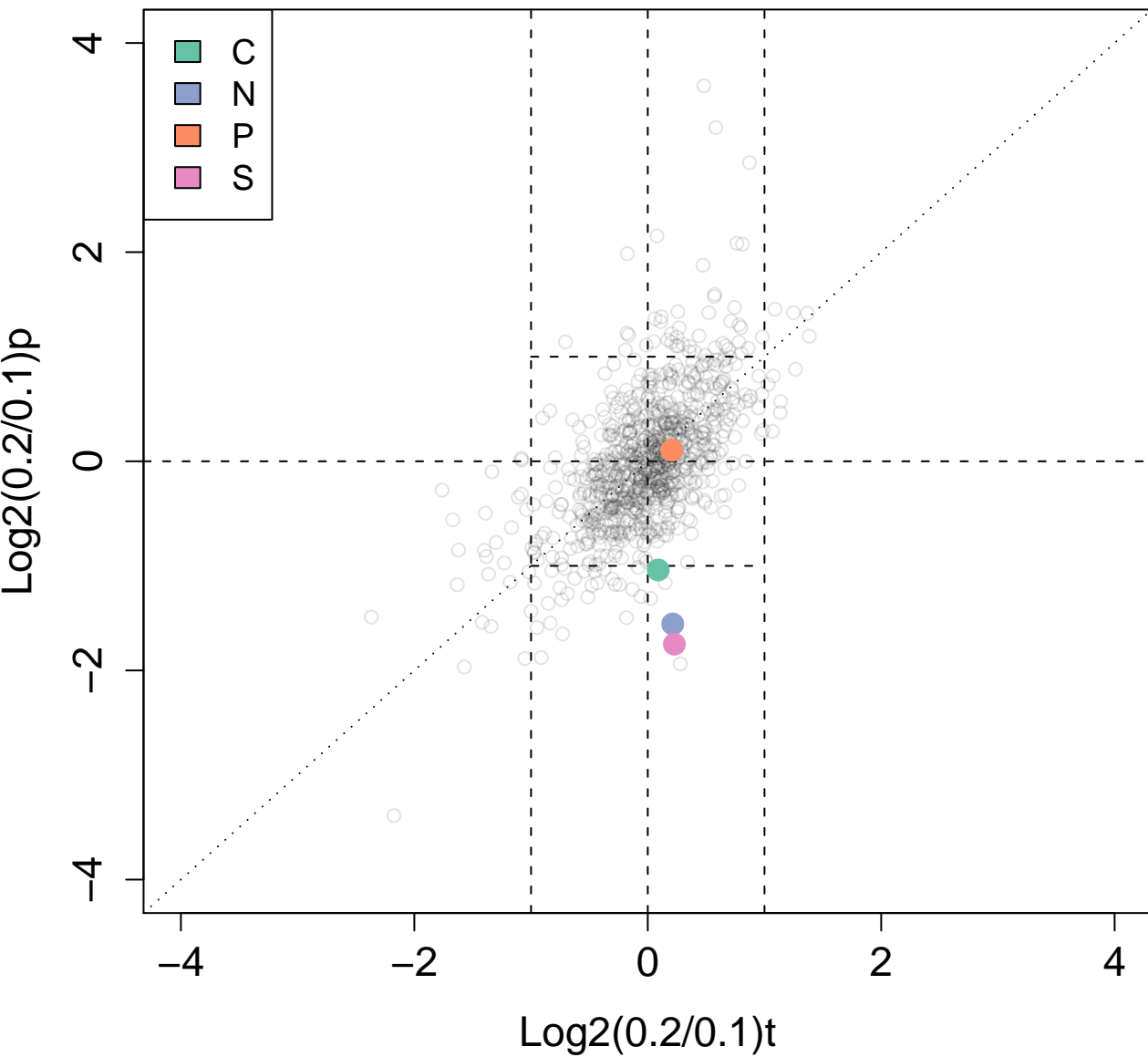

Supplement: Additional file 50 — Post-transcriptional control of YER021W. Protein and transcript log. fold changes for the shift from D = 0.1 h-1 to D = 0.2 h-1 in each nutrient-limiting condition for YER021W. [file 1741-7007-8-68-S50.pdf]

# YGL068W

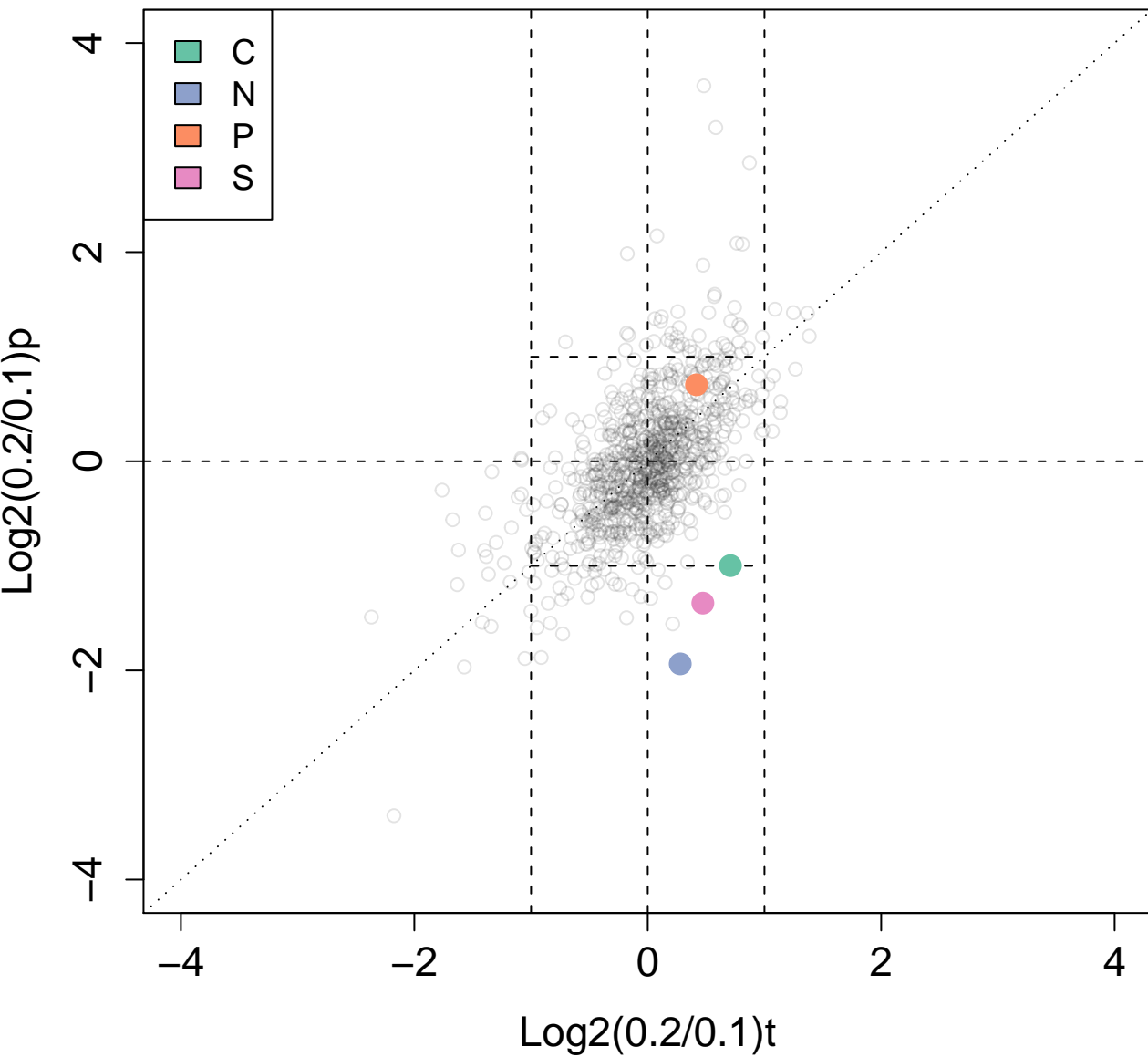

Supplement: Additional file 51 — Post-transcriptional control of YGL068W. Protein and transcript log. fold changes for the shift from D = 0.1 h-1 to D = 0.2 h-1 in each nutrient-limiting condition for YGL068W. [file 1741-7007-8-68-S51.pdf]

# YDR155C

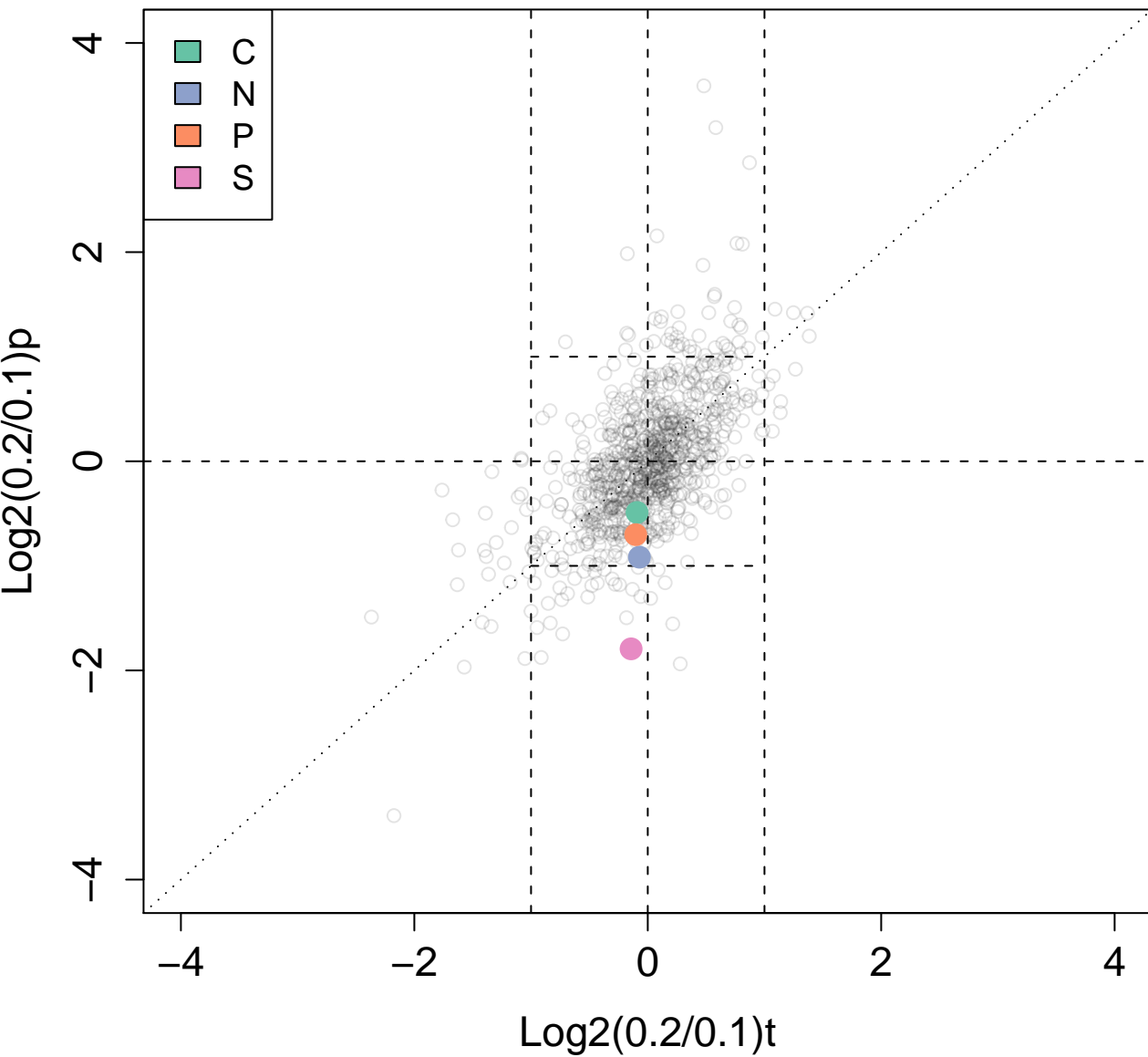

Supplement: Additional file 52 — Post-transcriptional control of YDR155C. Protein and transcript log. fold changes for the shift from D = 0.1 h-1 to D = 0.2 h-1 in each nutrient-limiting condition for YDR155C. [file 1741-7007-8-68-S52.pdf]

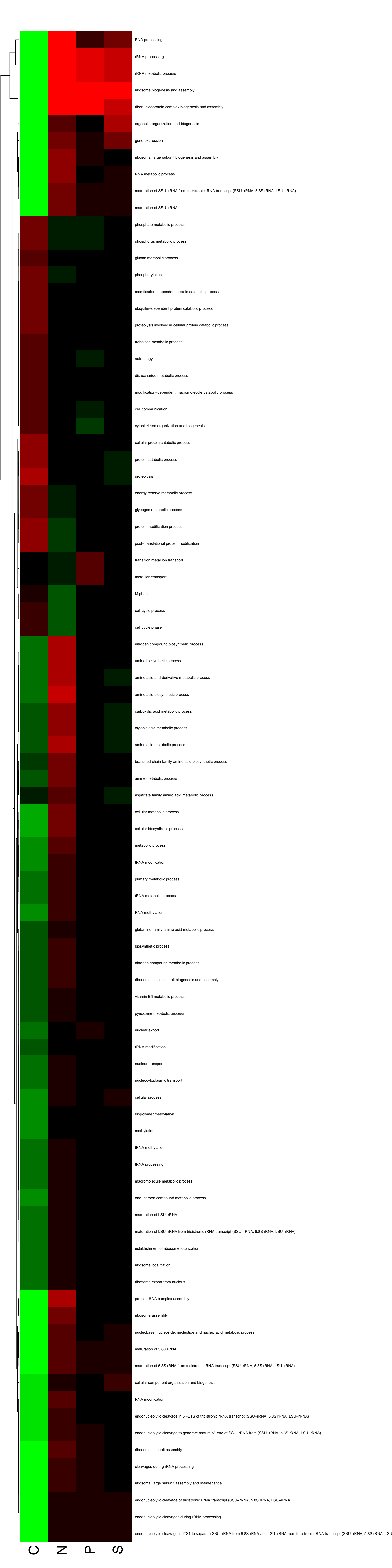

Supplement: Additional file 58 — Nutrient specific growth rate regulated GO biological process terms (transcriptome). GO biological process terms associated with up- (red) or down- (green) regulation of gene expression with increasing growth rate in one or more conditions relative to the overall trend (FDR < 1%). [file 1741-7007-8-68-S58.pdf]

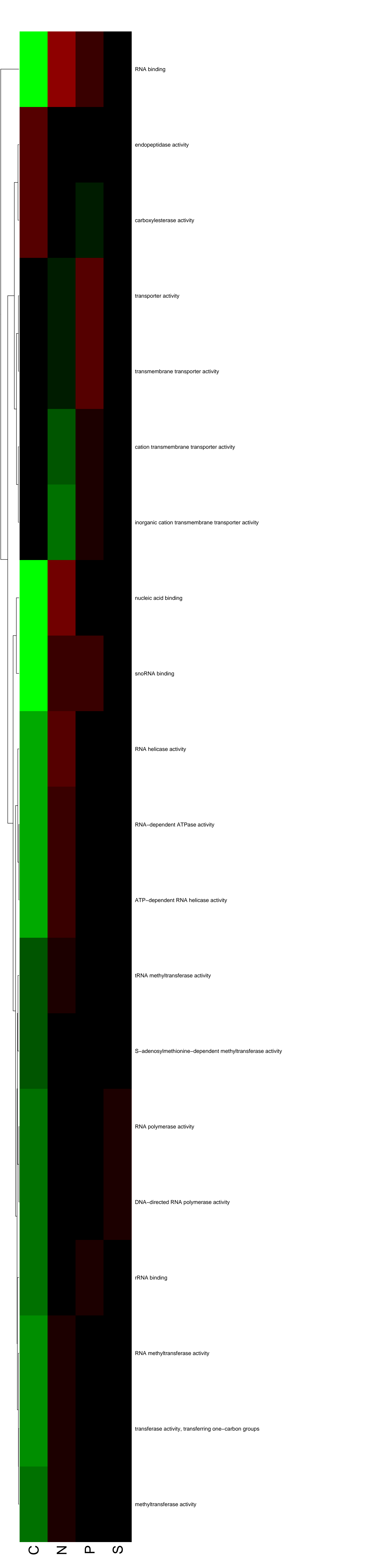

Supplement: Additional file 59 — Nutrient specific growth rate regulated GO molecular function terms (transcriptome). GO molecular function terms associated with up- (red) or down- (green) regulation of gene expression with increasing growth rate in one or more conditions relative to the overall trend (FDR < 1%). [file 1741-7007-8-68-S59.pdf]

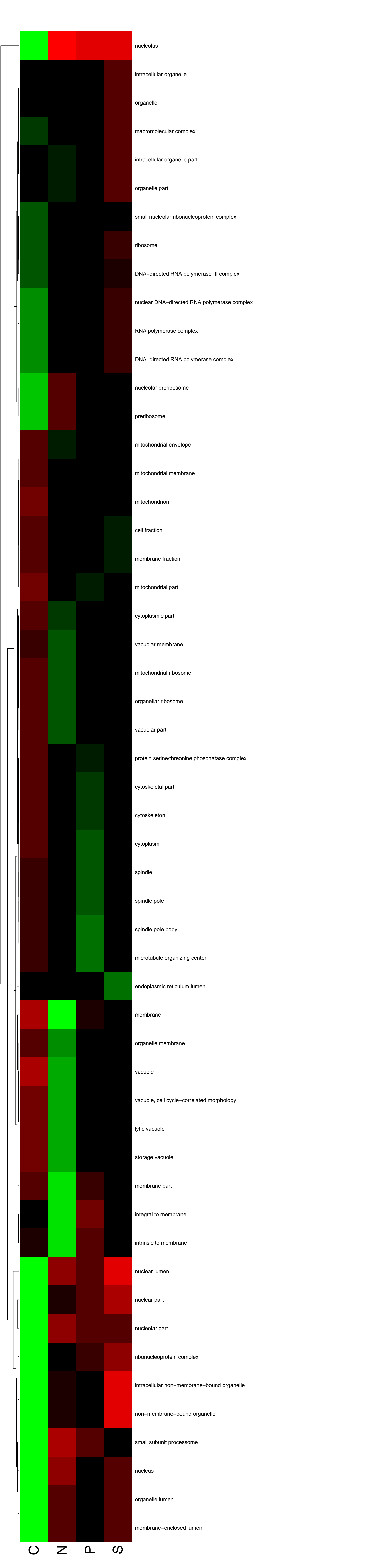

Supplement: Additional file 60 — Nutrient specific growth rate regulated GO cellular component terms (transcriptome). GO cellular component terms associated with up- (red) or down- (green) regulation of gene expression with increasing growth rate in one or more conditions relative to the overall trend (FDR < 1%). [file 1741-7007-8-68-S60.pdf]

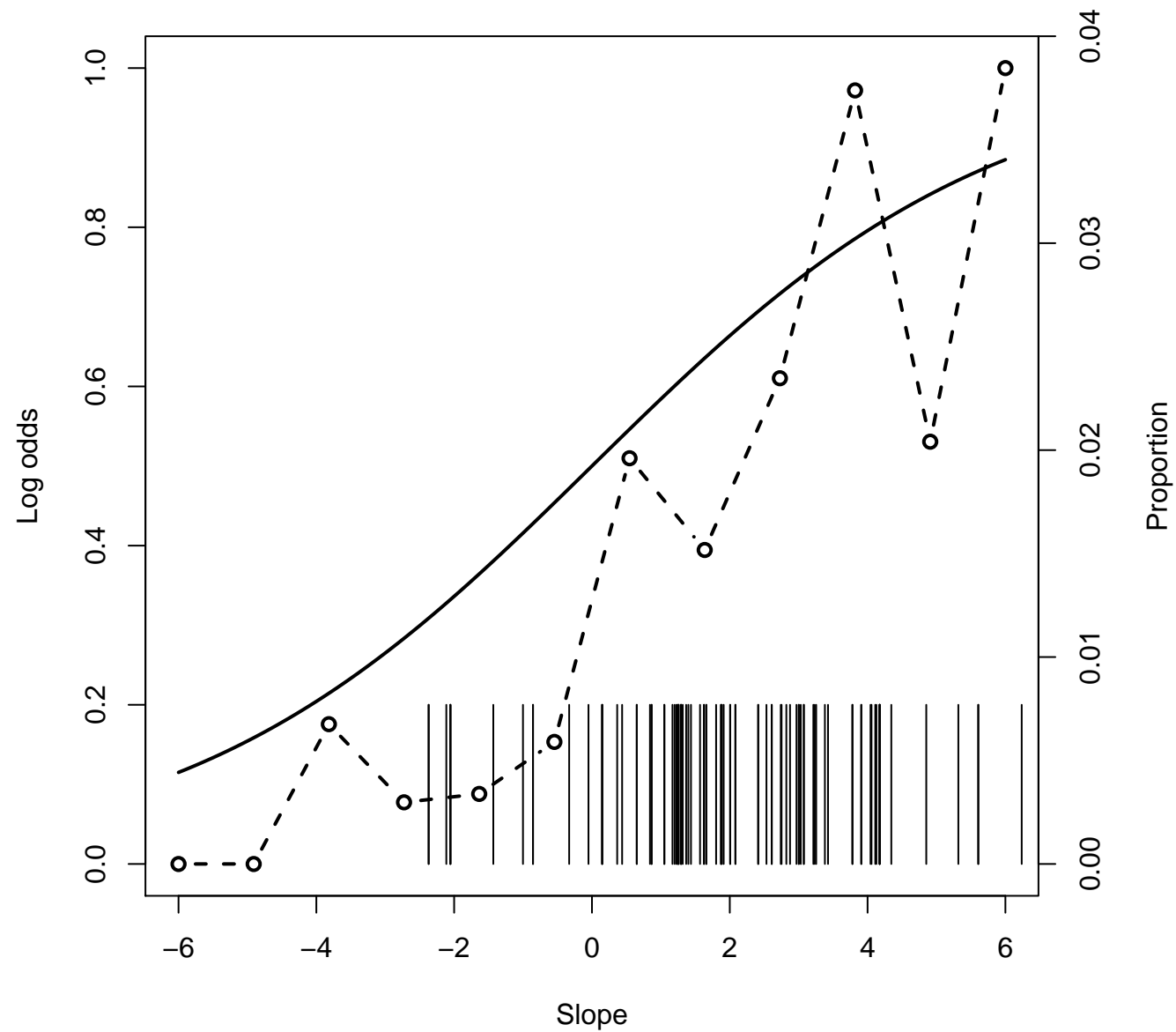

Supplement: Additional file 61 — Logistic regression example. Association of positive slopes of regression with the ribosome biogenesis GO term. Slopes are calculated for each gene from a linear regression of gene expression against growth rate. Vertical tick marks show the slopes of ribosome biogenesis annotated genes. The proportion of all genes at a given slope that are annotated with the term is shown with the dashed line. The solid line shows the fitted logistic regression. [file 1741-7007-8-68-S61.pdf]
