# Supplementary material for: Nutrient control of eukaryote cell growth: a systems biology study in yeast
Source: BMC Biol. 2010 May 24;8:68. doi: 10.1186/1741-7007-8-68 (PMC2895586; doi:10.1186/1741-7007-8-68)
Supplement: Additional file 53 — Physiological parameters. Specific rates of glucose comsumption (qgluc) and ethanol production (qethanol) from the chemostat series under specific nutrient-limiting conditions. [file 1741-7007-8-68-S53.doc]

|  |  |  |  |
| --- | --- | --- | --- |
| Dilution rate (h-1) | 0.07 | 0.10 | 0.20 |
|  |  |  |  |
|  |  |  |  |
| C limitation |  |  |  |
| q_gluc (mmol g-1 h-1)_ | 1.1 | 1.7 | 5.8 |
| q_ethanol (mmol g-1 h-1)_ | <0.08 | 0.2 | 11.1 |
|  |  |  |  |
| N limitation |  |  |  |
| q_gluc (mmol g-1 h-1)_ | 3.8 | 4.1 | 7.4 |
| q_ethanol (mmol g-1 h-1)_ | 5.7 | 7.1 | 16.2 |
|  |  |  |  |
| P limitation |  |  |  |
| q_gluc (mmol g-1 h-1)_ | 4.2 | 3.9 | 16.0 |
| q_ethanol (mmol g-1 h-1)_ | 8.0 | 11.0 | 27.4 |
|  |  |  |  |
| S limitation |  |  |  |
| q_gluc (mmol g-1 h-1)_ | 2.8 | 3.7 | 4.6 |
| q_ethanol (mmol g-1 h-1)_ | 6.3 | 4.2 | >8.0 |
|  |  |  |  |

Specific rates of glucose comsumption (q_gluc_) and ethanol production (q_ethanol_) from enzymatic analyses (Sigma and Boehringer kits) of *S. cerevisiae* chemostat series under specific nutrient-limiting conditions (C-, N-, P-, S-limited cultures) (Castrillo et al., 2007).
